# Supplementary material for: Wnt-presenting materials sustain H3K14-acetylation in human skeletal stem cells for tissue engineering and bone repair
Source: NPJ Regen Med. 2026 Mar 17;11:27. doi: 10.1038/s41536-026-00467-w (PMC13250124; doi:10.1038/s41536-026-00467-w)
Supplement: Supplementary file 1 — Supplementary Information [file 41536_2026_467_MOESM1_ESM.pdf]

<u>**Supplementary Figures**</u>

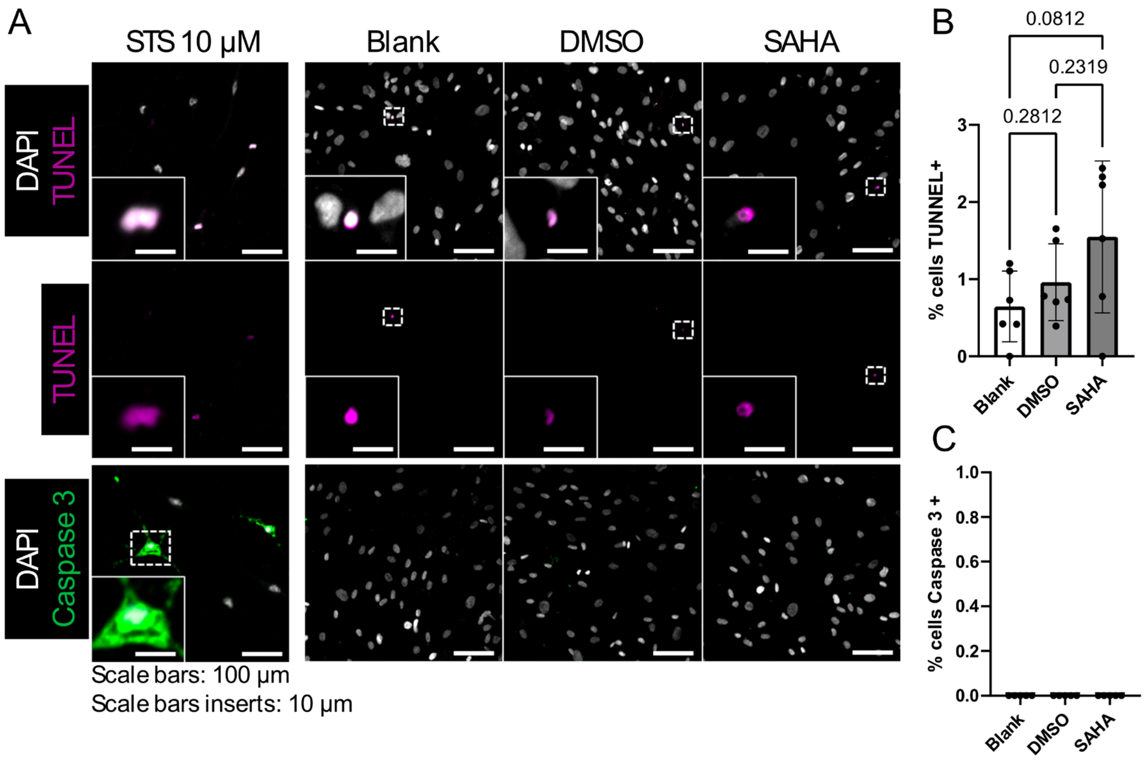

Supplementary figure 1 : SAHA treatment of hSSC in vitro does not induce cell death.
(A) Representative images and quantification of (B) TUNEL staining (purple) and (C) cleaved caspase 3 immunostaining (green) in the WIOTM after 7 days of culture under three conditions: osteogenic media alone (Blank), osteogenic media + DMSO, and osteogenic media + SAHA (1 µM). Staurosporine 10 µM was added 6h before the end of the experiment and used as a positive control of cell death. The data represents the mean ± s.d. Statistical analysis: ANOVA followed by unpaired t test with Welch's correction. N=6 WIOTM, n ≥ 97 cells per WIOTM.

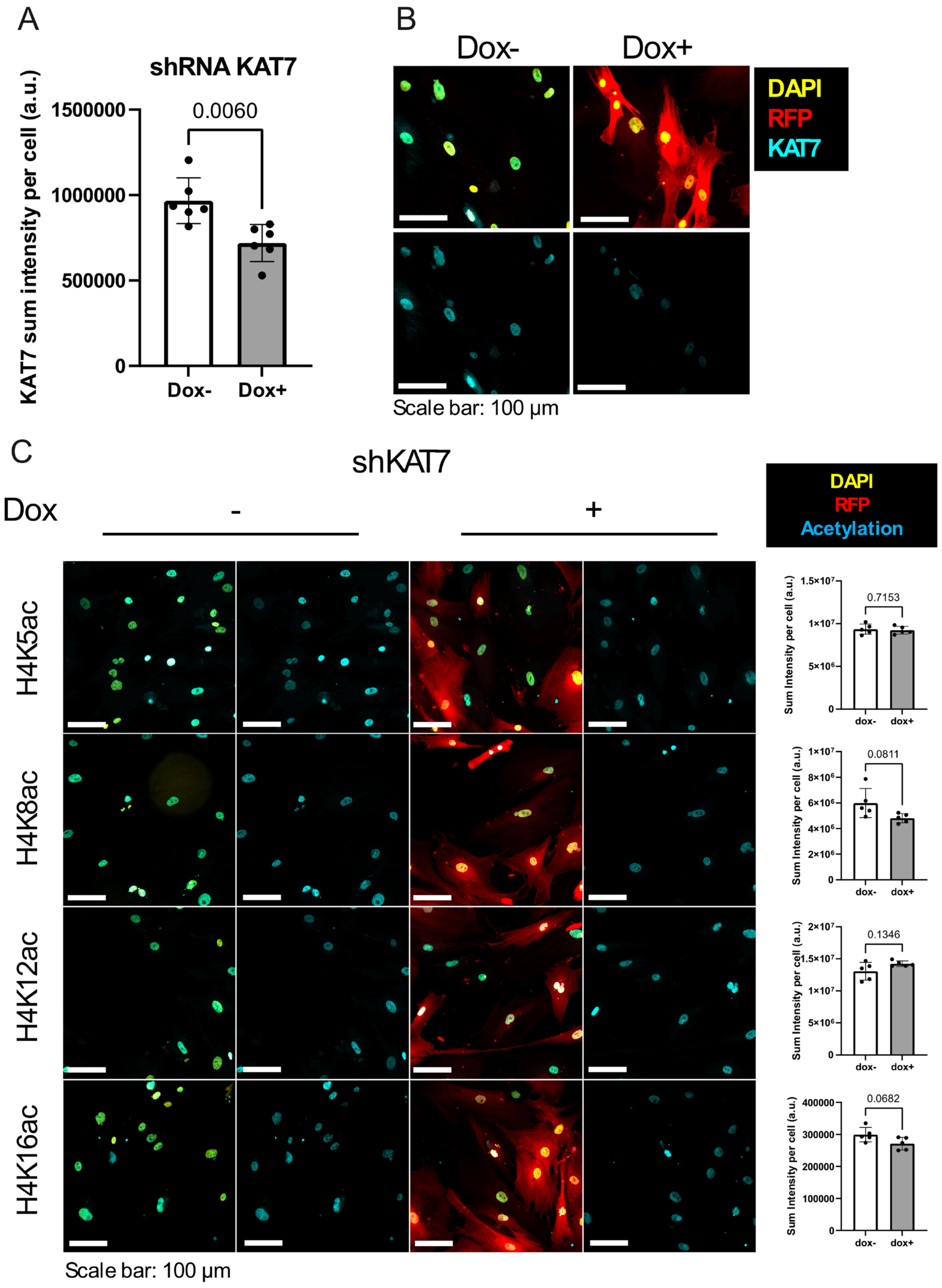
A
shRNA KAT7
KAT7 sum intensity per cell (a.u.)
1500000
1000000
500000
0
0.0060
Dox-
Dox+
B
Dox-
Dox+
DAPI
RFP
KAT7
Scale bar: 100 µm
C
shKAT7
Dox
-
+
DAPI
RFP
Acetylation
H4K5ac
H4K8ac
H4K12ac
H4K16ac
Sum Intensity per cell (a.u.)
0.7153
0.0811
0.1346
0.0682
dox-
dox+
Scale bar: 100 µm

Supplementary figure 2: Kat7 knock-out does not affect H4 acetylation.

(A) Quantification and (B) representative images of Kat7 (cyan) immunostaining in hSSCs after transduction of a doxycycline inducible shRNA targeting Kat7 and 7 days of induction. Induction of the shRNA with doxycycline treatment also induces the expression of the red fluorescent protein (RFP) shown in red. The data represents the mean ± s.d. Statistical analysis: Uncorrected Fisher's LSD tests. N=6 biological replicates, n ≥ 21 cells. (C) Representative images and quantification of the expression of a panel of H4 acetylation in hSSCs transduced with shRNA targeting *Kat7* after 7 days of induction. Acetylation marks are shown in blue, RFP in red. The data represents the mean ± s.d. Each data point represents a biological replicate. Statistical analysis: Uncorrected Fisher's LSD tests. N=5 biological replicates, n ≥ 19 cells.

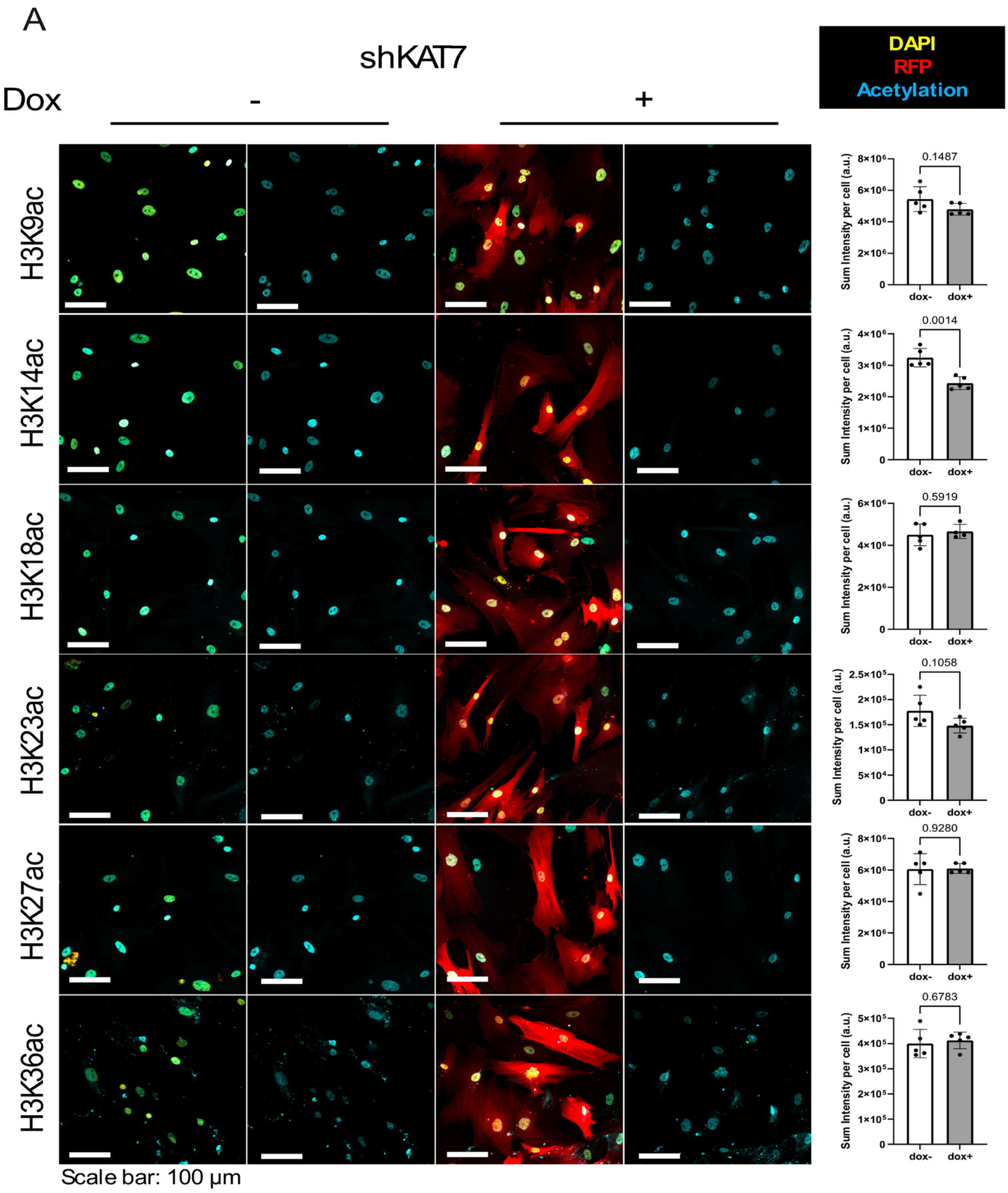

Supplementary figure 3: Kat7 knock-out only affects H3K14ac.

(A) Representative images and quantification of the expression of a panel of H3 acetylation in hSSCs transduced with shRNA targeting *Kat7* after 7 days of induction. Acetylation marks are shown in blue, RFP in red. The data represents the mean ± s.d. Each data point represents a biological replicate. Statistical analysis: Uncorrected Fisher's LSD tests. N=5 biological replicates, n ≥ 23 cells.

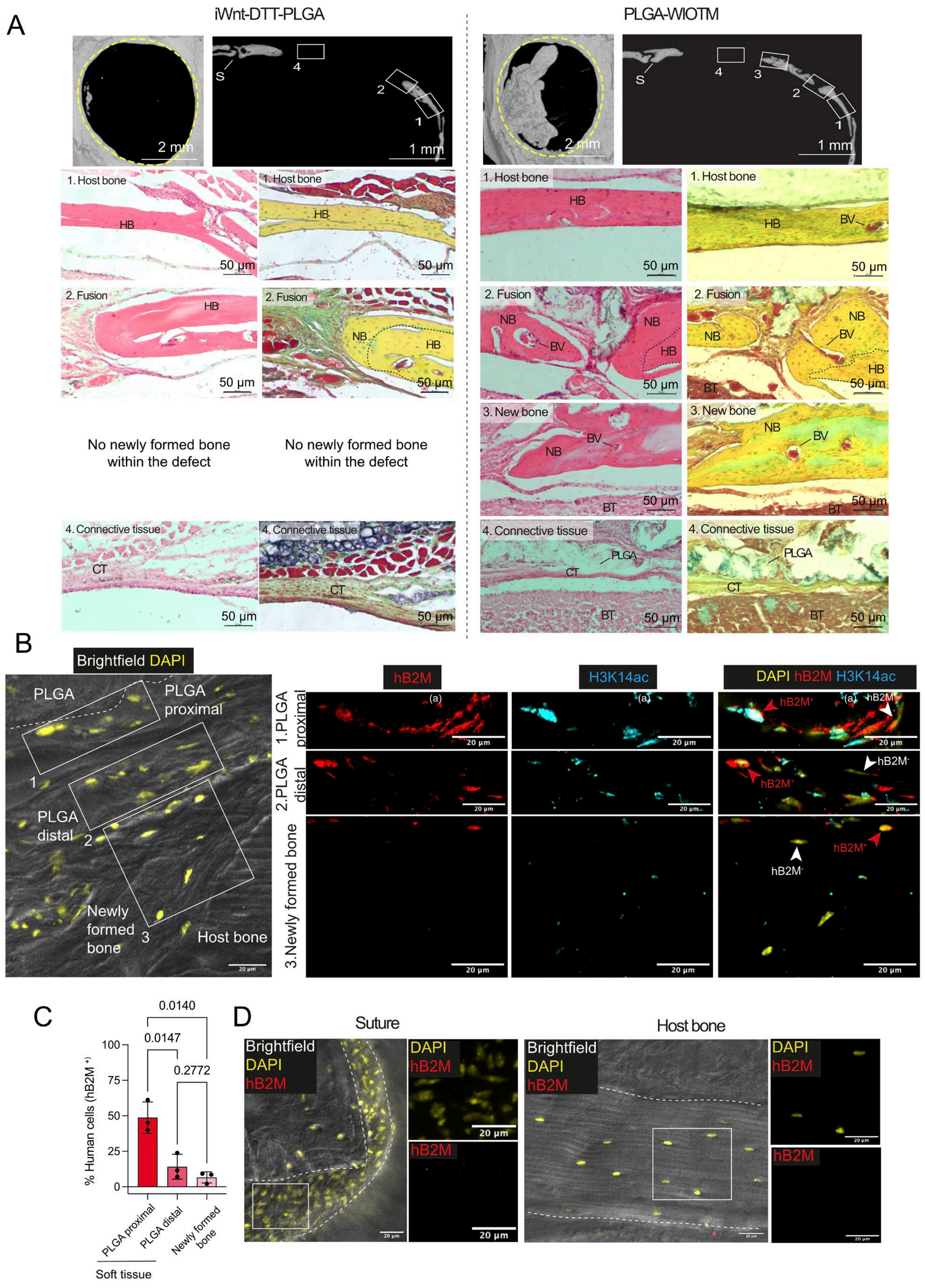
A
iWnt-DTT-PLGA
PLGA-WIOTM
2 mm
1 mm
S
1. Host bone
HB
2. Fusion
NB
3. New bone
4. Connective tissue
CT
BV
BT
PLGA
50 µm
No newly formed bone within the defect
B
Brightfield DAPI
PLGA
PLGA proximal
PLGA distal
Newly formed bone
Host bone
hB2M
H3K14ac
DAPI hB2M H3K14ac
1.PLGA proximal
2.PLGA distal
3.Newly formed bone
20 µm
C
% Human cells (hB2M+)
0.0140
0.0147
0.2772
PLGA proximal
PLGA distal
Newly formed bone
Soft tissue
D
Suture
Host bone
Brightfield
DAPI
hB2M

Supplementary figure 4: Histological characterization of the bone defects.
(A) Representative µCT images with top view and frontal digital sections of defects covered by iWnt-DTT-PLGA or PLGA-WIOTM. The dashed yellow circle indicates the edges of the drilled defects. S: sagittal suture of the calvaria. The numbered squares (1-4) indicate the enlarged area where representative images of H&E and Movat's pentachrome staining have been taken. HB: host bone, BV: blood vessel, NB: newly formed bone, HB: host bone, BT: brain tissue, CT: connective tissue. The dashed black line indicates the limit of the host bone (i.e., the edge of the drilled defect). (B) Representative images of the newly formed bone overlaid by the soft tissue, and the PLGA bandage. The dashed line indicates the limit of the PLGA. The numbered (1-3) white squares indicate the enlarged area on the right panel. The human marker (human beta-2 microglobulin (hB2M)) is displayed in red and indicates cells of human origin, based on tissue-specific threshold. The red and white arrowheads indicate positive ($hB2M^+$) and negative ($hB2M^-$) cells, respectively. H3K14ac is displayed in blue with the same threshold in all images. (C) Proportion of human cells ($hB2M^+$) in each tissue class. Data are presented as mean ± S.D. Statistical analyses: uncorrected ANOVA test. N=3 animals, n = 194 cells. (D) Representative images of immunostaining of host tissue (suture and host bone) where no human cells (labelled by hB2M) were found. The dashed lne indicates the limit of the sagittal suture (left panel), or the host bone (right panel).

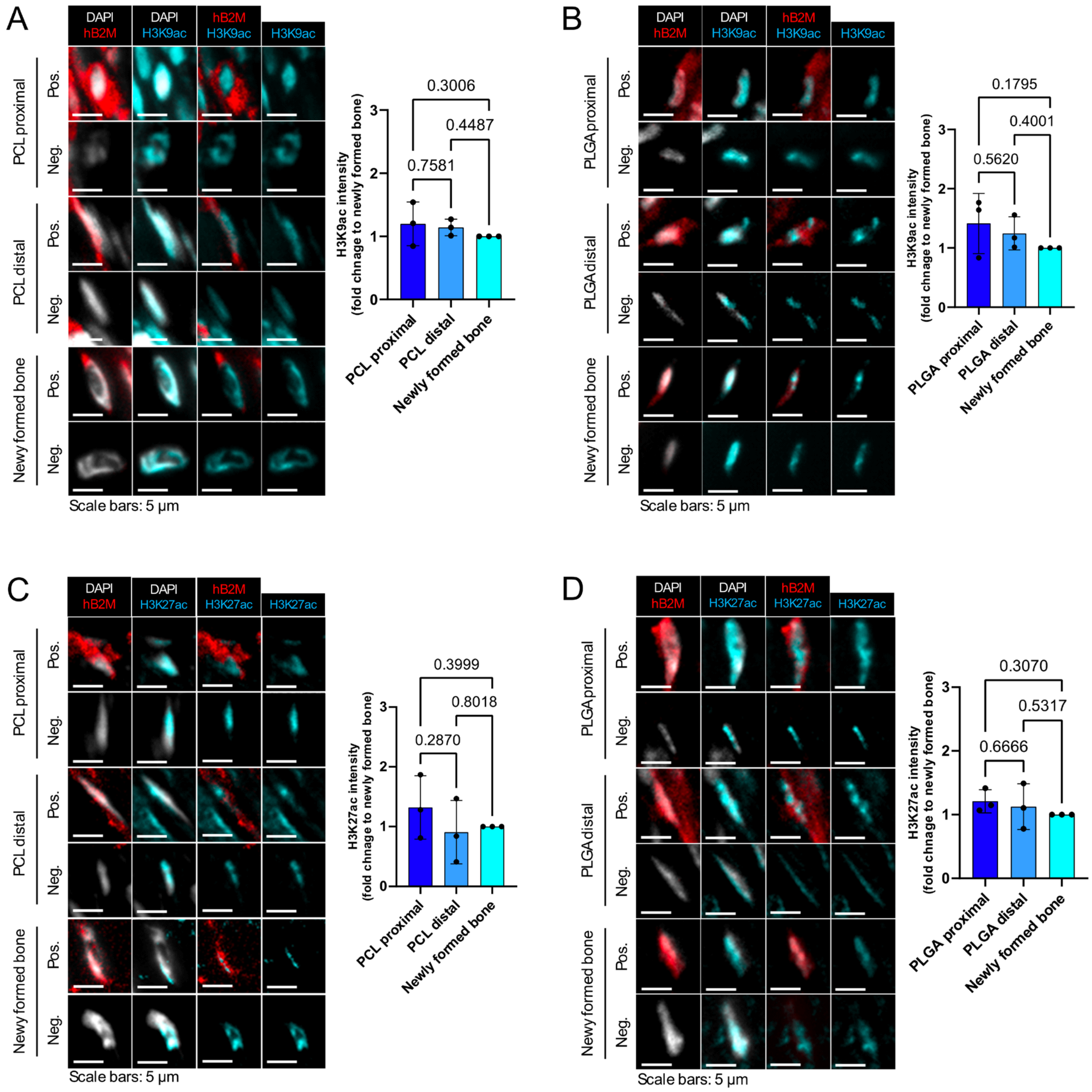

Supplementary figure 5: Localized Wnt-3a signaling on biodegradable polymers does not polarize H3K9ac and H3K27ac in transplanted hSSCs in a critical size defect model in mice calvaria.

(A) Representative images and quantification of tissue sections immunostained with H3K9ac and human beta-2 microglobulin. PCL proximal (i.e., close to the PCL), PCL distal (i.e., at distance from the PCL, close to the newly formed bone); newly formed bone (i.e., within the newly formed bone tissue). The human marker (human beta-2 microglobulin) is displayed in red and indicates cells of human origin, based on tissue-specific threshold (native bone). H3K9ac is displayed in blue with the same threshold in all images. H3K9ac intensity is quantified in cells of human origin in each tissue class. N = 3 animals, n = 149 cells. (B) Similar to (A) but here the calvarial defect was covered with a PLGA WIOTM bandage. N = 3 animals, n = 159 cells. (C) Similar to (A) but sections were immunostained with with H3K27ac (blue) and human beta-2 microglobulin (Red). N = 3 animals, n = 130 cells. (D) Similar to (C) but here the calvarial defect was covered with a PLGA WIOTM bandage. N = 3 animals, n = 137 cells. All data are presented as mean ± S.D. Statistical analyses: ANOVA followed by uncorrected Fisher's LSD.

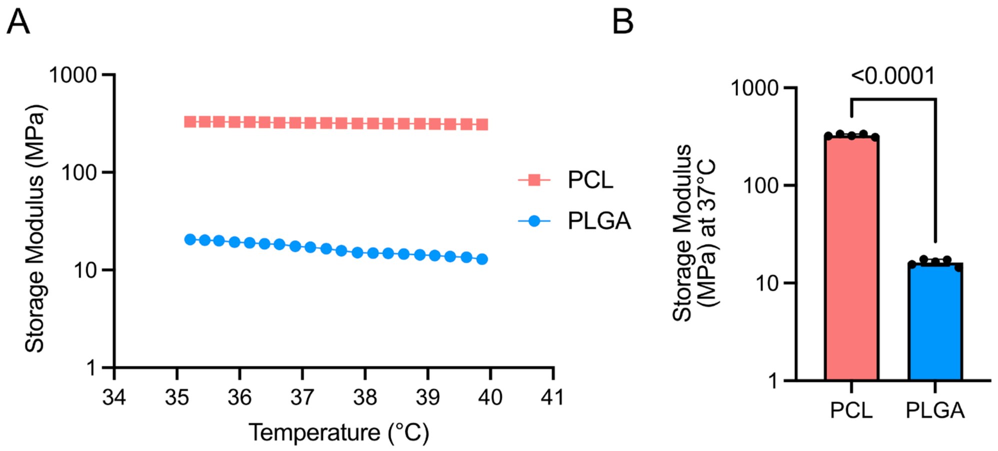

Supplementary figure 6: Mechanical properties of PCL and PLGA films
(A) Temperature ramp and measure of the storage moduli of polycaprolactone (PCL) and poly(lactic-co-glycolic acid) (PLGA) films (1 Hz and 0.5% strain) within a temperature range of 35-40 °C.(B) Storage moduli at 37 °C for PCL and PLGA films. Data are presented as mean ± S.D. N=5 films per group. Statistical analysis: unpaired Welch's t-test.

Fig 3C-D	DAPI	H3K14ac	H3K9ac	RNApolS2P	OPN:CDH13
DMSO	<0.0001	<0.0001	0.0022	<0.0001	0.0061
SAHA	0.0083	0.3802	0.9194	0.008	0.4987

Supplementary table 1: p-values after one sample t-test from figure 3C and D. Camparison to the theorical value of 1

Fig 4B & D	H3K14ac		OPN:CDH13	
	shScramble	shKAT7	shScramble	shKAT7
dox-	<0.0001	0.0019	0.0032	0.0143
dox+	0.0257	0.9712	0.0017	0.0206

Supplementary table 2: p-values after one sample t-test from figure 4B and D. Camparison to the theorical value of 1

Supplementary Table 3: list of genes corresponding to significantly decreased and increases peaks identified by ATAC sequencing.

Decreased Peaks		Increased Peaks	
Gene Symbol corresponding to decreased peak	Gene Decription	Gene Symbol correponding to increased peak	Gene Description
A4GALT	alpha 1,4-galactosyltransferase (P blood group)	ABCA13	ATP binding cassette subfamily A member 13
ABCG1	ATP binding cassette subfamily G member 1	ABCB8	ATP binding cassette subfamily B member 8
ABL1	ABL proto-oncogene 1, non-receptor tyrosine kinase	ABCC4	ATP binding cassette subfamily C member 4
ACCSL	1-aminocyclopropane-1-carboxylate synthase homolog (inactive) like	ABHD5	abhydrolase domain containing 5, lysophosphatidic acid acyltransferase
ACO2	aconitase 2	ACTBL2	actin beta like 2
ACOT12	acyl-CoA thioesterase 12	ACTL7B	actin like 7B
ACSL4	acyl-CoA synthetase long chain family member 4	ACTN4	actinin alpha 4
ACTN1	actinin alpha 1	ACTR3BP2	ACTR3B pseudogene 2
ACTR3BP2	ACTR3B pseudogene 2	ACTR3BP5	ACTR3B pseudogene 5
ACVR1	activin A receptor type 1	ADAP1	ArfGAP with dual PH domains 1
ADAM17	ADAM metallopeptidase domain 17	ADCY7	adenylate cyclase 7
ADAM28	ADAM metallopeptidase domain 28	ADRA1B	adrenoceptor alpha 1B
ADAM9	ADAM metallopeptidase domain 9	AFAP1	actin filament associated protein 1
ADAMTS1	ADAM metallopeptidase with thrombospondin type 1 motif 1	AFF1	AF4/FMR2 family member 1
ADAMTS2	ADAM metallopeptidase with thrombospondin type 1 motif 2	ALG10	ALG10 alpha-1,2-glucosyltransferase
ADAMTS3	ADAM metallopeptidase with thrombospondin type 1 motif 3	ALG10B	ALG10 alpha-1,2-glucosyltransferase B
ADGRG6	adhesion G protein-coupled receptor G6	ANKFN1	ankyrin repeat and fibronectin type III domain containing 1
ADH7	alcohol dehydrogenase 7 (class IV), mu or sigma polypeptide	ANKH	ANKH inorganic pyrophosphate transport regulator
ADHFE1	alcohol dehydrogenase iron containing 1	ANKRD20A12P	ankyrin repeat domain 20 family member A12, pseudogene
ADM	adrenomedullin	ANKRD26P1	ankyrin repeat domain 26 pseudogene 1
AFMID	arylformamidase	ANKRD30BL	ankyrin repeat domain 30B like
AFTPH	aftiphilin	ANKRD30BP2	ankyrin repeat domain 30B pseudogene 2
AGAP1	ArfGAP with GTPase domain, ankyrin repeat and PH domain 1	ANKRD31	ankyrin repeat domain 31
AGO2	argonaute RISC catalytic component 2	ANKRD33B	ankyrin repeat domain 33B
AGR3	anterior gradient 3, protein disulphide isomerase family member	ANKRD36B	ankyrin repeat domain 36B
AGTR1	angiotensin II receptor type 1	ANKRD36BP2	ankyrin repeat domain 36B pseudogene 2
AHR	aryl hydrocarbon receptor	ANKRD55	ankyrin repeat domain 55
AKR1C3	aldo-keto reductase family 1 member C3	ANO1	anoctamin 1
ALDH16A1	aldehyde dehydrogenase 16 family member A1	ANP32E	acidic nuclear phosphoprotein 32 family member E
ANHX	anomalous homeobox	ANXA5	annexin A5
ANKS1B	ankyrin repeat and sterile alpha motif domain containing 1B	AP1G1	adaptor related protein complex 1 subunit gamma 1
ANXA1	annexin A1	APCS	amyloid P component, serum
ANXA5	annexin A5	AQP4	aquaporin 4
ARHGAP18	Rho GTPase activating protein 18	AQP7P3	aquaporin 7 pseudogene 3
ARHGAP24	Rho GTPase activating protein 24	ARHGAP27P1	Rho GTPase activating protein 27 pseudogene 1
ARHGEF19	Rho guanine nucleotide exchange factor 19	ARHGEF18	Rho/Rac guanine nucleotide exchange factor 18
ARID5B	AT-rich interaction domain 5B	ARHGEF28	Rho guanine nucleotide exchange factor 28
ARRDC3-AS1	ARRDC3 antisense RNA 1	ARHGEF37	Rho guanine nucleotide exchange factor 37
ASB9P1	ankyrin repeat and SOCS box containing 9 pseudogene 1	ARID3B	AT-rich interaction domain 3B
ASCC2	activating signal cointegrator 1 complex subunit 2	ARID5B	AT-rich interaction domain 5B
ASIP	agouti signaling protein	ARL8B	ADP ribosylation factor like GTPase 8B
ATF3	activating transcription factor 3	ARMC9	armadillo repeat containing 9
ATP2B1-AS1	ATP2B1 antisense RNA 1	ARNT2	aryl hydrocarbon receptor nuclear translocator 2
ATP5IF1	ATP synthase inhibitory factor subunit 1	ATL2	atlastin GTPase 2
ATP6AP1L	ATPase H+ transporting accessory protein 1 like	ATP6AP1L	ATPase H+ transporting accessory protein 1 like
ATXN1	ataxin 1	ATP6V0A1	ATPase H+ transporting V0 subunit a1
AZI2	5-azacytidine induced 2	B4GALNT3	beta-1,4-N-acetyl-galactosaminyltransferase 3
B4GALT6	beta-1,4-galactosyltransferase 6	BAGE	B melanoma antigen
BCDIN3D	BCDIN3 domain containing RNA methyltransferase	BAGE3	BAGE family member 3
BCL2L10	BCL2 like 10	BATF	basic leucine zipper ATF-like transcription factor
BCL6	BCL6 transcription repressor	BCAR3	BCAR3 adaptor protein, NSP family member
BMPER	BMP binding endothelial regulator	BCL11B	BAF chromatin remodeling complex subunit BCL11B
BRCA2	BRCA2 DNA repair associated	BCL2L1	BCL2 like 1
BTC	betacellulin	BCRP2	BCR pseudogene 2
C16orf95	chromosome 16 open reading frame 95	BDH1	3-hydroxybutyrate dehydrogenase 1
C17orf50	chromosome 17 open reading frame 50	BMP6	bone morphogenetic protein 6
C1S	complement C1s	BMS1P18	BMS1 pseudogene 18
CABLES1	Cdk5 and Abl enzyme substrate 1	BNIP3L	BCL2 interacting protein 3 like
CACNA1A	calcium voltage-gated channel subunit alpha1 A	BRCA2	BRCA2 DNA repair associated
CARD19	caspase recruitment domain family member 19	BRD3OS	BRD3 opposite strand
CARS2	cysteinyl-tRNA synthetase 2, mitochondrial	BTBD11	BTB domain containing 11
CAV1	caveolin 1	BTF3	basic transcription factor 3
CBLC	Cbl proto-oncogene C	C1orf198	chromosome 1 open reading frame 198
CCDC107	coiled-coil domain containing 107	C1QTNF3	C1q and TNF related 3
CCDC115	coiled-coil domain containing 115	C1QTNF7	C1q and TNF related 7
CCDC144NL	CCDC144A N-terminal pseudogene	C2CD3	C2 domain containing 3 centriole elongation regulator
CCDC177	coiled-coil domain containing 177	C2orf73	chromosome 2 open reading frame 73
CCDC200	coiled-coil domain containing 200	C4orf51	chromosome 4 open reading frame 51
CCDC57	coiled-coil domain containing 57	C5orf24	chromosome 5 open reading frame 24
CCL20	C-C motif chemokine ligand 20	C5orf46	chromosome 5 open reading frame 46
CCN2	cellular communication network factor 2	C7orf61	chromosome 7 open reading frame 61
CCN6	cellular communication network factor 6	C8orf37	chromosome 8 open reading frame 37
CCNI2	cyclin I family member 2	CA12	carbonic anhydrase 12
CCNL1	cyclin L1	CALM2	calmodulin 2
CCR7	C-C motif chemokine receptor 7	CALN1	calneuron 1
CD27	CD27 molecule	CARTPT	CART prepropeptide
CD28	CD28 molecule	CBLB	Cbl proto-oncogene B
CD2AP	CD2 associated protein	CCDC80	coiled-coil domain containing 80
CD82	CD82 molecule	CCL2	C-C motif chemokine ligand 2
CDC14B	cell division cycle 14B	CCN1	cellular communication network factor 1
CDC42EP3	CDC42 effector protein 3	CCT6P3	chaperonin containing TCP1 subunit 6 pseudogene 3
CDKN2B-AS1	CDKN2B antisense RNA 1	CCT8L2	chaperonin containing TCP1 subunit 8 like 2
CEP95	centrosomal protein 95	CD28	CD28 molecule
CILK1	ciliogenesis associated kinase 1	CD44	CD44 molecule (Indian blood group)

CKAP2L	cytoskeleton associated protein 2 like	CDCA7L	cell division cycle associated 7 like
CLCN3	chloride voltage-gated channel 3	CDH11	cadherin 11
CLEC16A	C-type lectin domain containing 16A	CDH2	cadherin 2
COL12A1	collagen type XII alpha 1 chain	CDH5	cadherin 5
COL1A2	collagen type I alpha 2 chain	CDIN1	CDAN1 interacting nuclease 1
COL28A1	collagen type XXVIII alpha 1 chain	CDK15	cyclin dependent kinase 15
COL5A1	collagen type V alpha 1 chain	CDKN2B-AS1	CDKN2B antisense RNA 1
COPS8	COP9 signalosome subunit 8	CEBPD	CCAAT enhancer binding protein delta
COQ2	coenzyme Q2, polyprenyltransferase	CFAP20DC	CFAP20 domain containing
COX18	cytochrome c oxidase assembly factor COX18	CHEK2P2	checkpoint kinase 2 pseudogene 2
CP	ceruloplasmin	CLASP1	cytoplasmic linker associated protein 1
CPAMD8	C3 and PZP like alpha-2-macroglobulin domain containing 8	CLDN23	claudin 23
CPEB4	cytoplasmic polyadenylation element binding protein 4	CLSTN2	calsyntenin 2
CPED1	cadherin like and PC-esterase domain containing 1	CLTCL1	clathrin heavy chain like 1
CPSF6	cleavage and polyadenylation specific factor 6	CMTM7	CKLF like MARVEL transmembrane domain containing 7
CRIP1	cysteine rich protein 1	COL1A1	collagen type I alpha 1 chain
CROCC	ciliary rootlet coiled-coil, rootletin	COL4A2	collagen type IV alpha 2 chain
CROCCP3	CROCC pseudogene 3	COL6A2	collagen type VI alpha 2 chain
CTAGE1	cutaneous T cell lymphoma-associated antigen 1	COL8A1	collagen type VIII alpha 1 chain
CTSB	cathepsin B	COLGALT2	collagen beta(1-O)galactosyltransferase 2
CUBN	cubilin	CORIN	corin, serine peptidase
CWC27	CWC27 spliceosome associated cyclophilin	CPEB4	cytoplasmic polyadenylation element binding protein 4
CYP1B1-AS1	CYP1B1 antisense RNA 1	CPLANE2	ciliogenesis and planar polarity effector 2
CYTH3	cytohesin 3	CRCP	CGRP receptor component
CYTIP	cytohesin 1 interacting protein	CRIM1	cysteine rich transmembrane BMP regulator 1
DAB1	DAB adaptor protein 1	CRLF3	cytokine receptor like factor 3
DACT1	dishevelled binding antagonist of beta catenin 1	CTPS1	CTP synthase 1
DCLRE1C	DNA cross-link repair 1C	CUBN	cubilin
DDI2	DNA damage inducible 1 homolog 2	CUEDC1	CUE domain containing 1
DDIT4	DNA damage inducible transcript 4	CWH43	cell wall biogenesis 43 C-terminal homolog
DEPDC1B	DEP domain containing 1B	CYP2U1	cytochrome P450 family 2 subfamily U member 1
DOCK10	dedicator of cytokinesis 10	CYTOR	cytoskeleton regulator RNA
DUSP1	dual specificity phosphatase 1	DAAM1	dishevelled associated activator of morphogenesis 1
DUSP5P1	dual specificity phosphatase 5 pseudogene 1	DARS1	aspartyl-tRNA synthetase 1
DUT	deoxyuridine triphosphatase	DCAF1	DDB1 and CUL4 associated factor 1
DUX4L3	double homeobox 4 like 3 (pseudogene)	DCC	DCC netrin 1 receptor
DZIP1L	DAZ interacting zinc finger protein 1 like	DDHD1	DDHD domain containing 1
EBLN2	endogenous Bornavirus like nucleoprotein 2	DEFB115	defensin beta 115
EDEM3	ER degradation enhancing alpha-mannosidase like protein 3	DEUP1	deuterosome assembly protein 1
EDIL3	EGF like repeats and discoidin domains 3	DGCR6	DiGeorge syndrome critical region gene 6
EEPD1	endonuclease/exonuclease/phosphatase family domain containing 1	DHCR24	24-dehydrocholesterol reductase
EFEMP1	EGF containing fibulin extracellular matrix protein 1	DIO2-AS1	DIO2 antisense RNA 1
EFNA3	ephrin A3	DLEU7-AS1	DLEU7 antisense RNA 1
EGR1	early growth response 1	DLG1	discs large MAGUK scaffold protein 1
EIF1	eukaryotic translation initiation factor 1	DLG5	discs large MAGUK scaffold protein 5
ELAPOR1	endosome-lysosome associated apoptosis and autophagy regulator 1	DLX6-AS1	DLX6 antisense RNA 1
EMILIN2	elastin microfibril interfacer 2	DNAH8	dynein axonemal heavy chain 8
ENAH	ENAH actin regulator	DNAJB1	DnaJ heat shock protein family (Hsp40) member B1
ENSA	endosulfine alpha	DNAL1	dynein axonemal light chain 1
EOMES	eomesodermin	DOCK2	dedicator of cytokinesis 2
EPAS1	endothelial PAS domain protein 1	DOCK5	dedicator of cytokinesis 5
EPHA2	EPH receptor A2	DPYSL3	dihydropyrimidinase like 3
EPHA4	EPH receptor A4	DSTYK	dual serine/threonine and tyrosine protein kinase
EPHA6	EPH receptor A6	DUSP10	dual specificity phosphatase 10
EPS8	epidermal growth factor receptor pathway substrate 8	DUSP23	dual specificity phosphatase 23
ERCC1	ERCC excision repair 1, endonuclease non-catalytic subunit	E2F7	E2F transcription factor 7
ESRRG	estrogen related receptor gamma	EDN1	endothelin 1
ETS2	ETS proto-oncogene 2, transcription factor	EGFR	epidermal growth factor receptor
FAM111A	FAM111 trypsin like peptidase A	EIF2AK1	eukaryotic translation initiation factor 2 alpha kinase 1
FAM114A1	family with sequence similarity 114 member A1	EIF4H	eukaryotic translation initiation factor 4H
FAM13A	family with sequence similarity 13 member A	ELAPOR1	endosome-lysosome associated apoptosis and autophagy regulator 1
FAM174A	family with sequence similarity 174 member A	ELOA3DP	elongin A3 family member D, pseudogene
FAM207A	family with sequence similarity 207 member A	ELOA3P	elongin A3, pseudogene
FAM20A	FAM20A golgi associated secretory pathway pseudokinase	EMB	embigin
FAM27B	family with sequence similarity 27 member B	EMBP1	embigin pseudogene 1
FAM72A	family with sequence similarity 72 member A	EPHA3	EPH receptor A3
FAM72D	family with sequence similarity 72 member D	ESM1	endothelial cell specific molecule 1
FAM86C1P	family with sequence similarity 86 member C1, pseudogene	ETS1	ETS proto-oncogene 1, transcription factor
FAM86DP	family with sequence similarity 86 member D, pseudogene	ETV6	ETS variant transcription factor 6
FAR2	fatty acyl-CoA reductase 2	EXT1	exostosin glycosyltransferase 1
FAS-AS1	FAS antisense RNA 1	EYA3	EYA transcriptional coactivator and phosphatase 3
FAXDC2	fatty acid hydroxylase domain containing 2	F2RL1	F2R like trypsin receptor 1
FBXL20	F-box and leucine rich repeat protein 20	FAHD2CP	fumarylacetoacetate hydrolase domain containing 2C, pseudogene
FBXL7	F-box and leucine rich repeat protein 7	FAM138B	family with sequence similarity 138 member B
FBXO31	F-box protein 31	FAM172A	family with sequence similarity 172 member A
FBXW5	F-box and WD repeat domain containing 5	FAM182B	family with sequence similarity 182 member B
FBXW7	F-box and WD repeat domain containing 7	FAM20C	FAM20C golgi associated secretory pathway kinase
FCGR2A	Fc fragment of IgG receptor IIa	FAM227A	family with sequence similarity 227 member A
FER1L6	fer-1 like family member 6	FAM241A	family with sequence similarity 241 member A
FERMT1	FERM domain containing kindlin 1	FAM50B	family with sequence similarity 50 member B
FEZ2	fasciculation and elongation protein zeta 2	FAM71D	family with sequence similarity 71 member D
FGF7P6	fibroblast growth factor 7 pseudogene 6	FANK1	fibronectin type III and ankyrin repeat domains 1
FILIP1	filamin A interacting protein 1	FBXO32	F-box protein 32
FILNC1	FOXO induced long non-coding RNA 1	FEZ2	fasciculation and elongation protein zeta 2
FLJ42969	uncharacterized LOC441374	FHL2	four and a half LIM domains 2
FOS	Fos proto-oncogene, AP-1 transcription factor subunit	FIGNL1	fidgetin like 1
FOSL1	FOS like 1, AP-1 transcription factor subunit	FILNC1	FOXO induced long non-coding RNA 1
FOSL2	FOS like 2, AP-1 transcription factor subunit	FNBP1	formin binding protein 1
FTCD	formimidoyltransferase cyclodeaminase	FNIP2	folliculin interacting protein 2

FYCO1	FYVE and coiled-coil domain autophagy adaptor 1	FOXC1	forkhead box C1
FZD1	frizzled class receptor 1	FOXD4L5	forkhead box D4 like 5
GAB2	GRB2 associated binding protein 2	FOXP1	forkhead box P1
GADD45A	growth arrest and DNA damage inducible alpha	FRG1	FSHD region gene 1
GADL1	glutamate decarboxylase like 1	FRG1BP	FSHD region gene 1 family member B, pseudogene
GALNT15	polypeptide N-acetylgalactosaminyltransferase 15	FRG1HP	FSHD region gene 1 family member H, pseudogene
GALNT5	polypeptide N-acetylgalactosaminyltransferase 5	FRG1JP	FSHD region gene 1 family member J, pseudogene
GDNF	glial cell derived neurotrophic factor	FRMD5	FERM domain containing 5
GK5	glycerol kinase 5	FRMD6	FERM domain containing 6
GLIDR	glioblastoma down-regulated RNA	FRY	FRY microtubule binding protein
GLTPD2	glycolipid transfer protein domain containing 2	FSIP1	fibrous sheath interacting protein 1
GMEB1	glucocorticoid modulatory element binding protein 1	FUT10	fucosyltransferase 10
GOLGA8CP	golgin A8 family member C, pseudogene	GAD1	glutamate decarboxylase 1
GPR108	G protein-coupled receptor 108	GADD45B	growth arrest and DNA damage inducible beta
GRAP	GRB2 related adaptor protein	GADL1	glutamate decarboxylase like 1
GRAPL	GRB2 related adaptor protein like	GAREM2	GRB2 associated regulator of MAPK1 subtype 2
GRHL2	grainyhead like transcription factor 2	GART	phosphoribosylglycinamide formyltransferase, phosphoribosylglycinamide synthetase, phosphoribosylaminoimidazole synthetase
GRHL3	grainyhead like transcription factor 3	GCLC	glutamate-cysteine ligase catalytic subunit
GSAP	gamma-secretase activating protein	GCM1	glial cells missing transcription factor 1
GSK3A	glycogen synthase kinase 3 alpha	GGT3P	gamma-glutamyltransferase 3 pseudogene
GTF2I	general transcription factor IIi	GGT8P	gamma-glutamyltransferase 8 pseudogene
GTF3C4	general transcription factor IIIC subunit 4	GJA10	gap junction protein alpha 10
GXYLT2	glucoside xylosyltransferase 2	GJD2	gap junction protein delta 2
GYG2P1	glycogenin 2 pseudogene 1	GLI2	GLI family zinc finger 2
GYPC	glycophorin C (Gerbich blood group)	GLIS3	GLIS family zinc finger 3
HDAC9	histone deacetylase 9	GMDS-DT	GMDS divergent transcript
HEBP2	heme binding protein 2	GPAT3	glycerol-3-phosphate acyltransferase 3
HEG1	heart development protein with EGF like domains 1	GPR161	G protein-coupled receptor 161
HFM1	helicase for meiosis 1	GRB14	growth factor receptor bound protein 14
HINT1	histidine triad nucleotide binding protein 1	GS1-124K5.11	RAB guanine nucleotide exchange factor 1 pseudogene
HMGN4	high mobility group nucleosomal binding domain 4	GUSBP2	GUSB pseudogene 2
HNMT	histamine N-methyltransferase	GUSBP4	GUSB pseudogene 4
HNRNPA3P1	heterogeneous nuclear ribonucleoprotein A3 pseudogene 1	GYG2P1	glycogenin 2 pseudogene 1
HS1BP3	HCLS1 binding protein 3	H1-9P	H1.9 linker histone, pseudogene
HTR7	5-hydroxytryptamine receptor 7	HADH	hydroxyacyl-CoA dehydrogenase
IBTK	inhibitor of Bruton tyrosine kinase	HAS2	hyaluronan synthase 2
ID1	inhibitor of DNA binding 1, HLH protein	HAVCR1P1	hepatitis A virus cellular receptor 1 pseudogene 1
ID3	inhibitor of DNA binding 3, HLH protein	HCN1	hyperpolarization activated cyclic nucleotide gated potassium channel 1
IFFO2	intermediate filament family orphan 2	HEXIM2	HEXIM P-TEFb complex subunit 2
IGF2BP2	insulin like growth factor 2 mRNA binding protein 2	HHIP	hedgehog interacting protein
IGF2BP2-AS1	IGF2BP2 antisense RNA 1	HIC1	HIC ZBTB transcriptional repressor 1
IGF2R	insulin like growth factor 2 receptor	HIVEP3	HIVEP zinc finger 3
IGFBP3	insulin like growth factor binding protein 3	HMCN1	hemicentin 1
IKZF2	IKAROS family zinc finger 2	HORMAD2	HORMA domain containing 2
IL15RA	interleukin 15 receptor subunit alpha	HOXB1	homeobox B1
IL1R2	interleukin 1 receptor type 2	HS3ST3A1	heparan sulfate-glucosamine 3-sulfotransferase 3A1
IL21	interleukin 21	HSF2BP	heat shock transcription factor 2 binding protein
IL9R	interleukin 9 receptor	HSPA4	heat shock protein family A (Hsp70) member 4
IP6K2	inositol hexakisphosphate kinase 2	ID3	inhibitor of DNA binding 3, HLH protein
IRAK2	interleukin 1 receptor associated kinase 2	IFT80	intraflagellar transport 80
IREB2	iron responsive element binding protein 2	IGF1R	insulin like growth factor 1 receptor
IRS1	insulin receptor substrate 1	IGFBP3	insulin like growth factor binding protein 3
ITGA1	integrin subunit alpha 1	IGFBP7-AS1	IGFBP7 antisense RNA 1
ITGB1	integrin subunit beta 1	IL1R1	interleukin 1 receptor type 1
ITPRIPL1	ITPRIP like 1	IL31RA	interleukin 31 receptor A
ITSN1	intersectin 1	IMPA2	inositol monophosphatase 2
IWS1	interacts with SUPT6H, CTD assembly factor 1	IMPG2	interphotoreceptor matrix proteoglycan 2
JUN	Jun proto-oncogene, AP-1 transcription factor subunit	INTU	inturned planar cell polarity protein
KCNA5	potassium voltage-gated channel subfamily A member 5	IRF4	interferon regulatory factor 4
KCNIP4	potassium voltage-gated channel interacting protein 4	IRX3	iroquois homeobox 3
KCNK2	potassium two pore domain channel subfamily K member 2	ITGAV	integrin subunit alpha V
KDM6B	lysine demethylase 6B	ITPK1-AS1	ITPK1 antisense RNA 1
KIAA2013	KIAA2013	JARID2	jumonji and AT-rich interaction domain containing 2
KIF16B	kinesin family member 16B	JDP2	Jun dimerization protein 2
KIF24	kinesin family member 24	KCNJ2	potassium inwardly rectifying channel subfamily J member 2
KIF5C	kinesin family member 5C	KCNJ6	potassium inwardly rectifying channel subfamily J member 6
KLF4	Kruppel like factor 4	KCNJ9	potassium inwardly rectifying channel subfamily J member 9
KLF6	Kruppel like factor 6	KCNMA1	potassium calcium-activated channel subfamily M alpha 1
KLHL30	kelch like family member 30	KCNS3	potassium voltage-gated channel modifier subfamily S member 3
KRAS	KRAS proto-oncogene, GTPase	KCNU1	potassium calcium-activated channel subfamily U member 1
KYNU	kynureninase	KCTD16	potassium channel tetramerization domain containing 16
L3MBTL3	L3MBTL histone methyl-lysine binding protein 3	KHDRBS2	KH RNA binding domain containing, signal transduction associated 2
LAMA4	laminin subunit alpha 4	KIT	KIT proto-oncogene, receptor tyrosine kinase
LINC00111	long intergenic non-protein coding RNA 111	KLF6	Kruppel like factor 6
LINC00114	long intergenic non-protein coding RNA 114	KMT2C	lysine methyltransferase 2C
LINC00293	long intergenic non-protein coding RNA 293	KRT19P2	keratin 19 pseudogene 2
LINC00324	long intergenic non-protein coding RNA 324	KRT34	keratin 34
LINC00332	long intergenic non-protein coding RNA 332	KRT39	keratin 39
LINC00347	long intergenic non-protein coding RNA 347	KRT7	keratin 7
LINC00474	long intergenic non-protein coding RNA 474	KRT81	keratin 81
LINC00583	long intergenic non-protein coding RNA 583	KRTAP2-3	keratin associated protein 2-3
LINC00656	long intergenic non-protein coding RNA 656	KRTAP4-11	keratin associated protein 4-11
LINC00662	long intergenic non-protein coding RNA 662	KTN1-AS1	KTN1 antisense RNA 1
LINC00880	long intergenic non-protein coding RNA 880	LAMC1	laminin subunit gamma 1
LINC00910	long intergenic non-protein coding RNA 910	LARP4B	La ribonucleoprotein 4B
LINC00942	long intergenic non-protein coding RNA 942	LAX1	lymphocyte transmembrane adaptor 1
LINC01085	long intergenic non-protein coding RNA 1085	LCOR	ligand dependent nuclear receptor corepressor
LINC01121	long intergenic non-protein coding RNA 1121	LGALS9B	galectin 9B

LINC01387	long intergenic non-protein coding RNA 1387	LGALS9C	galectin 9C
LINC01554	long intergenic non-protein coding RNA 1554	LIMA1	LIM domain and actin binding 1
LINC01568	long intergenic non-protein coding RNA 1568	LINC00221	long intergenic non-protein coding RNA 221
LINC01588	long intergenic non-protein coding RNA 1588	LINC00273	long intergenic non-protein coding RNA 273
LINC01619	long intergenic non-protein coding RNA 1619	LINC00293	long intergenic non-protein coding RNA 293
LINC02067	long intergenic non-protein coding RNA 2067	LINC00472	long intergenic non-protein coding RNA 472
LINC02363	long intergenic non-protein coding RNA 2363	LINC00565	long intergenic non-protein coding RNA 565
LINC02372	long intergenic non-protein coding RNA 2372	LINC00662	long intergenic non-protein coding RNA 662
LINC02458	long intergenic non-protein coding RNA 2458	LINC00862	long intergenic non-protein coding RNA 862
LINC02591	long intergenic non-protein coding RNA 2591	LINC00911	long intergenic non-protein coding RNA 911
LINC02817	long intergenic non-protein coding RNA 2817	LINC00942	long intergenic non-protein coding RNA 942
LINC02873	long intergenic non-protein coding RNA 2873	LINC00960	long intergenic non-protein coding RNA 960
LOC100128993	uncharacterized LOC100128993	LINC00999	long intergenic non-protein coding RNA 999
LOC100130075	SUZ RNA binding domain containing 1 pseudogene	LINC01010	long intergenic non-protein coding RNA 1010
LOC100506422	putative deoxyuridine 5'-triphosphate nucleotidohydrolase-like protein FLJ16323	LINC01013	long intergenic non-protein coding RNA 1013
LOC284412	uncharacterized LOC284412	LINC01122	long intergenic non-protein coding RNA 1122
LOC646736	uncharacterized LOC646736	LINC01132	long intergenic non-protein coding RNA 1132
LOC650226	ankyrin repeat domain 26 pseudogene	LINC01184	long intergenic non-protein coding RNA 1184
LOC653513	phosphodiesterase 4D interacting protein-like	LINC01592	long intergenic non-protein coding RNA 1592
LPP	LIM domain containing preferred translocation partner in lipoma	LINC01622	long intergenic non-protein coding RNA 1622
LRRC41	leucine rich repeat containing 41	LINC02111	long intergenic non-protein coding RNA 2111
LRRC8B	leucine rich repeat containing 8 VRAC subunit B	LINC02139	long intergenic non-protein coding RNA 2139
LRRFIP1	LRR binding FLII interacting protein 1	LINC02167	long intergenic non-protein coding RNA 2167
LSP1P4	LSP1 pseudogene 4	LINC02346	long intergenic non-protein coding RNA 2346
LSP1P5	LSP1 pseudogene 5	LINC02347	long intergenic non-protein coding RNA 2347
LTBP4	latent transforming growth factor beta binding protein 4	LINC02693	long intergenic non-protein coding RNA 2693
MALAT1	metastasis associated lung adenocarcinoma transcript 1	LINC02873	long intergenic non-protein coding RNA 2873
MAP2K3	mitogen-activated protein kinase kinase 3	LINC02877	long intergenic non-protein coding RNA 2877
MAP2K6	mitogen-activated protein kinase kinase 6	LINC02915	long intergenic non-protein coding RNA 2915
MAP3K20	mitogen-activated protein kinase kinase kinase 20	LIPG	lipase G, endothelial type
MAP4K3-DT	MAP4K3 divergent transcript	LIPH	lipase H
MARCKS	myristoylated alanine rich protein kinase C substrate	LMBR1	limb development membrane protein 1
MATN4	matrilin 4	LOC100133920	methylenetetrahydrofolate dehydrogenase (NADP+ dependent) 1 like pseudogene
MB21D2	Mab-21 domain containing 2	LOC100134317	uncharacterized LOC100134317
MBD6	methyl-CpG binding domain protein 6	LOC100268168	uncharacterized LOC100268168
MBNL1	muscleblind like splicing regulator 1	LOC100506422	putative deoxyuridine 5'-triphosphate nucleotidohydrolase-like protein FLJ16323
MBNL2	muscleblind like splicing regulator 2	LOC286297	methylenetetrahydrofolate dehydrogenase (NADP+ dependent) 1 like pseudogene
MCF2L2	MCF.2 cell line derived transforming sequence-like 2	LOC339862	uncharacterized LOC339862
MCL1	MCL1 apoptosis regulator, BCL2 family member	LOC390705	protein phosphatase 2 regulatory subunit B'', beta pseudogene
MED30	mediator complex subunit 30	LOC441666	zinc finger protein 91 pseudogene
MEF2C	myocyte enhancer factor 2C	LOC644669	ankyrin repeat domain 30B pseudogene
MFGE8	milk fat globule EGF and factor V/VIII domain containing	LOC646813	DExH-box helicase 9 pseudogene
MGAT4C	MGAT4 family member C	LRATD1	LRAT domain containing 1
MGC27382	uncharacterized MGC27382	LRPAP1	LDL receptor related protein associated protein 1
MGC34796	sepiapterin reductase (7,8-dihydrobiopterin:NADP+ oxidoreductase) pseudogene	LRRC42	leucine rich repeat containing 42
MGP	matrix Gla protein	LRRN3	leucine rich repeat neuronal 3
MGST1	microsomal glutathione S-transferase 1	LRTM1	leucine rich repeats and transmembrane domains 1
MIR1208	microRNA 1208	LSP1P4	LSP1 pseudogene 4
MIR1281	microRNA 1281	LSP1P5	LSP1 pseudogene 5
MIR1293	microRNA 1293	LTBP2	latent transforming growth factor beta binding protein 2
MIR1343	microRNA 1343	LTBP4	latent transforming growth factor beta binding protein 4
MIR205HG	MIR205 host gene	LURAP1L	leucine rich adaptor protein 1 like
MIR3124	microRNA 3124	LYPLAL1-DT	LYPLAL1 divergent transcript
MIR3126	microRNA 3126	MACC1	MET transcriptional regulator MACC1
MIR3162	microRNA 3162	MACC1-AS1	MACC1 antisense RNA 1
MIR3170	microRNA 3170	MAFA	MAF bZIP transcription factor A
MIR3605	microRNA 3605	MAGOH	mago homolog, exon junction complex subunit
MIR3675	microRNA 3675	MALAT1	metastasis associated lung adenocarcinoma transcript 1
MIR3692	microRNA 3692	MAMDC2	MAM domain containing 2
MIR3924	microRNA 3924	MANBAL	mannosidase beta like
MIR4266	microRNA 4266	MAP1B	microtubule associated protein 1B
MIR4302	microRNA 4302	MAP3K8	mitogen-activated protein kinase kinase kinase 8
MIR4424	microRNA 4424	MCTP1	multiple C2 and transmembrane domain containing 1
MIR4483	microRNA 4483	METTL4	methyltransferase like 4
MIR4486	microRNA 4486	MGAT5	alpha-1,6-mannosylglycoprotein 6-beta-N-acetylglucosaminyltransferase
MIR4708	microRNA 4708	MIAT	myocardial infarction associated transcript
MIR4729	microRNA 4729	MICAL2	microtubule associated monooxygenase, calponin and LIM domain containing 2
MIR4744	microRNA 4744	MIR1204	microRNA 1204
MIR5191	microRNA 5191	MIR1244-2	microRNA 1244-2
MIR548AR	microRNA 548ar	MIR1252	microRNA 1252
MIR570	microRNA 570	MIR1288	microRNA 1288
MIR585	microRNA 585	MIR2052	microRNA 2052
MIR634	microRNA 634	MIR21	microRNA 21
MIR760	microRNA 760	MIR2278	microRNA 2278
MMD	monocyte to macrophage differentiation associated	MIR3074	microRNA 3074
MMEL1	membrane metalloendopeptidase like 1	MIR3648-1	microRNA 3648-1
MMUT	methylmalonyl-CoA mutase	MIR365B	microRNA 365b
MRPL15	mitochondrial ribosomal protein L15	MIR3675	microRNA 3675
MSC-AS1	MSC antisense RNA 1	MIR3942	microRNA 3942
MTDH	metadherin	MIR4265	microRNA 4265
MTMR7	myotubularin related protein 7	MIR4266	microRNA 4266
MTRNR2L1	MT-RNR2 like 1	MIR4305	microRNA 4305
MTRNR2L2	MT-RNR2 like 2	MIR4422	microRNA 4422
MTRNR2L8	MT-RNR2 like 8	MIR4436A	microRNA 4436a
MUSK	muscle associated receptor tyrosine kinase	MIR4509-1	microRNA 4509-1
MYADM	myeloid associated differentiation marker	MIR4522	microRNA 4522
MYO16-AS1	MYO16 antisense RNA 1	MIR4642	microRNA 4642
MYOZ2	myozenin 2	MIR4674	microRNA 4674
N4BP2	NEDD4 binding protein 2	MIR4677	microRNA 4677

N6AMT1	N-6 adenine-specific DNA methyltransferase 1	MIR4718	microRNA 4718
NAMA	non-protein coding RNA, associated with MAP kinase pathway and growth arrest	MIR5006	microRNA 5006
NAV3	neuron navigator 3	MIR548D2	microRNA 548d-2
NCOA3	nuclear receptor coactivator 3	MIR570	microRNA 570
NDUFS5	NADH:ubiquinone oxidoreductase subunit S5	MIR5702	microRNA 5702
NEAT1	nuclear paraspeckle assembly transcript 1	MIR581	microRNA 581
NEIL3	nei like DNA glycosylase 3	MIR634	microRNA 634
NFE2L1	nuclear factor, erythroid 2 like 1	MIR663AHG	MIR663A host gene
NFE2L2	nuclear factor, erythroid 2 like 2	MIR663B	microRNA 663b
NFIA	nuclear factor I A	MIRLET7A2	microRNA let-7a-2
NFIL3	nuclear factor, interleukin 3 regulated	MIRLET7I	microRNA let-7i
NID1	nidogen 1	MKX	mohawk homeobox
NIM1K	NIM1 serine/threonine protein kinase	MLLT10P1	MLLT10 pseudogene 1
NLK	nemo like kinase	MLPH	melanophilin
NMBR	neuromedin B receptor	MMAA	metabolism of cobalamin associated A
NMNAT2	nicotinamide nucleotide adenylyltransferase 2	MN1	MN1 proto-oncogene, transcriptional regulator
NNMT	nicotinamide N-methyltransferase	MRLN	myoregulin
NNT-AS1	NNT antisense RNA 1	MRPL33	mitochondrial ribosomal protein L33
NOX3	NADPH oxidase 3	MRPL37	mitochondrial ribosomal protein L37
NR2F1-AS1	NR2F1 antisense RNA 1	MSR1	macrophage scavenger receptor 1
NR3C1	nuclear receptor subfamily 3 group C member 1	MTCL1	microtubule crosslinking factor 1
NR5A2	nuclear receptor subfamily 5 group A member 2	MTERF1	mitochondrial transcription termination factor 1
NRG2	neuregulin 2	MTRNR2L1	MT-RNR2 like 1
NRP2	neuropilin 2	MUC17	mucin 17, cell surface associated
NT5E	5'-nucleotidase ecto	MYH9	myosin heavy chain 9
NUFIP1	nuclear FMR1 interacting protein 1	MYL12A	myosin light chain 12A
NXPH4	neurexophilin 4	MYLK	myosin light chain kinase
OBP2B	odorant binding protein 2B	MYO16	myosin XVI
OBSCN-AS1	OBSCN antisense RNA 1	MYOM2	myomesin 2
OLFML2A	olfactomedin like 2A	NAV1	neuron navigator 1
OR4C46	olfactory receptor family 4 subfamily C member 46	NBEA	neurobeachin
OR4F29	olfactory receptor family 4 subfamily F member 29	NBEAL1	neurobeachin like 1
OSBPL5	oxysterol binding protein like 5	NBPF10	NBPF member 10
OSMR	oncostatin M receptor	NBPF8	NBPF member 8
OXR1	oxidation resistance 1	NCOR1P1	NCOR1 pseudogene 1
PABPC1P2	poly(A) binding protein cytoplasmic 1 pseudogene 2	NDE1	nudE neurodevelopment protein 1
PALLD	palladin, cytoskeletal associated protein	NDST1	N-deacetylase and N-sulfotransferase 1
PALM2AKAP2	PALM2 and AKAP2 fusion	NEK7	NIMA related kinase 7
PANK3	pantothenate kinase 3	NEO1	neogenin 1
PARP16	poly(ADP-ribose) polymerase family member 16	NEURL1	neuralized E3 ubiquitin protein ligase 1
PCDH18	protocadherin 18	NFIX	nuclear factor I X
PCSK5	proprotein convertase subtilisin/kexin type 5	NOTCH2	notch receptor 2
PDCD4-AS1	PDCD4 antisense RNA 1	NOTCH2NLA	notch 2 N-terminal like A
PDCD6IP	programmed cell death 6 interacting protein	NPAS2	neuronal PAS domain protein 2
PDE3A	phosphodiesterase 3A	NRARP	NOTCH regulated ankyrin repeat protein
PDE4B	phosphodiesterase 4B	NRG1	neuregulin 1
PDE4DIP	phosphodiesterase 4D interacting protein	NRP2	neuropilin 2
PDXK	pyridoxal kinase	NT5C2	5'-nucleotidase, cytosolic II
PDZRN3	PDZ domain containing ring finger 3	NT5DC3	5'-nucleotidase domain containing 3
PDZRN4	PDZ domain containing ring finger 4	NTAQ1	N-terminal glutamine amidase 1
PGAP1	post-GPI attachment to proteins inositol deacylase 1	NTF3	neurotrophin 3
PGBD2	piggyBac transposable element derived 2	NUFIP1	nuclear FMR1 interacting protein 1
PHLDA1	pleckstrin homology like domain family A member 1	NUP35	nucleoporin 35
PHYH	phytanoyl-CoA 2-hydroxylase	NUP50-DT	NUP50 divergent transcript
PHYHD1	phytanoyl-CoA dioxygenase domain containing 1	NXN	nucleoredoxin
PID1	phosphotyrosine interaction domain containing 1	OAZ2	ornithine decarboxylase antizyme 2
PITRM1	pitrilysin metallopeptidase 1	ODAPH	odontogenesis associated phosphoprotein
PKM	pyruvate kinase M1/2	OGFOD1	2-oxoglutarate and iron dependent oxygenase domain containing 1
PLCB4	phospholipase C beta 4	OR11H12	olfactory receptor family 11 subfamily H member 12
PLEKHG4B	pleckstrin homology and RhoGEF domain containing G4B	OR2A1	olfactory receptor family 2 subfamily A member 1
PLLP	plasmolipin	OR4C46	olfactory receptor family 4 subfamily C member 46
PLS3	plastin 3	OR4F17	olfactory receptor family 4 subfamily F member 17
PNOC	prepronociceptin	OR4F5	olfactory receptor family 4 subfamily F member 5
POPDC3	popeye domain containing 3	ORC5	origin recognition complex subunit 5
PPARG	peroxisome proliferator activated receptor gamma	OSBPL9	oxysterol binding protein like 9
PPCDC	phosphopantothenoylcysteine decarboxylase	OTOL1	otolin 1
PPP1R1C	protein phosphatase 1 regulatory inhibitor subunit 1C	PAK1	p21 (RAC1) activated kinase 1
PPP3CA	protein phosphatase 3 catalytic subunit alpha	PALLD	palladin, cytoskeletal associated protein
PRDM15	PR/SET domain 15	PAPOLG	poly(A) polymerase gamma
PREX1	phosphatidylinositol-3,4,5-trisphosphate dependent Rac exchange factor 1	PAPPA	pappalysin 1
PRIM2	DNA primase subunit 2	PBOV1	prostate and breast cancer overexpressed 1
PRKAG2	protein kinase AMP-activated non-catalytic subunit gamma 2	PCAT1	prostate cancer associated transcript 1
PRKAR1A	protein kinase cAMP-dependent type I regulatory subunit alpha	PCDH10	protocadherin 10
PRKCH	protein kinase C eta	PCNX1	pecanex 1
PRMT5	protein arginine methyltransferase 5	PDE1C	phosphodiesterase 1C
PRPSAP2	phosphoribosyl pyrophosphate synthetase associated protein 2	PDXDC2P-NPIPB14P	nuclear pore complex-interacting protein
PRR16	proline rich 16	PDZRN3	PDZ domain containing ring finger 3
PSMG4	proteasome assembly chaperone 4	PELI2	pellino E3 ubiquitin protein ligase family member 2
PTBP2	polypyrimidine tract binding protein 2	PGM5P2	phosphoglucomutase 5 pseudogene 2
PTPN11	protein tyrosine phosphatase non-receptor type 11	PGRMC2	progesterone receptor membrane component 2
PTPN12	protein tyrosine phosphatase non-receptor type 12	PHACTR2	phosphatase and actin regulator 2
PXN	paxillin	PHRF1	PHD and ring finger domains 1
QKI	QKI, KH domain containing RNA binding	PIGP	phosphatidylinositol glycan anchor biosynthesis class P
RAET1E-AS1	RAET1E antisense RNA 1	PITRM1	pitrilysin metallopeptidase 1
RAMP3	receptor activity modifying protein 3	PKP2	plakophilin 2
RAPGEF2	Rap guanine nucleotide exchange factor 2	PLXNA2	plexin A2
RASL11A	RAS like family 11 member A	PLXND1	plexin D1
RASSF9	Ras association domain family member 9	POM121L8P	POM121 transmembrane nucleoporin like 8, pseudogene
RCL1	RNA terminal phosphate cyclase like 1	POSTN	periostin

RCN1	reticulocalbin 1	POTEA	POTE ankyrin domain family member A
RGMB-AS1	RGMB antisense RNA 1	PPIAL4F	peptidylprolyl isomerase A like 4F
RGPD1	RANBP2 like and GRIP domain containing 1	PPM1D	protein phosphatase, Mg2+/Mn2+ dependent 1D
RHOU	ras homolog family member U	PPP1R14D	protein phosphatase 1 regulatory inhibitor subunit 14D
RMND1	required for meiotic nuclear division 1 homolog	PPP2R2A	protein phosphatase 2 regulatory subunit Balpha
RND3	Rho family GTPase 3	PRAG1	PEAK1 related, kinase-activating pseudokinase 1
RNF216	ring finger protein 216	PRDM1	PR/SET domain 1
RNF44	ring finger protein 44	PRDM2	PR/SET domain 2
ROCK1P1	Rho associated coiled-coil containing protein kinase 1 pseudogene 1	PRIM2	DNA primase subunit 2
RORA	RAR related orphan receptor A	PRIMPOL	primase and DNA directed polymerase
RPL29P2	ribosomal protein L29 pseudogene 2	PRKAG2	protein kinase AMP-activated non-catalytic subunit gamma 2
RRAS2	RAS related 2	PRKCH	protein kinase C eta
RRM2	ribonucleotide reductase regulatory subunit M2	PRR20D	proline rich 20D
RSL24D1	ribosomal L24 domain containing 1	PRR5L	proline rich 5 like
RTF2	replication termination factor 2	PRRX2	paired related homeobox 2
RUNX1	RUNX family transcription factor 1	PRSS23	serine protease 23
RYBP	RING1 and YY1 binding protein	PSMA5	proteasome 20S subunit alpha 5
S100A11	S100 calcium binding protein A11	PSMD14	proteasome 26S subunit, non-ATPase 14
SAP30L-AS1	SAP30L antisense RNA 1 (head to head)	PSTPIP2	proline-serine-threonine phosphatase interacting protein 2
SAR1A	secretion associated Ras related GTPase 1A	PTGER4	prostaglandin E receptor 4
SASH1	SAM and SH3 domain containing 1	PTGER4P2-CDK2AP2P2	PTGER4P2-CDK2AP2P2 readthrough, transcribed pseudogene
SBF2	SET binding factor 2	PTK7	protein tyrosine kinase 7 (inactive)
SCN1A	sodium voltage-gated channel alpha subunit 1	PTPRC	protein tyrosine phosphatase receptor type C
SDCBP2-AS1	SDCBP2 antisense RNA 1	PTPRE	protein tyrosine phosphatase receptor type E
SEC22B	SEC22 homolog B, vesicle trafficking protein	PTPRG	protein tyrosine phosphatase receptor type G
SEMA3A	semaphorin 3A	RAB3B	RAB3B, member RAS oncogene family
SEPTIN9	septin 9	RAB3GAP1	RAB3 GTPase activating protein catalytic subunit 1
SERPINB1	serpin family B member 1	RAI14	retinoic acid induced 14
SFI1	SFI1 centrin binding protein	RARB	retinoic acid receptor beta
SFTA1P	surfactant associated 1, lncRNA	RASSF3	Ras association domain family member 3
SH3D19	SH3 domain containing 19	RBM20	RNA binding motif protein 20
SH3PXD2A	SH3 and PX domains 2A	RBMS3	RNA binding motif single stranded interacting protein 3
SIN3A	SIN3 transcription regulator family member A	RBPJ	recombination signal binding protein for immunoglobulin kappa J region
SLC14A2	solute carrier family 14 member 2	RBPMS	RNA binding protein, mRNA processing factor
SLC22A16	solute carrier family 22 member 16	REEP3	receptor accessory protein 3
SLC22A23	solute carrier family 22 member 23	REXO1L1P	REXO1 like 1, pseudogene
SLC7A5P2	solute carrier family 7 member 5 pseudogene 2	REXO1L2P	REXO1 like 2, pseudogene
SMIM20	small integral membrane protein 20	RFC3	replication factor C subunit 3
SMYD4	SET and MYND domain containing 4	RFTN1	raftlin, lipid raft linker 1
SNAI2	snail family transcriptional repressor 2	RIN2	Ras and Rab interactor 2
SNAPC3	small nuclear RNA activating complex polypeptide 3	RNF38	ring finger protein 38
SNORA24	small nucleolar RNA, H/ACA box 24	RNU5F-1	RNA, U5F small nuclear 1
SNTB1	syntrophin beta 1	ROCK1	Rho associated coiled-coil containing protein kinase 1
SNX18	sorting nexin 18	RPL13AP17	ribosomal protein L13a pseudogene 17
SNX4	sorting nexin 4	RPTOR	regulatory associated protein of MTOR complex 1
SOCS6	suppressor of cytokine signaling 6	RTP3	receptor transporter protein 3
SOX5	SRY-box transcription factor 5	RUNX1	RUNX family transcription factor 1
SOX8	SRY-box transcription factor 8	RUSC2	RUN and SH3 domain containing 2
SPATA13-AS1	SPATA13 antisense RNA 1	SAMD4A	sterile alpha motif domain containing 4A
SPRY2	sprouty RTK signaling antagonist 2	SATB1	SATB homeobox 1
SQOR	sulfide quinone oxidoreductase	SCGB1A1	secretoglobin family 1A member 1
SRFBP1	serum response factor binding protein 1	SCUBE3	signal peptide, CUB domain and EGF like domain containing 3
SRI	sorcin	SEL1L3	SEL1L family member 3
STARD13	StAR related lipid transfer domain containing 13	SENP8	SUMO peptidase family member, NEDD8 specific
SULF1	sulfatase 1	SEPTIN11	septin 11
SYNE1	spectrin repeat containing nuclear envelope protein 1	SERINC3	serine incorporator 3
SYTL3	synaptotagmin like 3	SERINC5	serine incorporator 5
TANK	TRAF family member associated NFKB activator	SERPINE1	serpin family E member 1
TAOK3	TAO kinase 3	SERPINE3	serpin family E member 3
TAS2R41	taste 2 receptor member 41	SETDB1	SET domain bifurcated histone lysine methyltransferase 1
TBC1D14	TBC1 domain family member 14	SEZ6	seizure related 6 homolog
TBC1D20	TBC1 domain family member 20	SFRP4	secreted frizzled related protein 4
TBX18	T-box transcription factor 18	SFT2D3	SFT2 domain containing 3
TBX3	T-box transcription factor 3	SH2B2	SH2B adaptor protein 2
TENM4	teneurin transmembrane protein 4	SH3RF2	SH3 domain containing ring finger 2
TEX14	testis expressed 14, intercellular bridge forming factor	SIRPA	signal regulatory protein alpha
TGFBR2	transforming growth factor beta receptor 2	SLC14A2	solute carrier family 14 member 2
TGFBR3	transforming growth factor beta receptor 3	SLC15A1	solute carrier family 15 member 1
THEMIS	thymocyte selection associated	SLC1A2	solute carrier family 1 member 2
THSD1	thrombospondin type 1 domain containing 1	SLC25A51P1	SLC25A51 pseudogene 1
TIAM2	TIAM Rac1 associated GEF 2	SLC26A4	solute carrier family 26 member 4
TM4SF20	transmembrane 4 L six family member 20	SLC27A1	solute carrier family 27 member 1
TMC1	transmembrane channel like 1	SLC38A4	solute carrier family 38 member 4
TMCC1	transmembrane and coiled-coil domain family 1	SLC4A4	solute carrier family 4 member 4
TMCO3	transmembrane and coiled-coil domains 3	SLC6A10P	solute carrier family 6 member 10, pseudogene
TMEM108	transmembrane protein 108	SLC7A6	solute carrier family 7 member 6
TMEM14EP	transmembrane protein 14E, pseudogene	SMIM14	small integral membrane protein 14
TMEM170B	transmembrane protein 170B	SMIM21	small integral membrane protein 21
TMEM207	transmembrane protein 207	SMIM30	small integral membrane protein 30
TMEM217	transmembrane protein 217	SMNDC1	survival motor neuron domain containing 1
TMEM26	transmembrane protein 26	SMPD3	sphingomyelin phosphodiesterase 3
TNFAIP6	TNF alpha induced protein 6	SNAI2	snail family transcriptional repressor 2
TNP1	transition protein 1	SNAR-A3	small NF90 (ILF3) associated RNA A3
TOP3A	DNA topoisomerase III alpha	SNAR-B2	small NF90 (ILF3) associated RNA B2
TPPP	tubulin polymerization promoting protein	SNAR-C3	small NF90 (ILF3) associated RNA C3
TRAPPC9	trafficking protein particle complex subunit 9	SNHG3	small nucleolar RNA host gene 3
TRG-AS1	T cell receptor gamma locus antisense RNA 1	SNORA11B	small nucleolar RNA, H/ACA box 11B
TRHDE	thyrotropin releasing hormone degrading enzyme	SNRNP48	small nuclear ribonucleoprotein U11/U12 subunit 48
TRIB1	tribbles pseudokinase 1	SNTB1	syntrophin beta 1

TRIM25	tripartite motif containing 25
TSPAN2	tetraspanin 2
TTLL9	tubulin tyrosine ligase like 9
TUT7	terminal uridylyl transferase 7
TXNDC2	thioredoxin domain containing 2
TXNRD1	thioredoxin reductase 1
UBAP1	ubiquitin associated protein 1
UNC13B	unc-13 homolog B
USP33	ubiquitin specific peptidase 33
VMP1	vacuole membrane protein 1
WASF3	WASP family member 3
WDFY2	WD repeat and FYVE domain containing 2
WDR11	WD repeat domain 11
WDR74	WD repeat domain 74
WDR81	WD repeat domain 81
WEE1	WEE1 G2 checkpoint kinase
WIPI2	WD repeat domain, phosphoinositide interacting 2
WNT5A	Wnt family member 5A
WSB1	WD repeat and SOCS box containing 1
YJU2B	YJU2 splicing factor homolog B
YY1P2	YY1 transcription factor pseudogene 2
ZADH2	zinc binding alcohol dehydrogenase domain containing 2
ZBTB16	zinc finger and BTB domain containing 16
ZC3H15	zinc finger CCCH-type containing 15
ZCCHC2	zinc finger CCHC-type containing 2
ZDHHC14	zinc finger DHHC-type palmitoyltransferase 14
ZFP64	ZFP64 zinc finger protein
ZHX3	zinc fingers and homeoboxes 3
ZMPSTE24	zinc metallopeptidase STE24
ZNF227	zinc finger protein 227
ZNF33A	zinc finger protein 33A
ZNF444	zinc finger protein 444
ZNF469	zinc finger protein 469
ZNF521	zinc finger protein 521
ZNF703	zinc finger protein 703
ZNF860	zinc finger protein 860
ZNF875	zinc finger protein 875
ZRANB1	zinc finger RANBP2-type containing 1
ZSWIM6	zinc finger SWIM-type containing 6

SNX25	sorting nexin 25
SNX29	sorting nexin 29
SOX6	SRY-box transcription factor 6
SPAG16	sperm associated antigen 16
SPATS2L	spermatogenesis associated serine rich 2 like
SPIDR	scaffold protein involved in DNA repair
SPIN4	spindlin family member 4
SPINK4	serine peptidase inhibitor Kazal type 4
SPOP	speckle type BTB/POZ protein
SPRY3	sprouty RTK signaling antagonist 3
SPTBN5	spectrin beta, non-erythrocytic 5
SRGAP2D	SLIT-ROBO Rho GTPase activating protein 2D (pseudogene)
STC2	stanniocalcin 2
STIL	STIL centriolar assembly protein
STPG1	sperm tail PG-rich repeat containing 1
SUGCT	succinyl-CoA:glutarate-CoA transferase
SUSD1	sushi domain containing 1
SYNPO2	synaptopodin 2
SYNRG	synergin gamma
SYT15	synaptotagmin 15
TARS3	threonyl-tRNA synthetase 3
TBC1D19	TBC1 domain family member 19
TBC1D2B	TBC1 domain family member 2B
TCP11L1	t-complex 11 like 1
TDG	thymine DNA glycosylase
TEKT4P2	tektin 4 pseudogene 2
TENM3	teneurin transmembrane protein 3
TFEB	transcription factor EB
TGFBI	transforming growth factor beta induced
TGFBR2	transforming growth factor beta receptor 2
TGM2	transglutaminase 2
THEMIS	thymocyte selection associated
THOC3	THO complex 3
TIMM8B	translocase of inner mitochondrial membrane 8 homolog B
TJP1	tight junction protein 1
TLE3	TLE family member 3, transcriptional corepressor
TLE4	TLE family member 4, transcriptional corepressor
TMEM120B	transmembrane protein 120B
TMEM160	transmembrane protein 160
TMEM174	transmembrane protein 174
TMEM18	transmembrane protein 18
TNFRSF9	TNF receptor superfamily member 9
TNFSF4	TNF superfamily member 4
TNIP3	TNFAIP3 interacting protein 3
TNNI3K	TNNI3 interacting kinase
TNS1	tensin 1
TP53TG3B	TP53 target 3B
TPM1	tropomyosin 1
TPR	translocated promoter region, nuclear basket protein
TPRG1	tumor protein p63 regulated 1
TPTE	transmembrane phosphatase with tensin homology
TRIM32	tripartite motif containing 32
TRIM48	tripartite motif containing 48
TRIM49B	tripartite motif containing 49B
TSG101	tumor susceptibility 101
TSKU	tsukushi, small leucine rich proteoglycan
TTC13	tetratricopeptide repeat domain 13
TTC7A	tetratricopeptide repeat domain 7A
TTLL5	tubulin tyrosine ligase like 5
TTTY23	testis-specific transcript, Y-linked 23
TTTY3	testis-specific transcript, Y-linked 3
TUBB6	tubulin beta 6 class V
TXNRD1	thioredoxin reductase 1
UBE2MP1	ubiquitin conjugating enzyme E2 M pseudogene 1
UBE2U	ubiquitin conjugating enzyme E2 U
ULK4	unc-51 like kinase 4
USP10	ubiquitin specific peptidase 10
USP12	ubiquitin specific peptidase 12
USP17L11	ubiquitin specific peptidase 17 like family member 11
USP17L17	ubiquitin specific peptidase 17 like family member 17
USP17L20	ubiquitin specific peptidase 17 like family member 20
USP17L6P	ubiquitin specific peptidase 17 like family member 6, pseudogene
USP17L9P	ubiquitin specific peptidase 17 like family member 9, pseudogene
USP18	ubiquitin specific peptidase 18
UST-AS1	UST antisense RNA 1
UXS1	UDP-glucuronate decarboxylase 1
VAV3	vav guanine nucleotide exchange factor 3
VOPP1	VOPP1 WW domain binding protein
VRK2	VRK serine/threonine kinase 2
WNT5B	Wnt family member 5B
XCL2	X-C motif chemokine ligand 2
XRCC4	X-ray repair cross complementing 4
XYLT1	xylosyltransferase 1
ZBTB8OS	zinc finger and BTB domain containing 8 opposite strand
ZDHHC7	zinc finger DHHC-type palmitoyltransferase 7
ZDHHC8P1	ZDHHC8 pseudogene 1
ZFAT-AS1	ZFAT antisense RNA 1
ZFHX3	zinc finger homeobox 3
ZFP36L1	ZFP36 ring finger protein like 1

ZFPM2	zinc finger protein, FOG family member 2
ZMIZ1	zinc finger MIZ-type containing 1
ZMYND12	zinc finger MYND-type containing 12
ZNF438	zinc finger protein 438
ZNF469	zinc finger protein 469
ZNF503-AS1	ZNF503 antisense RNA 1
ZNF707	zinc finger protein 707
ZNF716	zinc finger protein 716
ZNF717	zinc finger protein 717
ZNF718	zinc finger protein 718
ZNF733P	zinc finger protein 733, pseudogene
ZNF74	zinc finger protein 74
ZNF875	zinc finger protein 875
ZNF92	zinc finger protein 92
ZXDA	zinc finger X-linked duplicated A

Supplementary Table 4: Genes and GO Terms Associated With Enhancer-Overlapping Intergenic Peaks Identified by ATAC sequencing in hSSCs Cultured with Wnt3a.

seqnames	start	end	width	strand	annotation	SYMBOL	ENSEMBL	Description	enhancerChr	enhancerStart	enhancerEnd	GO
chr5	14913053	14913736	684	*	Distal Intergenic	ANKH	ENSG00000154122	ANKH inorganic pyrophosphate transport regulator	chr5	14876890	15191750	skeletal system development
chr1	86094319	86095547	1229	*	Distal Intergenic	CCN1	ENSG00000142871	cellular communication network factor 1	chr1	86090600	86096760	skeletal system development
chr1	86072419	86073405	987	*	Distal Intergenic	CCN1	ENSG00000142871	cellular communication network factor 1	chr1	86068670	86082800	skeletal system development
chr16	64874896	64875555	660	*	Distal Intergenic	CDH11	ENSG00000140937	cadherin 11	chr16	64843950	64980680	skeletal system development
chr3	197098830	197099377	548	*	Distal Intergenic	DLG1	ENSG00000075711	discs large MAGUK scaffold protein 1	chr3	197031140	197236650	skeletal system development
chr4	145499866	145500243	378	*	Distal Intergenic	HHIP	ENSG00000164161	hedgehog interacting protein	chr4	145467640	145562140	skeletal system development
chr17	46559601	46560063	463	*	Distal Intergenic	HOXB1	ENSG00000120094	homeobox B1	chr17	46558440	46580340	skeletal system development
chr4	55409231	55409754	524	*	Distal Intergenic	KIT	ENSG00000157404	KIT proto-oncogene, receptor tyrosine kinase	chr4	55344200	55519090	skeletal system development
chr17	57930669	57931130	462	*	Distal Intergenic	MIR21	ENSG00000284190	microRNA 21	chr17	57928510	57931420	skeletal system development
chr5	149853652	149854015	364	*	Distal Intergenic	NDST1	ENSG00000070614	N-deacetylase and N-sulfotransferase 1	chr5	149834320	149855070	skeletal system development
chr5	40389721	40390105	385	*	Distal Intergenic	PTGER4	ENSG00000171522	prostaglandin E receptor 4	chr5	40053430	40675030	skeletal system development
chr8	49816029	49816437	409	*	Distal Intergenic	SNAI2	ENSG00000019549	snail family transcriptional repressor 2	chr8	49811010	49821940	skeletal system development
chr11	15908483	15908831	349	*	Distal Intergenic	SOX6	ENSG00000110693	SRY-box transcription factor 6	chr11	15726920	15987990	skeletal system development
chr5	135356015	135356488	474	*	Distal Intergenic	TGFBI	ENSG00000120708	transforming growth factor beta induced	chr5	135295720	135359580	skeletal system development
chr5	135356015	135356488	474	*	Distal Intergenic	TGFBI	ENSG00000120708	transforming growth factor beta induced	chr5	135354440	135357240	skeletal system development
chr3	30481601	30482055	455	*	Distal Intergenic	TGFBR2	ENSG00000163513	transforming growth factor beta receptor 2	chr3	30454200	30642990	skeletal system development
chr11	76481490	76482377	888	*	Distal Intergenic	TSKU	ENSG00000182704	tsukushi, small leucine rich proteoglycan	chr11	76478480	76484770	skeletal system development
chr11	76481490	76482377	888	*	Distal Intergenic	TSKU	ENSG00000182704	tsukushi, small leucine rich proteoglycan	chr11	76437830	76488350	skeletal system development
chr16	17609574	17610140	567	*	Distal Intergenic	XYLT1	ENSG00000103489	xylosyltransferase 1	chr16	17569740	17703350	skeletal system development
chr3	197098830	197099377	548	*	Distal Intergenic	DLG1	ENSG00000075711	discs large MAGUK scaffold protein 1	chr3	197031140	197236650	skeletal system morphogenesis
chr4	145499866	145500243	378	*	Distal Intergenic	HHIP	ENSG00000164161	hedgehog interacting protein	chr4	145467640	145562140	skeletal system morphogenesis
chr17	46559601	46560063	463	*	Distal Intergenic	HOXB1	ENSG00000120094	homeobox B1	chr17	46558440	46580340	skeletal system morphogenesis
chr5	149853652	149854015	364	*	Distal Intergenic	NDST1	ENSG00000070614	N-deacetylase and N-sulfotransferase 1	chr5	149834320	149855070	skeletal system morphogenesis
chr11	15908483	15908831	349	*	Distal Intergenic	SOX6	ENSG00000110693	SRY-box transcription factor 6	chr11	15726920	15987990	skeletal system morphogenesis
chr3	30481601	30482055	455	*	Distal Intergenic	TGFBR2	ENSG00000163513	transforming growth factor beta receptor 2	chr3	30454200	30642990	skeletal system morphogenesis
chr11	76481490	76482377	888	*	Distal Intergenic	TSKU	ENSG00000182704	tsukushi, small leucine rich proteoglycan	chr11	76478480	76484770	skeletal system morphogenesis
chr11	76481490	76482377	888	*	Distal Intergenic	TSKU	ENSG00000182704	tsukushi, small leucine rich proteoglycan	chr11	76437830	76488350	skeletal system morphogenesis
chr1	86094319	86095547	1229	*	Distal Intergenic	CCN1	ENSG00000142871	cellular communication network factor 1	chr1	86090600	86096760	osteoblast proliferation
chr1	86072419	86073405	987	*	Distal Intergenic	CCN1	ENSG00000142871	cellular communication network factor 1	chr1	86068670	86082800	osteoblast proliferation
chr22	27857390	27857887	498	*	Distal Intergenic	MN1	ENSG00000169184	MN1 proto-oncogene, transcriptional regulator	chr22	27580130	27893580	osteoblast proliferation
chr22	27852832	27853526	695	*	Distal Intergenic	MN1	ENSG00000169184	MN1 proto-oncogene, transcriptional regulator	chr22	27580130	27893580	osteoblast proliferation
chr1	86094319	86095547	1229	*	Distal Intergenic	CCN1	ENSG00000142871	cellular communication network factor 1	chr1	86090600	86096760	osteoblast differentiation
chr1	86072419	86073405	987	*	Distal Intergenic	CCN1	ENSG00000142871	cellular communication network factor 1	chr1	86068670	86082800	osteoblast differentiation
chr7	46184728	46185511	784	*	Distal Intergenic	IGFBP3	ENSG00000146674	insulin like growth factor binding protein 3	chr7	46183140	46187510	osteoblast differentiation
chr17	57930669	57931130	462	*	Distal Intergenic	MIR21	ENSG00000284190	microRNA 21	chr17	57928510	57931420	osteoblast differentiation
chr8	49816029	49816437	409	*	Distal Intergenic	SNAI2	ENSG00000019549	snail family transcriptional repressor 2	chr8	49811010	49821940	osteoblast differentiation

Supplementary Table 5: differentialy expressed genes identified by RNA sequencing.

Gene	Description	log fold change	log counts per million	Likelihood ratio	p Value	False discovery rate	signature
NTM	neurotrimin	1.563553907	5.30735022	248.3473909	5.95E-56	8.12E-52	up
IL7R	interleukin 7 receptor	-1.647583253	4.702525269	221.2483994	4.83E-50	3.30E-46	down
DKK2	dickkopf WNT signaling pathway inhibitor 2	2.291275194	2.726681444	148.3134379	4.05E-34	1.68E-30	up
PTX3	pentraxin 3	-1.243895017	9.460073336	147.9269536	4.92E-34	1.68E-30	down
COMP	cartilage oligomeric matrix protein	1.40214033	7.992587421	127.9717931	1.14E-29	3.11E-26	up
BAALC	BAALC binder of MAP3K1 and KLF4	1.69659658	2.582965171	114.6499137	9.39E-27	2.13E-23	up
PDZRN3	PDZ domain containing ring finger 3	1.108857177	5.006914122	110.3071557	8.39E-26	1.64E-22	up
NKD1	NKD inhibitor of WNT signaling pathway 1	2.033882629	2.69481674	101.5090632	7.11E-24	1.21E-20	up
STEAP4	STEAP4 metalloreductase	-1.491569976	3.357449149	97.37962149	5.72E-23	8.67E-20	down
ADAMTS14	ADAM metallopeptidase with thrombospondin type 1 motif 14	1.51190593	3.587074068	96.67185769	8.18E-23	1.12E-19	up
NDNF	neuron derived neurotrophic factor	1.532828026	5.088423144	94.94642116	1.96E-22	2.43E-19	up
AXIN2	axin 2	1.326866667	4.256446038	93.45119266	4.16E-22	4.73E-19	up
CABLES1	Cdk5 and Abl enzyme substrate 1	1.015368303	4.584532206	87.54321827	8.25E-21	8.03E-18	up
CXCL12	C-X-C motif chemokine ligand 12	-1.122815387	4.964029511	81.30106131	1.94E-19	1.76E-16	down
ANKRD30BL	ankyrin repeat domain 30B like	-2.247453234	1.479586091	78.58672394	7.66E-19	6.53E-16	down
FOXQ1	forkhead box Q1	1.222385017	2.944443418	73.91724558	8.15E-18	6.54E-15	up
GMNC	geminin coiled-coil domain containing	-1.497544597	2.946010351	68.48039829	1.28E-16	9.20E-14	down
KRT81	keratin 81	1.676410937	3.009090741	64.24349458	1.10E-15	7.14E-13	up
FRMD5	FERM domain containing 5	1.290850415	2.369707417	62.62651355	2.50E-15	1.55E-12	up
CDH6	cadherin 6	1.007677112	5.630095684	62.0908618	3.28E-15	1.95E-12	up
STRA6	signaling receptor and transporter of retinol STRA6	1.624623439	1.559962736	60.53405392	7.23E-15	3.95E-12	up
CTHRC1	collagen triple helix repeat containing 1	1.024923527	6.399235473	57.88413337	2.78E-14	1.40E-11	up
NXPH3	neurexophilin 3	1.250856885	4.50936026	50.95556785	9.45E-13	4.03E-10	up
MIR663AHG	MIR663A host gene	-2.656994656	0.508194167	50.97561241	9.35E-13	4.03E-10	down
PCSK6	proprotein convertase subtilisin/kexin type 6	1.585642447	1.486353328	46.99723867	7.11E-12	2.69E-09	up
RAB5IF	RAB5 interacting factor	-1.983624584	1.217878425	42.69175457	6.41E-11	2.19E-08	down
SUGCT	succinyl-CoA:glutarate-CoA transferase	1.30340244	4.566458379	42.15694363	8.42E-11	2.71E-08	up
ZBED2	zinc finger BED-type containing 2	2.878751898	0.389260317	41.90887244	9.56E-11	2.91E-08	up
CIDEC	cell death inducing DFFA like effector c	-1.219497136	1.857069552	40.84642829	1.65E-10	4.78E-08	down
ANO1	anoctamin 1	1.45242648	2.653239076	40.41807801	2.05E-10	5.59E-08	up
ADGRG2	adhesion G protein-coupled receptor G2	-1.049046843	1.788007777	32.83031899	1.01E-08	2.18E-06	down
STAB2	stabilin 2	1.483362522	0.952543355	31.39076808	2.11E-08	4.30E-06	up
LINC01592	long intergenic non-protein coding RNA 1592	1.21371667	1.968019669	29.0982845	6.88E-08	1.21E-05	up
EPHA3	EPH receptor A3	-1.017654889	1.882794503	28.56755385	9.05E-08	1.47E-05	down
LGR5	leucine rich repeat containing G protein-coupled receptor 5	2.393596697	-0.751168454	28.21751606	1.08E-07	1.72E-05	up
TMEM160	transmembrane protein 160	-1.239441118	2.406012006	28.13683802	1.13E-07	1.75E-05	down
HNF4G	hepatocyte nuclear factor 4 gamma	-3.251403158	-1.392848068	27.64232761	1.46E-07	2.12E-05	down
MME	membrane metalloendopeptidase	-1.008646532	4.292226811	25.93114318	3.54E-07	4.73E-05	down
PLD5	phospholipase D family member 5	1.07221591	1.24972707	24.45099009	7.62E-07	9.54E-05	up
AKR1B10	aldo-keto reductase family 1 member B10	-1.500614433	0.332115202	24.28548122	8.31E-07	1.03E-04	down
FTH1	ferritin heavy chain 1	-1.416900331	8.593579075	23.98112282	9.73E-07	1.17E-04	down
FAM86HP	family with sequence similarity 86, member A pseudogene	-1.489184467	0.660562406	23.89472607	1.02E-06	1.20E-04	down
RASSF2	Ras association domain family member 2	1.061242767	1.19353947	22.69433019	1.90E-06	2.12E-04	up
ABCA9	ATP binding cassette subfamily A member 9	-1.353087546	0.611107762	22.10482772	2.58E-06	2.67E-04	down
PARM1	prostate androgen-regulated mucin-like protein 1	-1.359973013	0.41149288	21.66517127	3.25E-06	3.28E-04	down
RN7SK	RNA component of 7SK nuclear ribonucleoprotein	-1.535507861	1.378768226	20.92990377	4.76E-06	4.64E-04	down
EBF2	EBF transcription factor 2	-1.292052123	0.456849378	20.78237975	5.15E-06	4.94E-04	down
SERTAD4-AS1	SERTAD4 antisense RNA 1	1.650852315	0.172717606	20.65742412	5.49E-06	5.20E-04	up
CXCL2	C-X-C motif chemokine ligand 2	-2.027190375	-0.660557275	20.47181715	6.05E-06	5.65E-04	down
DOK7	docking protein 7	1.220106622	0.811852318	19.99946087	7.75E-06	7.00E-04	up
TSPAN2	tetraspanin 2	1.815694243	-0.059611917	19.8887738	8.21E-06	7.27E-04	up
AKAP5	A-kinase anchoring protein 5	1.193326961	0.702767779	19.7619926	8.77E-06	7.72E-04	up
ANGPTL5	angiopoietin like 5	-2.349796202	-1.27353401	19.71234568	9.00E-06	7.75E-04	down
NCF2	neutrophil cytosolic factor 2	1.998021889	-0.533054154	19.4581731	1.03E-05	8.71E-04	up
BATF	basic leucine zipper ATF-like transcription factor	1.153357695	1.049674382	18.25864335	1.93E-05	1.58E-03	up
TMEM155	NULL	-1.370270888	1.056006897	17.17383256	3.41E-05	2.58E-03	down
LIPG	lipase G, endothelial type	1.812532931	0.348368852	14.79130878	0.000120088	7.25E-03	up
TBILA	TGF-beta induced lncRNA	1.21432341	0.250000737	14.73359286	0.00012382	7.44E-03	up
HGF	hepatocyte growth factor	-1.354266467	0.680215526	14.63872169	0.000130211	7.76E-03	down
KDR	kinase insert domain receptor	-1.918343453	-1.021084485	14.40156628	0.000147679	8.57E-03	down
ISLR2	immunoglobulin superfamily containing leucine rich repeat 2	1.942960567	-1.276332376	14.32604892	0.000153723	8.81E-03	up
RMRP	RNA component of mitochondrial RNA processing endoribonuclease	-1.211925283	0.718114244	14.12720916	0.000170854	9.55E-03	down
SNORD87	small nucleolar RNA, C/D box 87	1.979381738	-1.255644872	14.06823584	0.000176296	9.78E-03	up
KRT31	keratin 31	2.089607823	-1.295724752	13.30984435	0.000264016	1.35E-02	up
REM1	RRAD and GEM like GTPase 1	-1.343003369	-0.380185412	13.11643325	0.000292717	1.47E-02	down
GNAZ	G protein subunit alpha z	1.050730637	1.349718783	13.07823198	0.000298747	1.49E-02	up
LINC01852	long intergenic non-protein coding RNA 1852	-1.840279741	-1.039097146	12.77903832	0.000350525	1.68E-02	down
MT1M	metallothionein 1M	1.05197953	1.073265995	12.74960855	0.000356084	1.69E-02	up
MIR4523	microRNA 4523	-2.157027128	-0.031856025	12.49121596	0.00040887	1.89E-02	down
RAPGEF4	Rap guanine nucleotide exchange factor 4	1.563350879	-0.767886251	12.48162005	0.000410976	1.89E-02	up
IFIT2	interferon induced protein with tetratricopeptide repeats 2	-1.14438177	0.454485789	12.25652967	0.000463633	2.09E-02	down
ZSCAN12P1	zinc finger and SCAN domain containing 12 pseudogene 1	-1.133426843	0.323565157	11.7530383	0.00060744	2.60E-02	down
MAP2K6	mitogen-activated protein kinase kinase 6	-1.410656946	-0.247913164	11.68242453	0.000630933	2.69E-02	down

LINC02604	long intergenic non-protein coding RNA 2604	-1.096263344	0.493592939	11.52224293	0.000687684	2.83E-02	down
DDIT4L	DNA damage inducible transcript 4 like	1.617858783	0.008385249	11.47465142	0.000705519	2.90E-02	up
CYP26B1	cytochrome P450 family 26 subfamily B member 1	-1.230755831	-0.279543856	11.46292745	0.000709984	2.91E-02	down
RDH14	retinol dehydrogenase 14	-1.482494244	0.253157754	11.20673754	0.000815009	3.27E-02	down
PDZRN3-AS1	PDZRN3 antisense RNA 1	1.006941843	0.503225072	10.99123329	0.000915439	3.63E-02	up
LOC100133091	NULL	-1.010167646	0.807722432	10.95048223	0.000935793	3.70E-02	down
STOX2	storkhead box 2	-1.315895328	0.172501786	10.94681199	0.000937648	3.70E-02	down
HECW1	HECT, C2 and WW domain containing E3 ubiquitin protein ligase 1	-1.125702243	0.24351271	10.8366602	0.000995101	3.88E-02	down
PLCXD2	phosphatidylinositol specific phospholipase C X domain containing 2	1.02017507	0.368341422	10.79521704	0.001017627	3.92E-02	up
TSPAN32	tetraspanin 32	1.765438496	-1.118062734	10.65084377	0.001100207	4.22E-02	up
EGF	epidermal growth factor	-1.022532186	0.305232641	10.15462292	0.001439398	5.17E-02	down
IL15RA	interleukin 15 receptor subunit alpha	-1.222119629	-0.405110797	10.102298	0.001480846	5.26E-02	down
IL18R1	interleukin 18 receptor 1	-1.527995333	-1.251666216	9.71380311	0.001828891	6.25E-02	down
IRX1	iroquois homeobox 1	1.940402307	0.360291996	9.468423594	0.002090389	6.99E-02	up
HHEX	hematopoietically expressed homeobox	-1.078695894	-0.381102794	9.36494042	0.002211752	7.32E-02	down
NPAS4	neuronal PAS domain protein 4	1.413033451	0.58326651	9.067533387	0.002601878	8.22E-02	up
EREG	epiregulin	-1.078021959	-0.107829869	8.747836214	0.003099696	9.37E-02	down
SPRY4-AS1	SPRY4 antisense RNA 1	-1.316394563	-0.886844107	8.606619568	0.003349434	9.88E-02	down
CLYBL	citramalyl-CoA lyase	1.569487027	-1.295654086	8.562836519	0.003430944	1.00E-01	up
MIR924HG	MIR924 host gene	-1.379224738	-1.097593401	8.539469171	0.00347527	1.01E-01	down
EPS8L1	EPS8 signaling adaptor L1	1.021775833	-0.132226364	8.523397041	0.003506094	1.01E-01	up
SNAI3	snail family transcriptional repressor 3	-1.324829496	-0.526228124	8.454922065	0.003640568	1.04E-01	down
SPON1	spondin 1	-1.133272529	0.585810017	8.343480057	0.003870738	1.08E-01	down
PKP2	plakophilin 2	1.561351521	-0.637770975	8.278246585	0.004012286	1.11E-01	up
RETREG1	reticulophagy regulator 1	1.243007803	-0.691656464	8.220535501	0.004141898	1.14E-01	up
MYH3	myosin heavy chain 3	1.051957994	1.474928313	8.068808983	0.004503371	1.21E-01	up
LINC01123	long intergenic non-protein coding RNA 1123	-1.391616365	-0.868816058	7.886260534	0.004981175	1.30E-01	down
CEBPA	CCAAT enhancer binding protein alpha	-1.467949168	0.066280259	7.789323233	0.005255588	1.34E-01	down
ADRA1D	adrenoceptor alpha 1D	1.212657563	0.25600917	7.764098229	0.00532949	1.36E-01	up
MMP28	matrix metallopeptidase 28	-1.196906756	-0.655164127	7.716216481	0.005472697	1.39E-01	down
HSD11B1	hydroxysteroid 11-beta dehydrogenase 1	-1.182297876	-0.720649721	7.671420433	0.005610228	1.41E-01	down
KIF6	kinesin family member 6	-1.292151833	-0.73749504	7.613762344	0.00579245	1.44E-01	down
HAGLR	HOXD antisense growth-associated long non-coding RNA	-1.073974885	-0.405629542	7.53252945	0.006059477	1.48E-01	down
TRPM3	transient receptor potential cation channel subfamily M member 3	-1.398511877	-1.186937836	7.499262216	0.006172427	1.49E-01	down
FAM221A	family with sequence similarity 221 member A	1.472787136	-0.731440408	7.402447914	0.006513518	1.56E-01	up
FAM13A-AS1	FAM13A antisense RNA 1	-1.121904507	-0.86957469	7.373563499	0.006618964	1.58E-01	down
NPY2R	neuropeptide Y receptor Y2	-1.59716297	-0.533622112	7.241780153	0.007122633	1.68E-01	down
LOC100289495	uncharacterized LOC100289495	1.138405337	-0.675617518	7.204352706	0.007272698	1.70E-01	up
ASB14	ankyrin repeat and SOCS box containing 14	-1.182872183	-0.962285748	6.96088307	0.00833109	1.88E-01	down
RPL23AP64	ribosomal protein L23a pseudogene 64	-1.049203577	-0.130298611	6.614931393	0.010112723	2.12E-01	down
DPP4	dipeptidyl peptidase 4	-1.197392499	0.458699433	6.572449502	0.010356931	2.15E-01	down
PDE11A	phosphodiesterase 11A	1.070827532	0.222420328	6.451624342	0.011085095	2.26E-01	up
NPIPB4	nuclear pore complex interacting protein family member B4	-1.074498407	0.401653779	6.251408449	0.01240946	2.41E-01	down
CYP4F35P	cytochrome P450 family 4 subfamily F member 35, pseudogene	1.05175713	-0.802562004	6.223960028	0.012603291	2.43E-01	up
SNORD36C	small nucleolar RNA, C/D box 36C	-1.133415054	-0.781245538	6.219597145	0.012634386	2.43E-01	down
ODF3B	NULL	-1.019456561	-0.609392631	6.210335229	0.012700658	2.43E-01	down
EEF1A2	eukaryotic translation elongation factor 1 alpha 2	1.174493165	-0.488202854	6.058789346	0.013837251	2.60E-01	up
LOC644634	uncharacterized LOC644634	1.09573608	-0.660556789	6.052004439	0.01389052	2.60E-01	up
CYP2T1P	cytochrome P450 family 2 subfamily T member 1, pseudogene	-1.072535876	-0.908309686	6.022531092	0.014124376	2.63E-01	down
DLG2	discs large MAGUK scaffold protein 2	-1.055993567	-0.536024299	5.876915916	0.01534067	2.77E-01	down
PIH1D2	PIH1 domain containing 2	-1.186661173	-0.359517924	5.817272966	0.015869531	2.81E-01	down
HOPX	HOP homeobox	1.058781122	-0.720737479	5.765877966	0.016340319	2.86E-01	up
GLYATL1	glycine-N-acyltransferase like 1	-1.083748191	-1.080022824	5.706895284	0.0168984	2.91E-01	down
RASSF1-AS1	RASSF1 antisense RNA 1	1.096361885	-1.18485976	5.63663325	0.017588951	2.98E-01	up
RAP1GAP	RAP1 GTPase activating protein	1.043556531	-0.611848812	5.460127988	0.019455222	3.18E-01	up
LINC00513	long intergenic non-protein coding RNA 513	-1.3309247	-0.814593341	5.338302912	0.020861773	3.32E-01	down
NPIPB11	nuclear pore complex interacting protein family member B11	-1.344125423	0.110141364	5.255030445	0.021883418	3.41E-01	down
F12	coagulation factor XII	-1.01504281	-0.733720988	5.23050856	0.022194042	3.44E-01	down
MAPK10	mitogen-activated protein kinase 10	-1.044599138	-1.038583009	5.1811082	0.022833756	3.51E-01	down
PLXDC1	plexin domain containing 1	1.335143816	-0.04877284	5.094352999	0.024003867	3.60E-01	up
LINC01152	long intergenic non-protein coding RNA 1152	-1.09551426	-0.855191129	4.987203215	0.025535449	3.76E-01	down
TCTE3	NULL	1.024152538	-0.628647564	4.772619259	0.028915801	4.01E-01	up
LINC00619	long intergenic non-protein coding RNA 619	-1.059902266	-0.8811373	4.749528308	0.029306327	4.04E-01	down
ANXA2P3	annexin A2 pseudogene 3	1.391960058	1.358877906	4.704912269	0.030076537	4.08E-01	up
M1AP	meiosis 1 associated protein	-1.164202645	-0.980078791	4.567738057	0.032579556	4.22E-01	down
LOC101928119	uncharacterized LOC101928119	1.223801953	-0.792148925	4.111501315	0.042592537	4.90E-01	up
KCND2	potassium voltage-gated channel subfamily D member 2	-1.077773921	-0.740565614	4.109107031	0.042652877	4.91E-01	down
CATSPER3	cation channel sperm associated 3	1.123294525	-1.20729935	4.098274509	0.042927002	4.91E-01	up
FGF18	fibroblast growth factor 18	1.175026537	-0.216144378	4.077420086	0.043459968	4.93E-01	up

Supplementary Table 6: Genes Exhibiting Significant Changes in H3K27me3 Deposition Identified by CUT&Tag in hSSCs Cultured with Wnt3a.

Gene	Description	Active Wnt3a	Inactive Wnt3a	Fold change
5_8S_rRNA	NULL	0.060732389	0.231909503	-1.933023922
AB231739	NULL	0.110860423	0.04233481	1.388828098
AB372727	NULL	0.427092405	0.161483223	1.403163961
AB488780	NULL	0.090599999	0.186034834	-1.03798984
ACTR3BP5	ACTR3B pseudogene 5	0.056475517	0.138532204	-1.294523915
ACTRT1	actin related protein T1	0.03582424	0.118905151	-1.730803225
ADAM1A	ADAM metallopeptidase domain 1A (pseudogene)	0.07043469	0.16388471	-1.218323206
ADAM3A	ADAM metallopeptidase domain 3A (pseudogene)	0.00792807	0.002065779	1.940284129
AF188711	NULL	0.047993064	0.180341258	-1.909831675
AF339817	NULL	0.079263963	0.160659988	-1.019273669
AF387615	NULL	0.863269215	0.418299245	1.045275147
AF548120	NULL	0.225723943	0.056934545	1.987183275
AJ004954	NULL	0.420834129	0.155634731	1.435087666
AJ276246	NULL	0.054211131	0.397937654	-2.87588142
AJ515158	NULL	0.152096145	0.068527434	1.150230021
AK001057	NULL	0.088818483	0.217179501	-1.289956098
AK001928	NULL	0.053480147	0.21872197	-2.032022806
AK021537	NULL	0.0490242	0.101127647	-1.044611465
AK021977	NULL	0.081379739	0.207692568	-1.351708043
AK022997	NULL	0.040863398	0.105980476	-1.374917431
AK025288	NULL	0.080728314	0.168664388	-1.063008723
AK026244	NULL	0.136535245	0.304169457	-1.155601879
AK026512	NULL	0.108128082	0.228642056	-1.080349546
AK055957	NULL	0.110229039	0.242345106	-1.136558605
AK056505	NULL	0.083959088	0.169999727	-1.017774025
AK094621	NULL	0.046065193	0.095662904	-1.054282545
AK094990	NULL	0.061583695	0.190420545	-1.628568819
AK096233	NULL	0.068437761	0.145620534	-1.089349333
AK096898	NULL	0.0898586	0.214379745	-1.254440114
AK098481	NULL	0.084830618	0.213639618	-1.332522232
AK098783	NULL	0.03296559	0.090656553	-1.459450397
AK124433	NULL	0.118106822	0.042317441	1.480768017
AK124950	NULL	0.10539721	0.232312158	-1.140227982
AK127697	NULL	0.031663344	0.067981759	-1.102334076
AK130481	NULL	0.042626275	0.116550038	-1.451134582
AK299432	NULL	0.038030708	0.085868916	-1.174971201
AK304759	NULL	0.058824578	0.304521453	-2.372052908
AK308652	NULL	0.10796317	0.22052091	-1.030376214
AK310046	NULL	0.112830717	0.297204138	-1.39729432
AL833150	NULL	0.124709694	0.361138931	-1.533980342
ALYREF	Aly/REF export factor	0.131182621	0.30453892	-1.215050014
AMN1	antagonist of mitotic exit network 1 homolog	0.408986829	0.820356152	-1.004195998
ANKRD36BP1	ankyrin repeat domain 36B pseudogene 1	0.045238358	0.126948007	-1.488619275
ANXA1	annexin A1	0.25973698	0.527359908	-1.021736464
APOBEC4	apolipoprotein B mRNA editing enzyme catalytic polypeptide like 4	0.065535646	0.282167863	-2.106201949
ARGFXP2	arginine-fifty homeobox pseudogene 2	0.186119632	0.079481527	1.227538741
ARL4D	ADP ribosylation factor like GTPase 4D	0.72644136	0.284965261	1.350060291
AX747080	NULL	0.157667907	0.343853799	-1.124906251
AX747098	NULL	0.122454307	0.245045005	-1.000803221
AX747125	NULL	0.136337638	0.418559983	-1.618250493
AX747333	NULL	0.025200722	0.091914457	-1.866826722
AX747345	NULL	0.045572647	0.122515907	-1.426729017
AX747376	NULL	0.094417052	0.193898145	-1.038179658
AX747756	NULL	0.03811091	0.173115223	-2.183456629
AX747838	NULL	0.160128366	0.336692968	-1.072204693
AX748016	NULL	0.122587962	0.25423063	-1.052320543
AX748371	NULL	0.019991522	0.078235913	-1.968442686
AY940074	NULL	0.106394186	0.052437286	1.020754386
BACE1-AS	BACE1 antisense RNA	0.092772219	0.187254354	-1.013234514
BC018887	NULL	0.382901644	0.185177406	1.04806577
BC020935	NULL	0.044767582	0.113181812	-1.338115838
BC021736	NULL	0.208406027	0.417023934	-1.000733184
BC024027	NULL	0.024338998	0.054219423	-1.155540014
BC024169	NULL	0.07843726	0.037116284	1.079486872
BC029038	NULL	0.101758378	0.243328688	-1.257758842
BC033323	NULL	0.051077598	0.120631583	-1.239845101
BC034630	NULL	0.052784741	0.123167679	-1.222430894

BC036258	NULL	0.056163501	0.115689703	-1.042555681
BC037357	NULL	0.146613495	0.30164482	-1.040832906
BC037382	NULL	0.077220335	0.1636892	-1.08390642
BC037783	NULL	0.206877908	0.100538109	1.041037129
BC038576	NULL	0.037812256	0.144267629	-1.931821784
BC038779	NULL	0.062765429	0.132058666	-1.073136938
BC039319	NULL	0.125110844	0.050421135	1.31110635
BC039399	NULL	0.122106638	0.036006842	1.761798653
BC039452	NULL	0.029285222	0.069904792	-1.255218525
BC042029	NULL	0.040384231	0.101124725	-1.324271799
BC042378	NULL	0.054616801	0.132631409	-1.280005741
BC042433	NULL	0.040479773	0.088543435	-1.129184153
BC043220	NULL	0.162909873	0.079659666	1.032152713
BC043587	NULL	0.067257217	0.146868366	-1.126762698
BC048132	NULL	0.033292476	0.144858727	-2.121378544
BC048420	NULL	0.044806014	0.092803754	-1.050490769
BC048997	NULL	0.079012277	0.272345541	-1.785289504
BC051736	NULL	0.127196952	0.29135551	-1.1957165
BC058002	NULL	0.108975065	0.275526068	-1.338190764
BC062350	NULL	0.063576146	0.177664603	-1.482598818
BC062762	NULL	0.130767375	0.269017077	-1.040695107
BC062763	NULL	0.044320091	0.145775488	-1.71771542
BC067269	NULL	0.03919851	0.097300994	-1.311655745
BC069792	NULL	0.154275875	0.059459071	1.375543662
BC069804	NULL	0.033163217	0.118474901	-1.836925588
BC069815	NULL	0.123234277	0.266157905	-1.110878828
BC101420	NULL	0.075655711	0.165772019	-1.131679626
BC113958	NULL	0.077897893	0.213968508	-1.457742264
BC114455	NULL	0.101442009	0.226934297	-1.161619444
BC122864	NULL	0.173045981	0.379792468	-1.134055859
BC127854	NULL	0.118005731	0.310291739	-1.394768368
BC131768	NULL	0.098277229	0.240326063	-1.290064034
BC146941	NULL	0.122565702	0.25195369	-1.039603268
BC150500	NULL	0.129893645	0.290564804	-1.161529111
BC150503	NULL	0.12445571	0.043677457	1.510671666
BIRC8	baculoviral IAP repeat containing 8	0.091650411	0.18341325	-1.000884617
C11orf71	chromosome 11 open reading frame 71	0.171127285	0.374301668	-1.12913167
C1GALT1C1	C1GALT1 specific chaperone 1	0.092161635	0.26967321	-1.548973997
C7orf66	NULL	0.020731415	0.081323839	-1.971859767
C8orf48	chromosome 8 open reading frame 48	0.177516844	0.084934684	1.063530204
CDH19	cadherin 19	0.114734614	0.233913323	-1.027673336
CDR1	cerebellar degeneration related 1	0.03763393	0.017975891	1.065970712
CFH	complement factor H	0.154032184	0.319003615	-1.050340947
CITED2	Cbp/p300 interacting transactivator with Glu/Asp rich carboxy-terminal domain 2	0.636472132	0.317640826	1.002700996
CoTC_ribozyme	NULL	0.220673639	0.606322954	-1.458172143
CRISPR_DR35	NULL	0.033475231	0.103119146	-1.623146319
CYCSP52	CYCS pseudogene 52	0.274184945	0.090248835	1.603169152
D21S2088E	D21S2088E	0.024234325	0.055065	-1.184083716
D28408	NULL	0.457933294	0.225239609	1.02367691
DCAF12L1	DDB1 and CUL4 associated factor 12 like 1	0.057644855	0.21633513	-1.908004215
DCAF4L2	DDB1 and CUL4 associated factor 4 like 2	0.035731375	0.101562122	-1.507099119
DCAF8L1	DDB1 and CUL4 associated factor 8 like 1	0.051216463	0.10375354	-1.018481039
DCAF8L2	DDB1 and CUL4 associated factor 8 like 2	0.043718287	0.119448205	-1.450076395
DD413568	NULL	0.546685008	1.107027017	-1.017908713
DD413619	NULL	0.048452154	0.347669098	-2.843082145
DD413694	NULL	0.205977519	0.515657055	-1.323925013
DEFB112	defensin beta 112	0.068006859	0.148233414	-1.124118534
DEFB113	defensin beta 113	0.049704741	0.016646279	1.578183788
DEFB114	defensin beta 114	0.065261126	0.032446689	1.008152624
DEFB115	defensin beta 115	0.055236337	0.157416143	-1.510893941
DEFB133	defensin beta 133 (pseudogene)	0.056988845	0.027535231	1.049400829
DIRC1	disrupted in renal carcinoma 1	0.054281306	0.201766528	-1.894159531
DJ439526	NULL	0.089901506	0.189622375	-1.076712025
DJ439531	NULL	0.102699005	0.451366072	-2.13587578
DJ439558	NULL	0.204083188	0.025062841	3.0255355
DJ439576	NULL	0.352297027	0.022034266	3.998971554
DKFZp686M11215	NULL	0.077028714	0.194564421	-1.336779681
DKFZp686N1631	NULL	0.110771368	0.251002151	-1.180114701
DKFZp761D112	NULL	0.101091386	0.047850282	1.079060753
DL490275	NULL	0.124045611	0.061101535	1.021590156

DL490795	NULL	0.134141463	0.042665049	1.652628649
DL490813	NULL	0.03855668	0.112943503	-1.550548547
DL490859	NULL	0.165695553	0.351444885	-1.084763577
DL490879	NULL	0.055887146	0.13734154	-1.297179633
DL491467	NULL	0.866473346	0.15085064	2.522034558
DL491527	NULL	0.107646755	0.283412505	-1.396598592
DL491652	NULL	0.099444987	0.534910481	-2.427326919
DL492006	NULL	0.110160717	0.242511664	-1.138444287
DL492217	NULL	0.791973495	0.242007642	1.710399546
DL492700	NULL	0.178207646	0.371475984	-1.05970971
DM376721	NULL	0.350464942	0.03750735	3.224024891
DPRXP4	divergent-paired related homeobox pseudogene 4	0.069045445	0.13843654	-1.003606643
DQ569978	NULL	0.312768541	0.105000634	1.574697376
DQ570500	NULL	0.330459902	0.031367639	3.3971264
DQ570601	NULL	0.458646372	0.213121073	1.105708978
DQ570642	NULL	0.589676879	0.2622386	1.169044572
DQ570720	NULL	0.12317615	0.259418722	-1.074559657
DQ570895	NULL	0.438766261	1.30509249	-1.572627553
DQ571071	NULL	0.086892982	0.251923188	-1.535672353
DQ571132	NULL	0.354227006	0.886694841	-1.323763477
DQ571386	NULL	0.287236273	0.727448363	-1.340606896
DQ571464	NULL	0.542071576	0.261387007	1.052295929
DQ571799	NULL	0.08929529	0.180780002	-1.017579113
DQ572701	NULL	0.30553405	0.104991942	1.541054562
DQ572925	NULL	0.074612761	0.151558968	-1.022384916
DQ572928	NULL	0.069085573	0.452582616	-2.711724809
DQ573195	NULL	0.672715047	0.301575426	1.157476655
DQ573199	NULL	0.289237773	0.081210918	1.832510378
DQ573285	NULL	0.38469328	0.046301942	3.054564032
DQ573774	NULL	0.076306607	0.209892577	-1.459771259
DQ573850	NULL	0.208736397	0.845369015	-2.017899051
DQ574199	NULL	0.163712185	0.594469757	-1.86044171
DQ574255	NULL	0.302445021	0.697584707	-1.205695501
DQ574380	NULL	0.384314563	0.12227695	1.652135178
DQ574672	NULL	0.135505611	0.365557865	-1.4317472
DQ575099	NULL	0.383418515	0.783292438	-1.030631025
DQ575498	NULL	0.161340846	0.35590296	-1.141372206
DQ575984	NULL	0.12359983	0.578765934	-2.227303253
DQ576994	NULL	0.108769916	0.228158242	-1.068755192
DQ577052	NULL	0.212177545	0.561085556	-1.402948794
DQ577171	NULL	0.13273313	0.412107578	-1.634492482
DQ577219	NULL	0.199924348	0.074853956	1.41730372
DQ577349	NULL	0.61875727	0.206225402	1.585151522
DQ577365	NULL	0.154119602	0.357510798	-1.213936451
DQ577420	NULL	0.134877159	0.510338164	-1.919807478
DQ577458	NULL	0.089282152	0.322610282	-1.853348719
DQ577480	NULL	0.068394674	0.22518314	-1.719142931
DQ577683	NULL	0.086892976	0.219430159	-1.336450357
DQ578031	NULL	0.758394931	1.563129843	-1.043416396
DQ578873	NULL	0.930286405	1.92276034	-1.047432102
DQ578901	NULL	0.109180018	0.308557894	-1.498832362
DQ579129	NULL	0.473253484	1.162963755	-1.297121104
DQ579268	NULL	0.953736017	0.201006741	2.246346116
DQ579704	NULL	0.625465585	0.230432138	1.440588564
DQ580013	NULL	0.119400972	0.300341265	-1.330788122
DQ580039	NULL	0.364853372	0.146835726	1.31311376
DQ580140	NULL	0.402380457	1.109952714	-1.463866073
DQ580247	NULL	0.085194694	0.431513048	-2.340568696
DQ580250	NULL	0.215556517	0.654423764	-1.60215896
DQ581337	NULL	0.084312114	0.410013052	-2.281857998
DQ581862	NULL	0.062542055	0.456406408	-2.867420518
DQ581988	NULL	0.290900119	1.029114742	-1.822808054
DQ582763	NULL	0.068367906	0.197752186	-1.532302499
DQ582871	NULL	0.119962359	0.43319019	-1.852418781
DQ582989	NULL	0.358140397	0.922450958	-1.364946954
DQ583079	NULL	0.422693043	0.202749178	1.059914301
DQ583119	NULL	1.027197	0.406441342	1.337593833
DQ583233	NULL	0.078374834	0.222071983	-1.502565004
DQ583509	NULL	0.928093712	0.381991206	1.280731062
DQ583692	NULL	0.126336625	0.417265383	-1.7236923

DQ583793	NULL	1.705813597	0.674871928	1.337774356
DQ584116	NULL	0.648786837	0.261630385	1.310214447
DQ584254	NULL	0.470329429	0.157611768	1.577296352
DQ584258	NULL	0.684816119	0.243923912	1.489285468
DQ584667	NULL	0.334119608	0.105248309	1.666567594
DQ584699	NULL	0.237513701	0.828599521	-1.802664248
DQ584913	NULL	0.206213591	0.431712674	-1.065932026
DQ584937	NULL	0.382063374	0.145236764	1.395405276
DQ584943	NULL	0.10303334	0.212395339	-1.04364086
DQ584993	NULL	0.178474944	0.901844698	-2.337157466
DQ585001	NULL	0.097385775	0.271454638	-1.478928178
DQ585433	NULL	0.09580337	0.249576814	-1.381335606
DQ585964	NULL	0.303840039	0.656617719	-1.111741686
DQ586086	NULL	0.241110723	0.105478616	1.192745274
DQ586241	NULL	0.112456074	0.369937665	-1.717920613
DQ586243	NULL	0.417170078	0.155104579	1.427394409
DQ586951	NULL	3.411489615	7.775184662	-1.188475115
DQ587139	NULL	0.094408192	0.338583168	-1.842526296
DQ587495	NULL	0.169310767	0.786660394	-2.216067232
DQ587808	NULL	0.076368969	0.162191454	-1.086639362
DQ587954	NULL	0.149127405	0.799743342	-2.422991676
DQ587955	NULL	0.234246341	0.479705141	-1.034121387
DQ588075	NULL	0.034995217	0.297643298	-3.088354771
DQ588120	NULL	0.094871767	0.340720675	-1.844538768
DQ588170	NULL	0.144714473	0.372653401	-1.364625215
DQ588370	NULL	0.124571567	0.456167431	-1.87258863
DQ588394	NULL	0.133687009	0.311343186	-1.219646429
DQ588542	NULL	0.14298517	0.510308104	-1.835503034
DQ588654	NULL	0.350946286	1.112144045	-1.664021518
DQ588677	NULL	0.554833271	1.134481437	-1.031906797
DQ588877	NULL	0.198689326	0.568428856	-1.516465424
DQ589171	NULL	0.087383292	0.23066671	-1.400380445
DQ589342	NULL	0.170558808	0.462581048	-1.439436902
DQ589586	NULL	1.118745418	0.474447477	1.237561485
DQ589665	NULL	0.237491287	0.658621265	-1.471574508
DQ589981	NULL	0.179665328	0.065099063	1.46460334
DQ590166	NULL	3.49193279	1.593294632	1.132012717
DQ590295	NULL	0.027881157	0.080046375	-1.521545656
DQ590919	NULL	0.192039187	0.470332654	-1.292280769
DQ591298	NULL	0.210358902	0.700570304	-1.735676967
DQ591313	NULL	0.224618238	0.089484077	1.327772179
DQ592298	NULL	0.077969207	0.259318604	-1.733749354
DQ592342	NULL	0.279792155	0.611793567	-1.128689427
DQ592463	NULL	0.086451666	0.184203997	-1.091338695
DQ593444	NULL	0.161612746	0.407431882	-1.334017897
DQ593488	NULL	0.096895459	0.272749495	-1.49307557
DQ593531	NULL	0.361198235	0.096523277	1.903842049
DQ593624	NULL	0.209061854	0.465822384	-1.155850117
DQ593864	NULL	0.161010763	0.534332401	-1.730580375
DQ593900	NULL	0.077273715	0.495169544	-2.679872927
DQ594177	NULL	0.066678566	0.53257667	-2.997694248
DQ594400	NULL	0.154213448	0.36732219	-1.252117478
DQ594620	NULL	0.363418229	0.175274794	1.052012247
DQ594674	NULL	0.052557432	0.106338954	-1.016703483
DQ595116	NULL	0.075138755	0.188831603	-1.329471124
DQ595165	NULL	0.136067294	0.309674647	-1.186432942
DQ595182	NULL	0.551882354	1.21757654	-1.141579805
DQ595216	NULL	0.618279969	1.423763742	-1.203377597
DQ595361	NULL	0.391054187	0.193094201	1.018063694
DQ595383	NULL	0.061262781	0.16312566	-1.412900982
DQ595853	NULL	0.069085573	0.454872612	-2.7190062
DQ596025	NULL	0.298442349	0.865718594	-1.53644588
DQ596074	NULL	0.553767814	0.26203438	1.079525094
DQ596486	NULL	0.125868615	0.312477312	-1.311832846
DQ596518	NULL	0.087490267	0.23380388	-1.418104444
DQ596562	NULL	0.129751118	0.287288448	-1.14675301
DQ596761	NULL	0.088564507	0.247534566	-1.48282946
DQ596791	NULL	0.139526001	0.317374404	-1.185651787
DQ596932	NULL	0.206797435	0.851040324	-2.041009199
DQ596958	NULL	0.989846732	2.020491639	-1.029429321

DQ597036	NULL	0.340034448	0.164107709	1.051037902
DQ597117	NULL	0.666732038	1.379490667	-1.048956737
DQ597620	NULL	0.428033086	0.080750314	2.406182543
DQ597730	NULL	0.332554938	0.145945021	1.188167705
DQ597986	NULL	0.067668114	0.207359335	-1.615584909
DQ598339	NULL	0.178764905	0.390863826	-1.128602534
DQ598479	NULL	0.646321687	1.473423563	-1.188847914
DQ599043	NULL	1.333539537	0.334711428	1.994270884
DQ599224	NULL	0.068862707	0.240356532	-1.803381222
DQ599251	NULL	0.282136954	0.116384931	1.277491364
DQ599837	NULL	0.850462408	0.414093175	1.038292042
DQ599995	NULL	0.50257	0.16138328	1.638833436
DQ600033	NULL	0.165829508	0.624485764	-1.912967937
DQ600156	NULL	0.094568656	0.382542893	-2.016187528
DQ600292	NULL	0.180935562	0.40272642	-1.15432413
DQ600701	NULL	0.023334591	0.052650226	-1.17396953
DQ601415	NULL	0.259475285	1.314021587	-2.340319943
DQ656011	NULL	0.021716546	0.052997819	-1.287138348
DQ786325	NULL	0.235672717	0.557374669	-1.241862684
EIF1AD	eukaryotic translation initiation factor 1A domain containing	0.659432393	1.439278694	-1.126049313
ELTD1	NULL	0.035460362	0.102255955	-1.527905714
Evf1_2	NULL	0.199736899	0.084660953	1.238332253
EXO5	exonuclease 5	0.430315065	0.932393836	-1.11554612
FAM127A	NULL	0.232164905	0.071912273	1.690839999
FGF7	fibroblast growth factor 7	0.054185141	0.11473826	-1.082377372
FLJ44955	NULL	0.051226185	0.132798004	-1.374280104
FW312330	NULL	0.307664795	0.110883946	1.47230887
FW420894	NULL	0.148717193	0.329575405	-1.148037138
FZD7	frizzled class receptor 7	0.104470067	0.239966954	-1.199746115
GPATCH3	G-patch domain containing 3	0.321340434	0.652904869	-1.022770275
GPR171	G protein-coupled receptor 171	0.113575036	0.2741921	-1.27154124
GPR174	G protein-coupled receptor 174	0.011223785	0.022529609	-1.005262965
GPR22	G protein-coupled receptor 22	0.160366132	0.068244957	1.232575148
GPR34	G protein-coupled receptor 34	0.112421056	0.342504254	-1.607209642
GPR52	G protein-coupled receptor 52	0.241676926	0.082714322	1.546870686
GPX1	glutathione peroxidase 1	0.256667055	0.538512076	-1.069080569
hADV14S1	NULL	0.064645938	0.155200226	-1.263499038
Hel-N1	NULL	0.226695489	0.06409539	1.82246319
HIST1H2BB	NULL	0.442078213	0.214068238	1.046230881
HIST1H2BE	NULL	0.94761682	0.466278544	1.023111762
HIST1H2BM	NULL	0.241743753	0.720783078	-1.576086532
HIST1H4I	NULL	0.492905993	0.202466707	1.283627829
HIST1H4J	NULL	0.267694865	0.565143772	-1.078028469
HIST2H2BC	NULL	0.191936522	0.466830412	-1.282269297
HM358988	NULL	0.03419733	0.203644208	-2.574095188
HP07349	NULL	0.348450104	0.126057229	1.466873236
hsa-miR-3074-5p	NULL	0.188187948	0.615269509	-1.709044264
HSP90AB4P	heat shock protein 90 alpha family class B member 4, pseudogene	0.10652939	0.218467084	-1.03616442
HUS1B	HUS1 checkpoint clamp component B	0.119454423	0.245748894	-1.040724648
HV303147	NULL	0.364309738	0.136516033	1.416095165
HV303525	NULL	0.023343498	0.055661389	-1.253656147
HV303528	NULL	0.023116168	0.098135232	-2.085868911
HV530979	NULL	0.403860193	0.14113929	1.516736293
HV531014	NULL	0.123992052	0.358670516	-1.532411508
HV531015	NULL	0.403860193	0.14113929	1.516736293
HV531016	NULL	0.166096689	0.368764327	-1.150675791
HV531022	NULL	0.162120674	0.331569857	-1.032244784
HV592900	NULL	0.509978211	0.192564216	1.405095979
HV592934	NULL	0.085203615	0.504420446	-2.565640216
HV592964	NULL	0.166444646	0.366431103	-1.138499504
HV592965	NULL	0.679573467	0.200581022	1.760444414
HV593178	NULL	0.433689353	0.924714006	-1.092345216
HV745902	NULL	0.116525768	0.040062306	1.540331651
HV781757	NULL	1.222711195	0.602355835	1.021395778
HV941429	NULL	0.496031073	0.155108916	1.677148881
HV941430	NULL	0.358283021	0.122142281	1.552536989
HV941440	NULL	0.495393661	0.123193789	2.007645892
HV941478	NULL	0.51200193	0.114320993	2.1630589
ID1	inhibitor of DNA binding 1	0.332006419	0.69647595	-1.068862401
IGHV 3-07	NULL	0.145347551	0.360717387	-1.311362204

IL10RB-AS1	NULL	0.171271891	0.375912308	-1.13410776
IL8	NULL	0.037460134	0.076316802	-1.026644667
immunoglobulin heavy chain	NULL	0.029815678	0.118709534	-1.993292753
INE1	inactivation escape 1	0.097243099	0.211230884	-1.11915301
IRAK4	interleukin 1 receptor associated kinase 4	0.561221196	1.138185269	-1.020094011
IRES_Cx43	NULL	0.298785603	0.014000079	4.41560376
ISM1-AS1	ISM1 antisense RNA 1	0.075843026	0.186323057	-1.296717793
JA072123	NULL	0.027101124	0.056178476	-1.051664787
JA202257	NULL	0.009164454	0.040996525	-2.161380853
JA202295	NULL	0.302396149	0.069231279	2.126943871
JA202330	NULL	0.006726254	0.025805862	-1.939823629
JA375049	NULL	0.037482454	0.206242559	-2.460054773
JA425978	NULL	0.635374464	1.309341631	-1.043162559
JA429818	NULL	0.024939249	0.113669144	-2.188350783
JA429839	NULL	0.700965517	0.202401646	1.792122455
JA429845	NULL	0.064325055	0.202992413	-1.657973112
JA429848	NULL	0.33375856	0.090283574	1.886269403
JA611242	NULL	0.391967831	0.068766396	2.510959623
JA611268	NULL	0.233127426	0.538951202	-1.209035914
JA611269	NULL	0.125320469	0.351992354	-1.489922017
JA611275	NULL	0.148311579	0.634641652	-2.097310973
JA611291	NULL	0.027685028	0.11933957	-2.107894618
JA660634	NULL	0.044908499	0.145588633	-1.696837323
JA700151	NULL	0.321616486	0.719709556	-1.162073455
JA700158	NULL	0.321099422	0.731719195	-1.188270036
JA700163	NULL	0.321687806	0.667433215	-1.052962237
JA700164	NULL	0.191798192	0.442571521	-1.206321496
JA700167	NULL	0.222438779	0.830841297	-1.901164604
JA700174	NULL	0.486327264	0.204513317	1.249732688
JA783409	NULL	0.01401413	0.058459679	-2.060559711
JA783425	NULL	0.016216102	0.040892232	-1.334399787
JA783430	NULL	0.024689645	0.156195119	-2.661371368
JA783435	NULL	0.044083916	0.215940936	-2.292312468
JA783482	NULL	0.019929128	0.083935275	-2.074398693
JA783498	NULL	0.020860727	0.042087151	-1.01259045
JA783500	NULL	0.019897924	0.042087153	-1.080761986
JA783507	NULL	0.009164453	0.211048311	-4.525380697
JA783513	NULL	0.007194264	0.044498707	-2.628844494
JA783533	NULL	0.029013347	0.009937365	1.545781547
JA783536	NULL	0.028478458	0.009937365	1.518935745
JA783574	NULL	0.010711185	0.027596059	-1.365344194
JA783579	NULL	0.006726255	0.025697233	-1.933737723
JA783589	NULL	0.187947321	0.007769129	4.596431675
JA783645	NULL	0.039466022	0.017006917	1.214489457
JA783654	NULL	0.039234235	0.017006917	1.205991436
JA783679	NULL	0.024582651	0.055900402	-1.185218183
JA783690	NULL	0.052000281	0.141460733	-1.443810318
JA783775	NULL	0.006681668	0.073315724	-3.455842431
JA783787	NULL	0.02444449	0.155869233	-2.672754967
JA783844	NULL	0.025313697	0.102058889	-2.011411734
JA783854	NULL	0.030105433	0.10883298	-1.854020059
JB023059	NULL	0.128173091	0.291290244	-1.184363973
JB074828	NULL	0.039220855	0.18646345	-2.249199993
JB074934	NULL	0.091586645	0.321463056	-1.811443806
JB153694	NULL	0.014245902	0.043560153	-1.612462034
JB155089	NULL	0.099235581	0.37511739	-1.918412748
JB158051	NULL	0.08913951	0.198382268	-1.154146148
JB175177	NULL	0.527415441	1.096324656	-1.055663376
JB175300	NULL	0.013782341	0.042721552	-1.632143153
JX003871	NULL	0.072165634	0.184018728	-1.35046872
KIF18A	kinesin family member 18A	0.118399417	0.240794443	-1.024140127
KRTAP1-3	keratin associated protein 1-3	0.047498214	0.211091991	-2.151926682
KRTAP1-5	keratin associated protein 1-5	0.043491053	0.207111706	-2.251618549
KRTAP19-1	keratin associated protein 19-1	0.105694288	0.212525747	-1.007740226
KRTAP19-6	keratin associated protein 19-6	0.162053999	0.056030735	1.532184313
KRTAP19-8	keratin associated protein 19-8	0.316690913	0.673694522	-1.089019099
KRTAP2-3	keratin associated protein 2-3	0.077113359	0.191312725	-1.310880119
KRTAP20-1	keratin associated protein 20-1	0.103166977	0.035534658	1.537682549
KRTAP20-4	keratin associated protein 20-4	0.026133845	0.085030299	-1.702057611
KRTAP21-3	keratin associated protein 21-3	0.317497882	0.153192786	1.051398612

KRTAP4-12	keratin associated protein 4-12	0.060616441	0.150233661	-1.309427049
KRTAP4-3	keratin associated protein 4-3	0.24288048	0.117001948	1.053713997
KRTAP4-4	keratin associated protein 4-4	0.258677524	0.128790321	1.006130538
KRTAP4-6	keratin associated protein 4-6	0.077590355	0.213759613	-1.462040069
KRTAP4-7	keratin associated protein 4-7	0.072704857	0.232947982	-1.679884192
KRTAP7-1	keratin associated protein 7-1	0.071171599	0.164577045	-1.209389576
KRTAP8-1	keratin associated protein 8-1	0.081249812	0.371884189	-2.194417041
L37717	NULL	0.330410854	0.935225217	-1.501052751
LAGE3	L antigen family member 3	0.06368909	0.140893113	-1.145482941
LAPTM4A	lysosomal protein transmembrane 4 alpha	0.163401083	0.358838084	-1.134915466
LDB2	LIM domain binding 2	0.466032866	0.948463357	-1.025160338
LINC00410	long intergenic non-protein coding RNA 410	0.038789757	0.088623836	-1.192019036
LINC00424	long intergenic non-protein coding RNA 424	0.06697866	0.144130188	-1.105599121
LINC00889	NULL	0.016217569	0.036925094	-1.187044042
LINC00906	long intergenic non-protein coding RNA 906	0.122414123	0.29896615	-1.288212138
LOC100128398	NULL	0.152684459	0.445117471	-1.543632903
LOC100130700	NULL	0.075152125	0.22566984	-1.586327817
LOC100133957	NULL	0.577846799	0.276695008	1.062390433
LOC100133985	NULL	0.059961214	0.137876016	-1.201270031
LOC100288842	NULL	0.085282342	0.185947961	-1.124579966
LOC100289673	NULL	0.145631358	0.331459938	-1.186513473
LOC286238	NULL	0.089570379	0.216733387	-1.274827797
LOC286367	NULL	0.073891373	0.153644662	-1.056119814
LOC392452	NULL	0.009454187	0.043056099	-2.187192384
LOC441666	zinc finger protein 91 pseudogene	0.046619323	0.094982143	-1.026728261
LOC541472	NULL	0.13319366	0.278502621	-1.064165493
LOC606724	coronin 1A pseudogene	0.389498469	0.856777454	-1.137302858
LOC644189	acyl-CoA thioesterase 4 pseudogene	0.096262397	0.217070805	-1.173121447
LOC645434	NULL	0.048248598	0.123109744	-1.351386028
LOC646903	uncharacterized LOC646903	0.082497966	0.166762608	-1.015365385
LOC727896	NULL	0.057202038	0.135343575	-1.242487961
LSP1P3	LSP1 pseudogene 3	0.074519161	0.268143845	-1.847323799
LUM	lumican	0.034810601	0.082476219	-1.244451465
LUST	NULL	0.184149527	0.06589859	1.482558197
LYPD8	LY6/PLAUR domain containing 8	0.110954061	0.350858203	-1.660925621
MAGEB17	MAGE family member B17	0.061218938	0.140185483	-1.195287047
MAGEB5	MAGE family member B5	0.028857302	0.080934242	-1.487813791
MAGEB6	MAGE family member B6	0.095241725	0.196774673	-1.046878894
MAGEC2	MAGE family member C2	0.082849975	0.180667113	-1.124760748
MAS1L	MAS1 proto-oncogene like, G protein-coupled receptor	0.109541056	0.340881658	-1.637799287
Mir_1183	NULL	0.056181315	0.172150531	-1.615508329
Mir_147	NULL	0.141144008	0.307801765	-1.124833621
Mir_1827	NULL	0.141621244	0.295579115	-1.061506646
Mir_361	NULL	0.010760215	0.025788467	-1.261019082
Mir_500	NULL	0.266620691	0.112617613	1.243356267
Mir_576	NULL	0.025870868	0.059789272	-1.208558186
Mir_887	NULL	0.050663021	0.190017801	-1.907129576
mir-108-1	NULL	0.235191188	0.801081523	-1.768115063
mir-220	NULL	0.085840979	0.030694161	1.483702286
MIR100	microRNA 100	0.177244904	0.085855842	1.045755943
MIR101-2	microRNA 101-2	0.0630502	0.174696795	-1.470280285
MIR103A1	microRNA 103a-1	0.115206589	0.035864886	1.683579281
MIR103A2	microRNA 103a-2	0.227703043	0.100651113	1.17779045
MIR103B1	microRNA 103b-1	0.115313566	0.035864887	1.684918278
MIR103B2	microRNA 103b-2	0.23113526	0.099747319	1.212387388
MIR105-1	microRNA 105-1	0.029993974	0.135403579	-2.17452128
MIR105-2	microRNA 105-2	0.028295696	0.072924663	-1.36582418
MIR10A	microRNA 10a	0.620887641	0.227234142	1.4501526
MIR1182	microRNA 1182	0.089785846	0.262951139	-1.550234812
MIR1185-1	microRNA 1185-1	0.805306029	0.232452588	1.792600646
MIR1207	microRNA 1207	0.042260802	0.158771839	-1.909563009
MIR1208	microRNA 1208	0.218364741	0.106660513	1.033713757
MIR1231	microRNA 1231	0.104874158	0.252149189	-1.265618361
MIR1233-2	microRNA 1233-2	0.890031345	0.440828802	1.01363766
MIR1236	microRNA 1236	0.301910121	0.150933242	1.000208539
MIR1238	microRNA 1238	0.113918356	0.253417932	-1.153518389
MIR1245A	microRNA 1245a	0.165259006	0.012679148	3.704199186
MIR1245B	microRNA 1245b	0.167193528	0.012670458	3.721978423
MIR1251	microRNA 1251	0.024600489	0.066876233	-1.442806622
MIR125B2	microRNA 125b-2	0.036207615	0.011749287	1.623719939

MIR1264	microRNA 1264	0.09841546	0.015642546	2.653409619
MIR1277	microRNA 1277	0.034188419	0.009355109	1.86968132
MIR1278	microRNA 1278	0.175461829	0.046297606	1.922147723
MIR1279	microRNA 1279	0.04545232	0.103384192	-1.18558976
MIR1283-1	microRNA 1283-1	0.155786749	0.069209601	1.170528438
MIR1283-2	microRNA 1283-2	0.095018836	0.337674963	-1.829349774
MIR1284	microRNA 1284	0.045875767	0.239348312	-2.38330746
MIR1287	microRNA 1287	0.370786349	0.12789958	1.535576609
MIR1288	microRNA 1288	0.083732666	0.175357341	-1.066435371
MIR1295B	microRNA 1295b	0.123737831	0.060988557	1.020676157
MIR1296	microRNA 1296	0.053025476	0.220816201	-2.058088453
MIR1304	microRNA 1304	0.049303543	0.164077266	-1.734612143
MIR1306	microRNA 1306	0.29844233	0.122429016	1.285506653
MIR130B	microRNA 130b	0.94310202	0.430361479	1.131864893
MIR1322	microRNA 1322	0.124794215	0.341333535	-1.451631102
MIR133A1	microRNA 133a-1	0.028358122	0.066311344	-1.225493702
MIR1343	microRNA 1343	0.02714123	0.441624153	-4.024261094
MIR136	microRNA 136	0.151605848	0.3332429	-1.136248738
MIR141	microRNA 141	0.172716178	0.645095045	-1.901108515
MIR1469	microRNA 1469	0.268764828	0.720227722	-1.422108782
MIR146A	microRNA 146a	0.87642736	0.309083718	1.503636867
MIR146B	microRNA 146b	0.660870963	1.393074415	-1.075831812
MIR147B	microRNA 147b	0.354984545	0.15409218	1.203962571
MIR1537	microRNA 1537	0.185869886	0.044190197	2.072494797
MIR1538	microRNA 1538	0.066896983	0.336345584	-2.329931264
MIR155	microRNA 155	0.023503968	0.133139946	-2.501967246
MIR181A1	microRNA 181a-1	0.043936809	0.40604611	-3.208141582
MIR181A2	microRNA 181a-2	0.113000044	0.231248952	-1.03312349
MIR181B2	microRNA 181b-2	0.406904907	0.059172296	2.781697898
MIR1827	microRNA 1827	0.128039328	0.687038515	-2.423803973
MIR1912	microRNA 1912	0.026013506	0.108511442	-2.060514417
MIR1914	microRNA 1914	0.201764984	0.560659779	-1.474449756
MIR192	microRNA 192	0.409449905	0.954126243	-1.220493212
MIR197	microRNA 197	0.014031954	0.418594885	-4.898766854
MIR1972-1	microRNA 1972-1	0.13439575	0.050321199	1.417249323
MIR2052	microRNA 2052	0.7949769	0.307719436	1.369297372
MIR2053	microRNA 2053	0.123492929	0.037507346	1.719183345
MIR2054	microRNA 2054	0.118576345	0.043564502	1.444591293
MIR2113	microRNA 2113	0.063981823	0.221076944	-1.788814571
MIR2114	microRNA 2114	0.071465823	0.171776829	-1.265210066
MIR215	microRNA 215	0.034602955	0.334477314	-3.272941231
MIR216B	microRNA 216b	0.145976027	0.038589295	1.919458864
MIR218-1	microRNA 218-1	0.068831346	0.020895839	1.71985007
MIR22	microRNA 22	0.177592597	0.471340616	-1.408198562
MIR2278	microRNA 2278	0.102921895	0.421749705	-2.034837134
MIR2467	microRNA 2467	0.181626455	0.063408828	1.51821875
MIR25	microRNA 25	0.416225318	0.856074062	-1.040370892
MIR2681	microRNA 2681	0.15373195	0.526272793	-1.775393787
MIR26B	microRNA 26b	0.331574121	0.754509182	-1.186207049
MIR2909	microRNA 2909	0.296739512	0.123237161	1.267759691
MIR29A	microRNA 29a	0.088649188	0.489264495	-2.46443527
MIR29B1	microRNA 29b-1	0.293623625	0.11436881	1.36027439
MIR302F	microRNA 302f	0.086687952	0.250011314	-1.528089987
MIR3064	microRNA 3064	0.209418462	0.09864799	1.086027073
MIR30B	microRNA 30b	0.085765284	0.033205649	1.36896511
MIR30C1	microRNA 30c-1	0.107566508	0.276629742	-1.362727332
MIR30E	microRNA 30e	0.054425099	0.155695455	-1.516382799
MIR31	microRNA 31	0.100229387	0.213455528	-1.090629968
MIR3115	microRNA 3115	0.032864565	0.124067101	-1.916515805
MIR3117	microRNA 3117	0.040887917	0.165815259	-2.01983032
MIR3119-1	microRNA 3119-1	0.042131517	0.11926137	-1.501155047
MIR3119-2	microRNA 3119-2	0.042131517	0.11926137	-1.501155047
MIR3120	microRNA 3120	0.075116486	0.298720633	-1.99159542
MIR3124	microRNA 3124	0.206721957	0.44164604	-1.095198941
MIR3125	microRNA 3125	0.079507023	0.169891028	-1.095455458
MIR3126	microRNA 3126	0.086848349	0.338904657	-1.964309141
MIR3129	microRNA 3129	0.028277864	0.086151301	-1.607199426
MIR3145	microRNA 3145	0.036902999	0.078716805	-1.09293362
MIR3147	microRNA 3147	0.375867605	0.039006416	3.268441244
MIR3152	microRNA 3152	0.025665841	0.09179569	-1.838576911

MIR3156-2	microRNA 3156-2	0.089259926	0.183108976	-1.036618002
MIR3157	microRNA 3157	0.093975596	0.198073712	-1.07567936
MIR3162	microRNA 3162	0.093258208	0.239674151	-1.361771714
MIR3169	microRNA 3169	0.005580682	0.228967705	-5.358558875
MIR3175	microRNA 3175	0.477184996	0.223232155	1.096003831
MIR3177	microRNA 3177	0.15660247	0.501365746	-1.67875647
MIR3178	microRNA 3178	0.22147145	0.614006179	-1.471132445
MIR3184	microRNA 3184	0.456079172	0.169578208	1.427333505
MIR3188	microRNA 3188	0.092883733	0.266892308	-1.522759871
MIR3193	microRNA 3193	0.63570867	0.232617713	1.450404806
MIR3198-1	microRNA 3198-1	0.079743183	0.347516665	-2.123649075
MIR3200	microRNA 3200	0.071051217	0.357341179	-2.330370918
MIR320C2	microRNA 320c-2	0.094292131	0.263129433	-1.480563355
MIR323A	microRNA 323a	0.441645896	0.215406614	1.035827561
MIR323B	microRNA 323b	0.35752088	0.716516938	-1.002973308
MIR326	microRNA 326	0.209556735	0.453768868	-1.114616745
MIR331	microRNA 331	0.114564679	0.045432912	1.334352648
MIR34B	microRNA 34b	0.53813148	1.090233707	-1.018606821
MIR3605	microRNA 3605	0.107254451	0.357267253	-1.735966163
MIR3606	microRNA 3606	0.024426665	0.266496795	-3.4475894
MIR3609	microRNA 3609	0.105498255	0.217444422	-1.043427571
MIR3612	microRNA 3612	0.091956363	0.209675245	-1.189135234
MIR3613	microRNA 3613	0.045309666	0.833238782	-4.200839227
MIR362	microRNA 362	0.421676567	0.043642703	3.272324504
MIR3622A	microRNA 3622a	0.089763561	0.229741246	-1.35580808
MIR3622B	microRNA 3622b	0.096173337	0.233252128	-1.278181367
MIR3656	NULL	0.093088807	0.241255903	-1.373884629
MIR365A	microRNA 365a	0.089990707	0.668289256	-2.892624747
MIR3662	microRNA 3662	0.163631788	0.017237218	3.246854229
MIR3679	microRNA 3679	0.287418923	0.106121683	1.437435588
MIR3682	microRNA 3682	0.040402056	0.106864711	-1.403284907
MIR3685	microRNA 3685	0.059484275	0.135164604	-1.184137153
MIR3691	microRNA 3691	0.051010697	0.312855436	-2.616624451
MIR3692	microRNA 3692	0.914542937	1.973611778	-1.10971542
MIR374A	microRNA 374a	0.117047497	0.035534656	1.719795439
MIR376A1	microRNA 376a-1	0.342691212	0.104579151	1.71231393
MIR378D2	microRNA 378d-2	0.066223913	0.266462173	-2.008506587
MIR3909	microRNA 3909	0.297180841	0.678799718	-1.191644858
MIR3912	microRNA 3912	0.28560498	0.611103165	-1.097394818
MIR3916	microRNA 3916	0.116485699	0.052641525	1.145879664
MIR3919	microRNA 3919	0.45007056	0.188683936	1.254179601
MIR3924	microRNA 3924	0.107874124	0.033966043	1.667183797
MIR3936	microRNA 3936	0.819413262	0.270455267	1.599201251
MIR3938	microRNA 3938	0.558234472	0.147817801	1.917051204
MIR3941	microRNA 3941	0.032592634	0.140057243	-2.103398739
MIR3942	microRNA 3942	0.013595127	0.111122889	-3.030994532
MIR3960	microRNA 3960	0.237366555	0.504025012	-1.086378658
MIR3973	microRNA 3973	0.078793851	0.328919471	-2.061579461
MIR3975	microRNA 3975	0.346332723	0.053580086	2.692389904
MIR421	microRNA 421	0.102150766	0.036642684	1.479102948
MIR423	microRNA 423	0.431634645	0.168822153	1.354306441
MIR424	microRNA 424	0.154971293	0.053210719	1.5422122
MIR4254	microRNA 4254	0.189132708	0.707169531	-1.902657235
MIR4255	microRNA 4255	0.054398324	0.163842658	-1.590676926
MIR4256	microRNA 4256	0.102137366	0.334246762	-1.710402825
MIR4257	microRNA 4257	0.177949196	0.51444893	-1.531562454
MIR4263	microRNA 4263	0.040134589	0.117579802	-1.550722219
MIR4264	microRNA 4264	0.089197493	0.209649173	-1.232902077
MIR4272	microRNA 4272	0.432945055	0.177964425	1.282595071
MIR4273	microRNA 4273	0.163261767	0.360000419	-1.140811607
MIR4275	microRNA 4275	0.02600013	0.122433304	-2.235405302
MIR4278	microRNA 4278	0.938082165	0.439164694	1.094952215
MIR4282	microRNA 4282	0.029205013	0.065029531	-1.154879006
MIR4284	microRNA 4284	0.785741458	0.157968081	2.314421606
MIR4295	microRNA 4295	0.150496004	0.051659539	1.54261852
MIR4303	microRNA 4303	0.038213464	0.267683182	-2.808373553
MIR4305	microRNA 4305	0.030658145	0.148834429	-2.279365982
MIR4322	microRNA 4322	0.09427893	0.384046559	-2.026273931
MIR4324	microRNA 4324	0.217410749	0.507366344	-1.222604551
MIR4329	microRNA 4329	0.024738677	0.071425585	-1.529672628

MIR4422	microRNA 4422	0.62345489	0.122281309	2.350081275
MIR4423	microRNA 4423	0.063246359	0.167149312	-1.402083079
MIR4427	microRNA 4427	0.089175012	0.232217879	-1.38076764
MIR4450	microRNA 4450	0.067307061	0.13691139	-1.024412701
MIR4451	microRNA 4451	0.033354872	0.163421158	-2.292625387
MIR4465	microRNA 4465	0.018961868	0.25008985	-3.72127354
MIR4467	microRNA 4467	0.212195613	0.869474476	-2.03474885
MIR4468	microRNA 4468	0.409258216	0.827204734	-1.015233064
MIR4469	microRNA 4469	0.333058544	0.08032884	2.051785842
MIR4473	microRNA 4473	0.137471379	0.280523213	-1.028988871
MIR4474	microRNA 4474	0.054915426	0.151241678	-1.461572398
MIR448	microRNA 448	0.011446657	0.022916347	-1.001450737
MIR4480	microRNA 4480	0.058900357	0.213438139	-1.857469721
MIR4484	microRNA 4484	0.12041726	0.627502012	-2.381577894
MIR4489	microRNA 4489	0.454184765	0.197196056	1.203648616
MIR4500	microRNA 4500	0.012806167	0.126039771	-3.298968461
MIR4505	microRNA 4505	0.096913233	0.212829889	-1.134935199
MIR450A2	microRNA 450a-2	0.032231643	0.216866647	-2.750258573
MIR4510	microRNA 4510	0.037950483	0.129359493	-1.769195777
MIR4511	microRNA 4511	0.052339007	0.105478547	-1.010991145
MIR451A	microRNA 451a	0.091546533	0.19236858	-1.071296024
MIR451B	microRNA 451b	0.091546529	0.194601986	-1.087949349
MIR452	microRNA 452	0.03877956	0.103040918	-1.409849012
MIR4524A	microRNA 4524a	0.264615028	0.095536851	1.469765775
MIR4524B	microRNA 4524b	0.225639302	0.107147093	1.074425677
MIR4531	microRNA 4531	0.243437482	0.753067333	-1.629227548
MIR4535	microRNA 4535	0.1608191	0.407006651	-1.339613607
MIR454	microRNA 454	0.056176895	0.322506011	-2.521277272
MIR455	microRNA 455	0.527839193	0.209705804	1.331731686
MIR4639	microRNA 4639	0.508908336	0.157772492	1.689560133
MIR4642	microRNA 4642	0.154030725	0.060919035	1.338253177
MIR4643	microRNA 4643	0.083875301	0.561489551	-2.74294123
MIR4648	microRNA 4648	0.354628022	0.755815049	-1.091726704
MIR4651	microRNA 4651	0.761124008	0.359839619	1.080777488
MIR4658	microRNA 4658	0.133896504	1.15242514	-3.105482846
MIR4663	microRNA 4663	0.354396355	0.909288356	-1.359374113
MIR4668	microRNA 4668	0.025523191	0.051524828	-1.013459087
MIR4670	microRNA 4670	0.046080813	0.28240439	-2.615524456
MIR4675	microRNA 4675	0.130562219	0.265180212	-1.022235647
MIR4676	microRNA 4676	0.045617241	0.127647498	-1.484514162
MIR4679-1	microRNA 4679-1	0.020548718	0.049369635	-1.264575575
MIR4679-2	microRNA 4679-2	0.020548718	0.049369633	-1.264575566
MIR4681	microRNA 4681	0.129059991	0.314093898	-1.283154089
MIR4685	microRNA 4685	0.108337649	0.242181361	-1.16055315
MIR4687	microRNA 4687	0.275923336	0.930357461	-1.753517657
MIR4688	microRNA 4688	0.850967677	0.361577543	1.234799259
MIR4694	microRNA 4694	0.147692233	0.467382241	-1.662008958
MIR4699	microRNA 4699	0.063696391	0.022659964	1.491066052
MIR4703	microRNA 4703	0.174240469	0.37281433	-1.097377572
MIR4708	microRNA 4708	0.054817348	0.158724044	-1.533816245
MIR4709	microRNA 4709	0.316766577	0.137233009	1.206792587
MIR4718	microRNA 4718	0.09497873	0.328771783	-1.791410114
MIR4721	microRNA 4721	0.518206653	0.255056058	1.022713172
MIR4722	microRNA 4722	0.31652609	0.927559848	-1.551115952
MIR4723	microRNA 4723	0.327700752	0.128373233	1.352034571
MIR4724	microRNA 4724	0.747688583	0.1597322	2.226782332
MIR4725	microRNA 4725	0.1686999	0.940339324	-2.478722337
MIR4731	microRNA 4731	0.298170288	0.08908437	1.742892276
MIR4734	microRNA 4734	0.446651578	0.221420332	1.012362158
MIR4735	microRNA 4735	0.266174921	0.109571676	1.280499737
MIR4738	microRNA 4738	0.074202699	0.392897698	-2.404610154
MIR4740	microRNA 4740	0.120644333	0.405828899	-1.750111459
MIR4742	microRNA 4742	0.101999227	0.04079229	1.322189814
MIR4745	microRNA 4745	0.16103761	0.448081046	-1.47636204
MIR4753	microRNA 4753	0.146662625	0.032966668	2.153421305
MIR4762	microRNA 4762	0.056159034	0.160153602	-1.511866217
MIR4764	microRNA 4764	0.294501953	0.651225803	-1.144880661
MIR4765	microRNA 4765	0.048033176	0.297165197	-2.629162065
MIR4767	microRNA 4767	0.081129458	0.180536679	-1.153994224
MIR4769	microRNA 4769	0.223414871	0.062179133	1.845222821

MIR4773-1	microRNA 4773-1	0.03915843	0.108954658	-1.476333047
MIR4773-2	microRNA 4773-2	0.03915843	0.108954658	-1.476333047
MIR4777	microRNA 4777	0.382232851	0.105091874	1.862800663
MIR4782	microRNA 4782	0.020976619	0.053914658	-1.361895381
MIR4789	microRNA 4789	0.127887806	0.259179729	-1.019074181
MIR4791	microRNA 4791	0.00591499	0.019205573	-1.699077457
MIR4796	microRNA 4796	0.127994761	0.033318626	1.941683969
MIR4804	microRNA 4804	0.05741605	0.288400934	-2.328549862
MIR484	microRNA 484	0.045710844	0.126661163	-1.470365869
MIR491	microRNA 491	0.146042908	0.066311406	1.139063351
MIR496	microRNA 496	0.235289543	0.538325548	-1.194041692
MIR497	microRNA 497	0.800896423	0.296691367	1.43265273
MIR5002	microRNA 5002	0.38832617	0.190834758	1.024944977
MIR5006	microRNA 5006	0.050431264	0.10965425	-1.120571453
MIR5007	microRNA 5007	0.022318309	0.152245526	-2.770100238
MIR500A	microRNA 500a	0.410729272	0.052997819	2.954182872
MIR500B	microRNA 500b	0.383971231	0.05154219	2.897172478
MIR501	microRNA 501	0.058971694	0.348524922	-2.563167293
MIR502	microRNA 502	0.019367489	0.104392249	-2.430305765
MIR504	microRNA 504	0.020967699	0.094598273	-2.173645324
MIR506	microRNA 506	0.034295422	0.014760482	1.216276203
MIR5090	microRNA 5090	0.188838597	0.479435819	-1.344184027
MIR5091	microRNA 5091	0.135474252	0.504046649	-1.895538577
MIR5093	microRNA 5093	0.11577701	0.367899798	-1.66796408
MIR5094	microRNA 5094	0.07233503	0.15379229	-1.088216793
MIR511-2	NULL	0.486277818	0.14332919	1.762448332
MIR512-2	microRNA 512-2	0.236100589	0.113312893	1.05908962
MIR514A1	microRNA 514a-1	0.012480781	0.067176023	-2.428238218
MIR514A2	microRNA 514a-2	0.183053038	0.065963763	1.472516106
MIR516A1	microRNA 516a-1	0.293021964	0.086186124	1.765481296
MIR517A	microRNA 517a	0.035641518	0.081202217	-1.187960324
MIR517B	microRNA 517b	0.048042078	0.172537247	-1.844537382
MIR518A1	microRNA 518a-1	0.061507938	0.345392138	-2.489390737
MIR518E	microRNA 518e	0.181840445	0.086703185	1.068516232
MIR5190	microRNA 5190	0.608447228	0.235329238	1.370451566
MIR5194	microRNA 5194	0.370193352	0.161491893	1.196817243
MIR519E	microRNA 519e	0.051033003	0.172806659	-1.759656364
MIR520B	microRNA 520b	0.046557764	0.102006792	-1.131571538
MIR521-1	microRNA 521-1	0.30917577	0.766967477	-1.310738143
MIR523	microRNA 523	0.040544696	0.082084276	-1.0175927
MIR524	microRNA 524	0.039666571	0.143146665	-1.851498484
MIR525	microRNA 525	0.043165663	0.090244485	-1.063954638
MIR526A1	microRNA 526a-1	0.05870422	0.128686044	-1.132319493
MIR526A2	microRNA 526a-2	0.078691251	0.206624959	-1.392739393
MIR526B	microRNA 526b	0.033533177	0.088771455	-1.40450667
MIR542	microRNA 542	0.031973113	0.263598814	-3.043412755
MIR545	microRNA 545	0.020762661	0.274674792	-3.725661289
MIR548AA1	microRNA 548aa-1	0.045893594	0.347872917	-2.922195669
MIR548AD	microRNA 548ad	0.136352582	0.056121978	1.280704265
MIR548AL	microRNA 548al	0.044106184	0.152406206	-1.788868795
MIR548AV	microRNA 548av	0.045363171	0.109988815	-1.277763439
MIR548D1	microRNA 548d-1	0.045893594	0.347872917	-2.922195669
MIR548F3	microRNA 548f-3	0.316673251	0.067966877	2.220091281
MIR548F4	microRNA 548f-4	0.265724895	0.050290801	2.401566956
MIR548I2	microRNA 548i-2	0.041485212	0.12026513	-1.535549358
MIR548I3	microRNA 548i-3	0.055481378	0.025957927	1.095828474
MIR548Y	microRNA 548y	0.022835367	0.055982916	-1.293716659
MIR549	NULL	0.092175025	0.332569263	-1.851207035
MIR550A2	microRNA 550a-2	0.049388216	0.116641247	-1.239839301
MIR550B2	microRNA 550b-2	0.049388216	0.116641247	-1.239839301
MIR554	microRNA 554	0.142472422	0.355320719	-1.318439131
MIR555	microRNA 555	0.036911895	0.104852859	-1.506208482
MIR5579	microRNA 5579	0.234433522	0.673243598	-1.52194973
MIR558	microRNA 558	0.028826136	0.058729058	-1.026697022
MIR5583-1	microRNA 5583-1	0.049450636	0.373044632	-2.915287267
MIR5583-2	microRNA 5583-2	0.049450634	0.375981972	-2.926602563
MIR563	microRNA 563	0.044150741	0.260065991	-2.55836819
MIR5687	microRNA 5687	0.173474034	0.051116389	1.762861914
MIR569	microRNA 569	0.076819106	0.167036292	-1.120624517
MIR5692A1	microRNA 5692a-1	0.096685919	0.348793997	-1.850997509

MIR5703	microRNA 5703	0.397834059	0.070152488	2.503600627
MIR573	microRNA 573	0.12684026	0.308944769	-1.28433621
MIR586	microRNA 586	0.007439442	0.020087639	-1.433041784
MIR589	microRNA 589	0.217218931	0.64497312	-1.570089196
MIR590	microRNA 590	0.065902963	0.15306234	-1.215704136
MIR591	microRNA 591	0.16430065	0.055696159	1.560688454
MIR595	microRNA 595	0.336308071	0.132931289	1.339102678
MIR597	microRNA 597	0.155091432	0.044794154	1.791736637
MIR599	microRNA 599	0.015734681	0.04714927	-1.583287489
MIR610	microRNA 610	0.021382251	0.054770644	-1.356989104
MIR613	microRNA 613	0.189204267	0.052393856	1.852475087
MIR618	microRNA 618	0.136709176	0.046440996	1.557639272
MIR624	microRNA 624	0.040098956	0.101281137	-1.336728925
MIR628	microRNA 628	0.019394248	0.255877391	-3.72175197
MIR632	microRNA 632	0.073569739	0.254286962	-1.789273122
MIR641	microRNA 641	0.244712421	0.654319969	-1.418908973
MIR648	microRNA 648	0.417428814	0.132257755	1.658177871
MIR651	microRNA 651	0.107994336	0.051446594	1.069808177
MIR654	microRNA 654	0.326577447	0.115485507	1.499713354
MIR655	microRNA 655	0.518358125	0.236841242	1.130028852
MIR658	microRNA 658	0.414108062	0.194688855	1.088836989
MIR7-1	microRNA 7-1	0.116748692	0.382495099	-1.71203488
MIR708	microRNA 708	0.105939295	0.3196297	-1.593163664
MIR711	microRNA 711	0.156134497	0.526715356	-1.754234192
MIR744	microRNA 744	0.042969541	0.127226061	-1.566007962
MIR758	microRNA 758	0.188691577	0.391090109	-1.051471026
MIR760	microRNA 760	0.455004734	0.225787122	1.010918353
MIR766	microRNA 766	0.123715753	0.04202199	1.557812841
MIR873	microRNA 873	0.168691038	0.080611325	1.065328894
MIR874	microRNA 874	0.211072244	0.62892706	-1.575155833
MIR875	microRNA 875	0.016661825	0.081128344	-2.283659601
MIR876	microRNA 876	0.270806343	0.127443278	1.087406251
MIR888	microRNA 888	0.018712264	0.072238167	-1.948777155
MIR889	microRNA 889	0.235645927	0.670301577	-1.508189582
MIR890	microRNA 890	0.018712262	0.046393216	-1.309929876
MIR891A	microRNA 891a	0.008317541	0.024975934	-1.586309652
MIR891B	microRNA 891b	0.017851951	0.036833873	-1.04495135
MIR892A	microRNA 892a	0.025282503	0.211000844	-3.061037565
MIR892B	microRNA 892b	0.015868417	0.300059089	-4.241016511
MIR938	microRNA 938	0.046669208	0.255156081	-2.450837136
MIR939	microRNA 939	0.254032793	0.564505111	-1.151971896
MIR942	microRNA 942	0.114123393	0.345005296	-1.596023963
MIR944	microRNA 944	0.011571458	0.0280914	-1.279557934
MIR98	microRNA 98	0.099324542	0.037472609	1.406313801
MIR99A	microRNA 99a	0.110151849	0.31506717	-1.516165725
MIRLET7C	microRNA let-7c	0.11148903	0.029746917	1.906089722
MIRLET7G	microRNA let-7g	0.128962156	0.058520501	1.139933743
MIRLET7I	microRNA let-7i	0.078071565	0.15884156	-1.024719345
MLL/AF6 fusion	NULL	0.050110333	0.102849719	-1.037357837
MLLT11	MLLT11 transcription factor 7 cofactor	0.176491526	0.384260108	-1.122484293
MPC1L	mitochondrial pyruvate carrier 1 like	0.026071447	0.089310233	-1.776354821
MT1HL1	metallothionein 1H like 1	0.198096776	0.465261885	-1.231837602
NAP1L6	NULL	0.029427885	0.134864863	-2.196258761
NDUFAF4P1	NADH:ubiquinone oxidoreductase complex assembly factor 4 pseudogene 1	0.051432631	0.108780864	-1.080668922
NGFRAP1	NULL	0.090717394	0.335497947	-1.886852836
NUDT9P1	nudix hydrolase 9 pseudogene 1	0.058704242	0.167140605	-1.509525597
OR10A6	olfactory receptor family 10 subfamily A member 6 (gene/pseudogene)	0.05472376	0.119339599	-1.124833577
OR10C1	olfactory receptor family 10 subfamily C member 1	0.318910855	0.143442265	1.15268303
OR10G3	olfactory receptor family 10 subfamily G member 3	0.610524483	1.34750624	-1.142170897
OR10H4	olfactory receptor family 10 subfamily H member 4	0.105266404	0.236237327	-1.166191864
OR10J1	olfactory receptor family 10 subfamily J member 1	0.19769108	0.399115422	-1.013558249
OR10R2	olfactory receptor family 10 subfamily R member 2	0.093672664	0.204739267	-1.128087828
OR10X1	olfactory receptor family 10 subfamily X member 1	0.067654726	0.324422489	-2.261611209
OR11H2	olfactory receptor family 11 subfamily H member 2	0.084173888	0.185585839	-1.140641969
OR14C36	olfactory receptor family 14 subfamily C member 36	0.017071912	0.100094924	-2.551672264
OR14I1	olfactory receptor family 14 subfamily I member 1	0.073979804	0.172872005	-1.224500883
OR1L3	olfactory receptor family 1 subfamily L member 3	0.29100282	0.141582469	1.0393905
OR1L4	olfactory receptor family 1 subfamily L member 4	0.20528655	0.078834082	1.380747724
OR1L6	olfactory receptor family 1 subfamily L member 6	0.132109042	0.307558851	-1.219133279
OR2AP1	olfactory receptor family 2 subfamily AP member 1	0.066669675	0.205586516	-1.624643041

OR2G2	olfactory receptor family 2 subfamily G member 2	0.107611054	0.032988379	1.705796482
OR2L2	olfactory receptor family 2 subfamily L member 2	0.030297084	0.112513378	-1.8928457
OR2L5	olfactory receptor family 2 subfamily L member 5	0.150210734	0.038945593	1.947455939
OR2M4	olfactory receptor family 2 subfamily M member 4	0.067904365	0.220455682	-1.698912451
OR2M7	olfactory receptor family 2 subfamily M member 7	0.01547167	0.120486755	-2.961173727
OR2T1	olfactory receptor family 2 subfamily T member 1	0.059604573	0.199772786	-1.744865135
OR2T10	olfactory receptor family 2 subfamily T member 10	0.14449178	0.041904631	1.785805845
OR2T12	olfactory receptor family 2 subfamily T member 12	0.037299723	0.286423863	-2.940914869
OR2T34	olfactory receptor family 2 subfamily T member 34	0.051942299	0.105061439	-1.016251482
OR2T5	olfactory receptor family 2 subfamily T member 5	0.076462542	0.157233672	-1.040085148
OR2Z1	olfactory receptor family 2 subfamily Z member 1	0.160449336	0.343936463	-1.100024257
OR3A3	olfactory receptor family 3 subfamily A member 3	0.085613637	0.199472881	-1.220280105
OR4A15	olfactory receptor family 4 subfamily A member 15	0.15734694	0.049174068	1.677979508
OR4A47	olfactory receptor family 4 subfamily A member 47	0.045470176	0.135221177	-1.572328627
OR4C13	olfactory receptor family 4 subfamily C member 13	0.042603973	0.164285847	-1.94714832
OR4C16	olfactory receptor family 4 subfamily C member 16	0.071844696	0.019896454	1.852370346
OR4C46	olfactory receptor family 4 subfamily C member 46	0.103385423	0.231779214	-1.16471841
OR4C6	olfactory receptor family 4 subfamily C member 6	0.052432583	0.018866642	1.474625992
OR4E2	olfactory receptor family 4 subfamily E member 2	0.118424826	0.248608002	-1.069901178
OR4K1	olfactory receptor family 4 subfamily K member 1	0.064619241	0.132757437	-1.038756983
OR4M2	olfactory receptor family 4 subfamily M member 2	0.103149114	0.237732071	-1.204605112
OR4N3P	olfactory receptor family 4 subfamily N member 3 pseudogene	0.220780596	0.1006641	1.133064117
OR4Q3	olfactory receptor family 4 subfamily Q member 3	0.110294445	0.260587627	-1.240408451
OR4S2	olfactory receptor family 4 subfamily S member 2	0.010729007	0.031624006	-1.559503577
OR51A4	olfactory receptor family 51 subfamily A member 4	0.48998218	0.240760565	1.025130172
OR52B6	olfactory receptor family 52 subfamily B member 6	0.092099214	0.200746078	-1.124111057
OR52W1	olfactory receptor family 52 subfamily W member 1	0.262987894	0.580504009	-1.142309642
OR5AS1	olfactory receptor family 5 subfamily AS member 1	0.043379646	0.094094242	-1.117088178
OR5B12	olfactory receptor family 5 subfamily B member 12	0.120707013	0.285932906	-1.244167164
OR5D13	olfactory receptor family 5 subfamily D member 13	0.052682292	0.142542549	-1.436002619
OR5D18	olfactory receptor family 5 subfamily D member 18	0.008910374	0.070530522	-2.984689814
OR5H1	olfactory receptor family 5 subfamily H member 1	0.345049101	0.150476928	1.197259371
OR5K1	olfactory receptor family 5 subfamily K member 1	0.557013141	0.231335919	1.267722078
OR5L2	olfactory receptor family 5 subfamily L member 2	0.022171197	0.129607214	-2.547387455
OR5M1	olfactory receptor family 5 subfamily M member 1	0.022202349	0.09701423	-2.127484088
OR5M11	olfactory receptor family 5 subfamily M member 11	0.03063587	0.190000544	-2.632709819
OR5M3	olfactory receptor family 5 subfamily M member 3	0.016363184	0.067793033	-2.050683517
OR5M9	olfactory receptor family 5 subfamily M member 9	0.082863368	0.184443097	-1.154369435
OR5P2	olfactory receptor family 5 subfamily P member 2	0.260598682	0.526185271	-1.013741076
OR5T3	olfactory receptor family 5 subfamily T member 3	0.029494686	0.08974934	-1.605446279
OR5W2	olfactory receptor family 5 subfamily W member 2	0.065577612	0.159627876	-1.283437347
OR6C1	olfactory receptor family 6 subfamily C member 1	0.104526513	0.225022496	-1.106200309
OR6C2	olfactory receptor family 6 subfamily C member 2	0.210510603	0.540068161	-1.359248598
OR6F1	olfactory receptor family 6 subfamily F member 1	0.030555553	0.068536056	-1.165428605
OR6N2	olfactory receptor family 6 subfamily N member 2	0.106536802	0.348924758	-1.711564092
OR7D4	olfactory receptor family 7 subfamily D member 4	0.122980215	0.304173816	-1.306469731
OR8G2	NULL	0.242911637	0.494395858	-1.025235051
OR8H2	olfactory receptor family 8 subfamily H member 2	0.055066978	0.116784617	-1.084590911
OR8H3	olfactory receptor family 8 subfamily H member 3	0.101972455	0.048961174	1.058469452
OR8I2	olfactory receptor family 8 subfamily I member 2	0.097728977	0.035825774	1.447788537
OR8J1	olfactory receptor family 8 subfamily J member 1	0.099191059	0.048739566	1.025116671
OR8K1	olfactory receptor family 8 subfamily K member 1	0.007158604	0.084387206	-3.559274108
OR8K3	olfactory receptor family 8 subfamily K member 3 (gene/pseudogene)	0.037562646	0.123398192	-1.715950677
OR9G4	olfactory receptor family 9 subfamily G member 4	0.094635475	0.237227972	-1.325821133
OR9G9	olfactory receptor family 9 subfamily G member 9	0.124468992	0.255920755	-1.03991078
PAR-SN	NULL	0.198676256	0.099091239	1.003590058
PAR4	NULL	0.134912654	0.389204044	-1.528501028
PCDHX	NULL	0.004345983	0.010054688	-1.210113807
PCNAP1	proliferating cell nuclear antigen pseudogene 1	0.039318904	0.099191154	-1.334988368
PGAM4	phosphoglycerate mutase family member 4	0.023236539	0.083917898	-1.852583341
PGK2	phosphoglycerate kinase 2	0.057634387	0.115750496	-1.006016632
PLN	phospholamban	0.076172305	0.198859119	-1.384408272
PMCH	pro-melanin concentrating hormone	0.011442196	0.039423554	-1.784693878
PNMA1	PNMA family member 1	0.139373121	0.296188812	-1.087564791
POM121L12	POM121 transmembrane nucleoporin like 12	0.084182792	0.194515118	-1.208285033
POU5F1P3	POU class 5 homeobox 1 pseudogene 3	0.535443382	0.256164023	1.063666163
POU5F1P4	POU class 5 homeobox 1 pseudogene 4	0.275174533	0.944493202	-1.779193455
POU5F2	POU domain class 5, transcription factor 2	0.05459451	0.183678432	-1.750354445
PPP1R2P9	NULL	0.015712403	0.058251097	-1.890381415
PRR20E	proline rich 20E	0.088274738	0.18035353	-1.03075513

PTRHD1	peptidyl-tRNA hydrolase domain containing 1	0.084011937	0.292679386	-1.800654899
PUS7L	pseudouridine synthase 7 like	0.256778404	0.554645071	-1.111040983
PYDC2	pyrin domain containing 2	0.746658338	0.366644108	1.026067878
RAB40AL	RAB40A like	0.047921721	0.154396334	-1.687886887
RAB9BP1	RAB9B, member RAS oncogene family pseudogene 1	0.277880153	0.061575139	2.17404291
RASSF9	Ras association domain family member 9	0.058268961	0.127291551	-1.127337179
RBM43	RNA binding motif protein 43	0.088237176	0.176997431	-1.004269893
RBM7	RNA binding motif protein 7	0.201083154	0.436537641	-1.118313834
REG1B	regenerating family member 1 beta	0.104103085	0.211391655	-1.021905603
REG3A	regenerating family member 3 alpha	0.120638591	0.315699861	-1.387862139
RFPL4B	ret finger protein like 4B	0.07775962	0.248970711	-1.678882969
RGS1	regulator of G protein signaling 1	0.062885279	0.211272954	-1.748313856
RNF113A	ring finger protein 113A	0.101152223	0.208445728	-1.043143779
RNF146	ring finger protein 146	0.682938351	1.40272267	-1.038402548
RNU12	RNA, U12 small nuclear	0.082190387	0.314211178	-1.934692943
RNU4ATAC	RNA, U4atac small nuclear	0.121357664	0.555645476	-2.194899459
RNU6-57P	RNA, U6 small nuclear 57, pseudogene	0.293940303	0.730941682	-1.314233123
RNU6-78P	RNA, U6 small nuclear 78, pseudogene	0.232904625	0.048035662	2.277561528
RNU6ATAC	RNA, U6atac small nuclear	0.181078335	0.466952035	-1.366660423
RNY5	RNA, Ro60-associated Y5	0.035690555	0.298916116	-3.066126461
RPA4	replication protein A4	0.031901786	0.014660538	1.121699174
RPL13P5	ribosomal protein L13 pseudogene 5	0.225193367	0.478522983	-1.087423882
RPS10P7	ribosomal protein S10 pseudogene 7	0.045117994	0.118731301	-1.395925501
RPS14P3	ribosomal protein S14 pseudogene 3	0.05470148	0.132696584	-1.278479468
RPS8	ribosomal protein S8	0.153588609	0.314046767	-1.031908198
RPSAP58	NULL	0.052929764	0.125360628	-1.24393318
RWDD2A	RWD domain containing 2A	0.236928222	0.47645768	-1.007898019
SCARNA1	small Cajal body-specific RNA 1	0.303265127	0.082253764	1.882426014
SCARNA10	small Cajal body-specific RNA 10	0.320671751	0.076839659	2.061174246
SCARNA23	small Cajal body-specific RNA 23	0.016256214	0.035356504	-1.120984331
SCARNA27	small Cajal body-specific RNA 27	0.179215136	0.08418298	1.090091999
SCARNA5	small Cajal body-specific RNA 5	0.128623288	0.052293901	1.298437288
SCARNA7	small Cajal body-specific RNA 7	0.192204128	0.064899196	1.566366818
SCARNA9	small Cajal body-specific RNA 9	0.167955551	0.429170727	-1.353472202
SCARNA9L	small Cajal body-specific RNA 9 like	0.021859177	0.089588316	-2.035071498
SMIM15	small integral membrane protein 15	0.211257217	0.506757693	-1.262295459
SNAR-E	small NF90 (ILF3) associated RNA E	0.039737901	0.090496512	-1.187346536
SNORA11B	small nucleolar RNA, H/ACA box 11B	0.093400825	0.478540788	-2.357134699
SNORA11C	small nucleolar RNA, H/ACA box 11C	0.467556936	0.228020665	1.035977481
SNORA15	small nucleolar RNA, H/ACA box 15	0.302966431	0.059267885	2.353835482
SNORA16	NULL	0.054737118	0.44351458	-3.018390162
SNORA18	small nucleolar RNA, H/ACA box 18	0.047190723	0.352109499	-2.899448968
SNORA23	small nucleolar RNA, H/ACA box 23	0.040281688	0.196917877	-2.289398059
SNORA28	small nucleolar RNA, H/ACA box 28	0.154997721	0.3891346	-1.328022265
SNORA2A	small nucleolar RNA, H/ACA box 2A	0.134315344	0.295044687	-1.135309354
SNORA2B	small nucleolar RNA, H/ACA box 2B	0.041587748	0.08594278	-1.047217898
SNORA30	small nucleolar RNA, H/ACA box 30	0.507736136	0.128412293	1.983295628
SNORA36B	small nucleolar RNA, H/ACA box 36B	0.031808141	0.084317697	-1.406439397
SNORA37	small nucleolar RNA, H/ACA box 37	0.033203325	0.180132609	-2.439659742
SNORA38	small nucleolar RNA, H/ACA box 38	0.084107077	0.304026185	-1.853896488
SNORA46	small nucleolar RNA, H/ACA box 46	0.05184873	0.238879067	-2.203899879
SNORA47	small nucleolar RNA, H/ACA box 47	0.111395273	0.383181849	-1.782341214
SNORA48	small nucleolar RNA, H/ACA box 48	0.203463384	0.416826125	-1.034676516
SNORA50	NULL	0.288372954	0.75902509	-1.396211714
SNORA53	small nucleolar RNA, H/ACA box 53	0.12655059	0.059467757	1.089534661
SNORA54	small nucleolar RNA, H/ACA box 54	0.171124925	0.645329442	-1.914985937
SNORA56	small nucleolar RNA, H/ACA box 56	0.326448227	0.133630773	1.288601934
SNORA6	small nucleolar RNA, H/ACA box 6	0.502863866	0.216114867	1.218369567
SNORA61	small nucleolar RNA, H/ACA box 61	0.171699947	0.403708338	-1.233423788
SNORA63	small nucleolar RNA, H/ACA box 63	1.043264023	2.088577729	-1.001416524
SNORA66	small nucleolar RNA, H/ACA box 66	0.308956842	0.120843045	1.354270884
SNORA67	small nucleolar RNA, H/ACA box 67	0.811313584	0.332139144	1.288471886
SNORA69	small nucleolar RNA, H/ACA box 69	0.063192852	0.271993703	-2.105739965
SNORA7	NULL	0.24561691	0.654384079	-1.413727758
SNORA70G	small nucleolar RNA, H/ACA box 70G	0.085885434	0.037694189	1.188071352
SNORA71B	small nucleolar RNA, H/ACA box 71B	0.442345639	0.214007591	1.047512131
SNORA71C	small nucleolar RNA, H/ACA box 71C	0.220094181	0.526732799	-1.258950296
SNORA74A	small nucleolar RNA, H/ACA box 74A	0.222202324	0.068536093	1.69693806
SNORA76	NULL	0.163685454	0.34550492	-1.077780131
SNORA78	small nucleolar RNA, H/ACA box 78	0.199005906	0.429409888	-1.109544164

SNORA8	small nucleolar RNA, H/ACA box 8	0.084967398	0.192716247	-1.18149691
SNORD103A	small nucleolar RNA, C/D box 103A	0.346439594	0.918839321	-1.407208776
SNORD104	small nucleolar RNA, C/D box 104	0.276493893	0.574764786	-1.055724062
SNORD105B	small nucleolar RNA, C/D box 105B	0.518117319	0.24519268	1.079362898
SNORD109B	small nucleolar RNA, C/D box 109B	0.142731013	0.06871859	1.054526502
SNORD11	small nucleolar RNA, C/D box 11	0.026445887	0.06827106	-1.368230787
SNORD110	small nucleolar RNA, C/D box 110	0.249241038	0.583550014	-1.227314681
SNORD111	small nucleolar RNA, C/D box 111	0.035677215	0.175487621	-2.298294355
SNORD113-4	small nucleolar RNA, C/D box 113-4	0.027778644	0.085699484	-1.625310333
SNORD113-5	small nucleolar RNA, C/D box 113-5	0.044703461	0.200745944	-2.166912403
SNORD113-6	small nucleolar RNA, C/D box 113-6	0.050025622	0.13356559	-1.416809274
SNORD113-9	small nucleolar RNA, C/D box 113-9	0.175894364	0.074801786	1.233564644
SNORD114-1	small nucleolar RNA, C/D box 114-1	0.286371405	0.051842002	2.465694105
SNORD114-10	small nucleolar RNA, C/D box 114-10	0.145815676	0.061184102	1.252917087
SNORD114-11	small nucleolar RNA, C/D box 114-11	0.201292768	0.086477229	1.21890314
SNORD114-14	small nucleolar RNA, C/D box 114-14	0.137507023	0.040014508	1.780910223
SNORD114-16	small nucleolar RNA, C/D box 114-16	0.053689629	0.254291236	-2.243766411
SNORD114-2	small nucleolar RNA, C/D box 114-2	0.076132664	0.336684326	-2.144809101
SNORD114-23	small nucleolar RNA, C/D box 114-23	0.034491504	0.086768317	-1.330927307
SNORD114-29	small nucleolar RNA, C/D box 114-29	0.181163087	0.058055592	1.641782083
SNORD114-30	small nucleolar RNA, C/D box 114-30	0.109625752	0.252557642	-1.204025957
SNORD114-31	small nucleolar RNA, C/D box 114-31	0.045706387	0.143572519	-1.651311945
SNORD114-8	small nucleolar RNA, C/D box 114-8	0.103817596	0.211982819	-1.029896358
SNORD114-9	small nucleolar RNA, C/D box 114-9	0.055098166	0.32481354	-2.559535583
SNORD115-1	small nucleolar RNA, C/D box 115-1	0.740998248	0.142290685	2.380628912
SNORD115-14	small nucleolar RNA, C/D box 115-14	0.086193136	0.345309208	-2.002243916
SNORD115-27	small nucleolar RNA, C/D box 115-27	0.454964628	0.213985729	1.088239804
SNORD115-28	small nucleolar RNA, C/D box 115-28	0.527629523	0.198508285	1.410326074
SNORD115-32	small nucleolar RNA, C/D box 115-32	0.151904293	0.432959821	-1.511070504
SNORD115-35	small nucleolar RNA, C/D box 115-35	0.11328984	0.404655507	-1.836675745
SNORD115-37	small nucleolar RNA, C/D box 115-37	0.572172394	0.246535262	1.214655884
SNORD115-38	small nucleolar RNA, C/D box 115-38	0.173005708	0.382599642	-1.145015888
SNORD115-40	small nucleolar RNA, C/D box 115-40	0.135612563	0.581038435	-2.099142769
SNORD115-41	small nucleolar RNA, C/D box 115-41	0.107027119	0.388730599	-1.860794275
SNORD115-44	small nucleolar RNA, C/D box 115-44	0.178506404	0.443071144	-1.311562537
SNORD115-48	small nucleolar RNA, C/D box 115-48	0.036390396	0.082279765	-1.176979934
SNORD115-5	small nucleolar RNA, C/D box 115-5	0.129710897	0.362611826	-1.483126295
SNORD116-11	small nucleolar RNA, C/D box 116-11	0.060678844	0.502730109	-3.050518589
SNORD116-16	small nucleolar RNA, C/D box 116-16	0.19077747	0.072033939	1.405142104
SNORD116-18	small nucleolar RNA, C/D box 116-18	0.05977845	0.402491627	-2.751261375
SNORD116-24	small nucleolar RNA, C/D box 116-24	0.04304086	0.108485419	-1.33372234
SNORD116-28	small nucleolar RNA, C/D box 116-28	0.08031362	0.03340987	1.265370299
SNORD116-29	small nucleolar RNA, C/D box 116-29	0.119802102	0.047792325	1.325802378
SNORD116-4	small nucleolar RNA, C/D box 116-4	0.107499444	0.230788364	-1.102241293
SNORD117	small nucleolar RNA, C/D box 117	0.346257034	0.136629063	1.341578978
SNORD11B	small nucleolar RNA, C/D box 11B	0.170857543	0.054175373	1.65708485
SNORD121B	small nucleolar RNA, C/D box 121B	0.1166864	0.35559432	-1.607595862
SNORD123	small nucleolar RNA, C/D box 123	0.09756407	0.246665654	-1.338135003
SNORD127	small nucleolar RNA, C/D box 127	0.024627246	0.05347577	-1.118630033
SNORD12C	small nucleolar RNA, C/D box 12C	0.144910567	0.304638591	-1.071935912
SNORD14	NULL	0.110232065	0.499936251	-2.181200204
SNORD17	small nucleolar RNA, C/D box 17	0.356999345	0.176413257	1.01696245
SNORD18C	small nucleolar RNA, C/D box 18C	0.266290767	0.115298647	1.207626828
SNORD19	small nucleolar RNA, C/D box 19	0.093414208	0.227977057	-1.287174742
SNORD19B	small nucleolar RNA, C/D box 19B	0.054848558	0.202948995	-1.887591595
SNORD1A	small nucleolar RNA, C/D box 1A	0.352132005	0.152945024	1.203103193
SNORD1C	small nucleolar RNA, C/D box 1C	0.117675938	0.410043642	-1.800958115
SNORD21	small nucleolar RNA, C/D box 21	0.236782561	0.088241376	1.424035635
SNORD27	small nucleolar RNA, C/D box 27	0.287846821	0.961247722	-1.739606998
SNORD28	small nucleolar RNA, C/D box 28	0.119236031	0.40008016	-1.746468829
SNORD33	small nucleolar RNA, C/D box 33	0.088399601	0.292302667	-1.725351227
SNORD34	small nucleolar RNA, C/D box 34	0.130976677	0.333846691	-1.349875807
SNORD35A	small nucleolar RNA, C/D box 35A	0.518238247	0.188970669	1.455453172
SNORD38A	small nucleolar RNA, C/D box 38A	0.076319966	0.199533803	-1.386500746
SNORD41	small nucleolar RNA, C/D box 41	0.099872982	0.520206314	-2.380917564
SNORD44	small nucleolar RNA, C/D box 44	0.281454968	0.103349471	1.445373107
SNORD45B	small nucleolar RNA, C/D box 45B	0.09509466	0.193563517	-1.025370823
SNORD45C	small nucleolar RNA, C/D box 45C	0.211473566	0.103240863	1.034463229
SNORD4B	small nucleolar RNA, C/D box 4B	0.13224279	0.277168548	-1.067574488
SNORD52	small nucleolar RNA, C/D box 52	0.240041174	0.09568029	1.326988228

SNORD55	small nucleolar RNA, C/D box 55	0.109358397	0.371467493	-1.76417197
SNORD56	small nucleolar RNA, C/D box 56	0.078160843	0.190056905	-1.281913518
SNORD59A	small nucleolar RNA, C/D box 59A	0.627226421	0.248929365	1.333249904
SNORD61	small nucleolar RNA, C/D box 61	0.034001206	0.13160159	-1.952519112
SNORD62A	small nucleolar RNA, C/D box 62A	0.731801537	0.332260773	1.139136468
SNORD7	small nucleolar RNA, C/D box 7	0.302338163	0.635918824	-1.072679518
SNORD71	small nucleolar RNA, C/D box 71	0.058307542	0.122368121	-1.069473361
SNORD73A	small nucleolar RNA, C/D box 73A	0.110856091	0.417474085	-1.912998607
SNORD80	small nucleolar RNA, C/D box 80	0.822609568	0.147691725	2.477618861
SNORD84	small nucleolar RNA, C/D box 84	0.106434303	0.213360166	-1.003327657
SNORD87	small nucleolar RNA, C/D box 87	0.300434686	0.097613849	1.621893633
SNORD89	small nucleolar RNA, C/D box 89	0.095393143	0.220316516	-1.207620183
SNORD91A	small nucleolar RNA, C/D box 91A	0.070864091	0.153023205	-1.110623795
SNORD92	small nucleolar RNA, C/D box 92	0.049053902	0.145432519	-1.567910104
SNORD94	small nucleolar RNA, C/D box 94	0.093249286	0.264024475	-1.501507087
SNORD97	small nucleolar RNA, C/D box 97	0.096578813	0.252205622	-1.384821806
SnoZ5	NULL	0.03229847	0.235602765	-2.86681876
SnR39B	NULL	0.031340124	0.090904904	-1.536347252
SPIN4	spindlin family member 4	0.078241142	0.024206833	1.692513106
SPRR2D	small proline rich protein 2D	1.116455431	2.235060353	-1.00138813
SUMO4	small ubiquitin like modifier 4	0.040348573	0.150668142	-1.900784861
SYT14L	NULL	0.608828893	0.254830123	1.256501003
TAS2R13	taste 2 receptor member 13	0.027952447	0.103527608	-1.88896905
TAS2R14	taste 2 receptor member 14	0.114787436	0.038445911	1.578062683
TAS2R3	taste 2 receptor member 3	0.046504236	0.128733786	-1.468956696
TAS2R43	taste 2 receptor member 43	0.129505984	0.047466426	1.44803943
TCEAL3	transcription elongation factor A like 3	0.174129171	0.424505529	-1.285625432
TCRAV5.1a	NULL	0.052133873	0.11295218	-1.115419169
TCRBV	NULL	1.312420907	0.539459567	1.282643745
TMEM203	transmembrane protein 203	0.156031983	0.337084027	-1.111266491
TPI1P3	triosephosphate isomerase 1 pseudogene 3	0.137582798	0.452938953	-1.719016515
TRAV3J15	NULL	0.70967019	0.082006083	3.11334588
TRIM51HP	tripartite motif-containing 51H, pseudogene	0.041890798	0.191851458	-2.195284457
TRIpartite motif protein	NULL	0.051197941	0.118726933	-1.213489549
TRNA_Sup	NULL	0.368303168	0.764477806	-1.053580814
TTC3P1	tetratricopeptide repeat domain 3 pseudogene 1	0.024688173	0.085140409	-1.786023944
TTTY10	testis expressed transcript, Y-linked 10	0.022941431	0.055404804	-1.272055708
TTTY12	testis expressed transcript, Y-linked 12	0.024462317	0.096752059	-1.983731335
TWIST1	twist family bHLH transcription factor 1	0.140631479	0.297771888	-1.082287995
TXNDC16	thioredoxin domain containing 16	0.203871726	0.485153886	-1.25078072
UBE2MP1	ubiquitin conjugating enzyme E2 M pseudogene 1	0.138073059	0.06417356	1.105380923
UBE2NL	ubiquitin conjugating enzyme E2 N like (gene/pseudogene)	0.048082203	0.131015004	-1.446157137
UBTFL1	upstream binding transcription factor like 1	0.135117713	0.327377057	-1.276736408
UGT2B17	UDP glucuronosyltransferase family 2 member B17	0.006412658	0.002400206	1.417764424
USP17L1P	NULL	0.2172903	0.105478547	1.042674174
USP17L3	ubiquitin specific peptidase 17 like family member 3	0.07599456	0.035061023	1.116028048
V alpha 18/J alpha F/C alpha	NULL	0.069250326	0.026674899	1.376338081
VCY	variable charge Y-linked	0.099734743	0.256346587	-1.361927628
VIS1	viral integration site 1	0.007635557	0.03613862	-2.242736108
VTRNA1-1	vault RNA 1-1	0.100470312	0.383724917	-1.933303194
VTRNA1-2	vault RNA 1-2	0.087401112	0.246413596	-1.495358325
WAPL	WAPL cohesin release factor	0.170113065	0.368112915	-1.11365442
X74394	NULL	0.163734517	0.486709589	-1.571702713
Y16709	NULL	0.195560455	0.071577701	1.450032562
YY2	YY2 transcription factor	0.080615504	0.01851903	2.122048821
Z49981	NULL	0.016002136	0.057864376	-1.854410984
ZBED2	zinc finger BED-type containing 2	0.124082562	0.258708962	-1.060029649
ZCCHC5	NULL	0.147483443	0.31380268	-1.089304677
ZFAT-AS1	ZFAT antisense RNA 1	0.060763559	0.155927259	-1.359594894
ZNF561	zinc finger protein 561	0.145253994	0.298233527	-1.037864617
ZNF606	zinc finger protein 606	0.160845442	0.326253222	-1.020317096
ZNF674-AS1	ZNF674 antisense RNA 1 (head to head)	0.052888792	0.112627794	-1.090528974
ZNF727	zinc finger protein 727	0.041611976	0.099468945	-1.257247394
ZNF749	zinc finger protein 749	0.082764568	0.171155567	-1.048223048
ZNF830	zinc finger protein 830	0.284793655	0.138180154	1.043366585

Supplementary Table 7: Genes Exhibiting Significant Changes in H3K14ac Deposition Identified by CUT&Tag in hSSCs Cultured with Wnt3a

Gene	Description	Active Wnt3a	Inactive Wnt3a	Fold change
AAAS	aladin WD repeat nucleoporin	0.765735792	0.372517339	1.039539115
AB062081	NULL	0.598315927	0.234701275	1.350081787
AB586698	NULL	0.205644454	0.085544977	1.26539711
ABHD11	abhydrolase domain containing 11	2.384586146	1.097520815	1.119490601
AJ297365	NULL	0.719855811	0.259799317	1.470310325
AK024535	NULL	0.410888936	0.158079204	1.378100896
AK057604	NULL	0.203735636	0.076020357	1.422240643
AK057689	NULL	0.355847354	0.139107838	1.355054803
AK057978	NULL	0.236006222	0.494580248	-1.067379728
AK097453	NULL	0.300572906	0.148020217	1.02192073
AK125701	NULL	0.392300449	0.147107223	1.4150909
AK299432	NULL	0.212780809	0.090260581	1.237200064
AK310794	NULL	0.456278956	0.217955458	1.065882787
AKR7A2P1	AKR7A2 pseudogene 1	0.564617979	0.272135546	1.052949656
AMELY	amelogenin Y-linked	0.060156236	0.154457481	-1.360423539
AMH	anti-Mullerian hormone	0.569699977	0.271445663	1.069538913
APOC1	apolipoprotein C1	1.372907755	0.634033932	1.114602738
APOC2	apolipoprotein C2	0.429115646	0.203123876	1.079006673
ATP6AP1	ATPase H+ transporting accessory protein 1	0.813870404	0.401636815	1.018907571
AV30S1	NULL	0.168330999	0.414231521	-1.299136462
AX746640	NULL	0.30074155	0.148217392	1.02080946
AX746968	NULL	0.603379375	0.297700504	1.019203718
AX747161	NULL	0.550786977	0.205645008	1.421338401
AX747402	NULL	0.194411716	0.071700684	1.439056387
AX748200	NULL	0.653868513	0.318178067	1.039166162
B3GNT1	NULL	0.378406888	0.157667658	1.263051594
BANF1	BAF nuclear assembly factor 1	0.76568333	0.379237137	1.0136476
BC034630	NULL	0.203787418	0.087671862	1.216879192
BC042029	NULL	0.222526366	0.702636285	-1.6588018
BC042033	NULL	0.451260422	0.22342324	1.014180994
BC062469	NULL	0.666854877	0.283911621	1.231930931
BC062753	NULL	0.36780724	0.169720284	1.115790885
BC070370	NULL	0.15113871	0.332022345	-1.135407121
BC122864	NULL	0.829882788	0.393044977	1.078213173
BC127854	NULL	0.877092032	0.365539973	1.262699052
BC128459	NULL	0.42098255	0.206526901	1.027430723
BC150535	NULL	0.453165251	0.21900248	1.049090032
BEX2	brain expressed X-linked 2	1.566541081	0.662617798	1.241333743
BST2	bone marrow stromal cell antigen 2	0.458726718	0.177320267	1.371277498
C12orf10	NULL	2.122418897	1.000830042	1.084512425
C16orf3	NULL	0.479361829	0.208913296	1.198210722
C19orf52	NULL	0.511715504	0.177112798	1.530673485
C1orf56	chromosome 1 open reading frame 56	1.154777375	0.572350585	1.012643722
CDIPT	CDP-diacylglycerol--inositol 3-phosphatidyltransferase	1.952778627	0.867682744	1.170288868
CDR1	cerebellar degeneration related 1	0.085749818	0.179338229	-1.064477542
COG8	component of oligomeric golgi complex 8	0.39979421	0.194270828	1.041188297
COX6A1	cytochrome c oxidase subunit 6A1	0.466279941	0.19515558	1.256571657
CPXCR1	CPX chromosome region candidate 1	0.088349225	0.197426299	-1.160024806
DD413549	NULL	0.137947176	0.357462752	-1.373676995
DD413615	NULL	0.180112142	0.551181406	-1.613631782
DD413674	NULL	0.26654371	0.74902489	-1.490641522
DJ439583	NULL	0.463603327	0.990048688	-1.094608554
DL489931	NULL	1.293167238	0.220507714	2.552007831
DL489965	NULL	0.521319934	0.202553079	1.363869013
DL489966	NULL	1.250362969	0.380309373	1.717101558
DL490450	NULL	1.222946019	0.539560838	1.180503181
DL491209	NULL	1.043281526	0.28193013	1.887718943
DL491467	NULL	0.324725576	0.684583814	-1.076006165
DM075093	NULL	0.752571765	0.24172024	1.638490884
DM110804	NULL	1.090337754	0.311914726	1.805551536
DM119504	NULL	0.929458781	0.411295235	1.176216531
DM119532	NULL	0.612635803	0.305456198	1.004064169
DPB	NULL	0.711046141	0.151978444	2.22607647
DQ569978	NULL	0.224190648	0.455603408	-1.023052441
DQ570403	NULL	0.737396465	1.979180173	-1.424390548
DQ570455	NULL	2.292241402	0.977520661	1.229559885
DQ570460	NULL	0.464393437	1.111465935	-1.259044243
DQ570533	NULL	0.60435725	0.187659303	1.687285801
DQ570973	NULL	0.806442291	0.394404042	1.03189696
DQ571071	NULL	0.213232183	0.432533838	-1.020387804

DQ571361	NULL	0.508170701	0.181802445	1.482941595
DQ571491	NULL	0.29225449	0.642490032	-1.136448883
DQ572047	NULL	2.588684126	1.142095094	1.180536158
DQ572309	NULL	1.300411131	0.586929692	1.147708212
DQ572638	NULL	1.373925028	0.657051331	1.064225294
DQ573409	NULL	0.22937597	0.744822384	-1.699182172
DQ573543	NULL	1.935594548	0.806654068	1.262754767
DQ573683	NULL	0.396912554	1.020098005	-1.361814667
DQ573937	NULL	0.234034818	0.729030889	-1.639256766
DQ574115	NULL	0.899275109	0.263726237	1.76972143
DQ574263	NULL	0.719357472	0.285836292	1.331519766
DQ574353	NULL	1.502779281	0.655692418	1.196542013
DQ574393	NULL	2.280093145	1.114475748	1.032727539
DQ574554	NULL	0.120806584	0.274578901	-1.18452169
DQ574855	NULL	1.025693362	0.417743443	1.295910403
DQ574970	NULL	0.118578225	0.242508636	-1.032197018
DQ575983	NULL	0.944687531	0.466305561	1.018561582
DQ576238	NULL	0.215521569	0.43390325	-1.009541132
DQ576448	NULL	0.534525766	0.150074545	1.832580202
DQ576557	NULL	0.56259195	0.267051544	1.074970686
DQ576585	NULL	0.863338221	0.285696209	1.595443969
DQ576722	NULL	0.728271164	1.668360584	-1.195883505
DQ577036	NULL	0.281375956	0.619503359	-1.138613054
DQ577180	NULL	0.777741805	2.211880345	-1.50791015
DQ577458	NULL	0.339444981	0.797286531	-1.231920544
DQ577480	NULL	0.159897052	0.504144598	-1.656694248
DQ577534	NULL	0.322266564	0.905270925	-1.490095107
DQ577646	NULL	0.537393606	1.17487699	-1.128458654
DQ578285	NULL	0.836172076	0.38844862	1.106076078
DQ578665	NULL	1.684010657	0.642915032	1.38920128
DQ578778	NULL	0.517309898	0.195373439	1.404794448
DQ578892	NULL	0.241199585	0.925707766	-1.940329403
DQ579128	NULL	0.519303102	0.183929428	1.497424522
DQ579218	NULL	2.023820106	0.984767611	1.039225839
DQ579267	NULL	0.844071086	0.356155299	1.24485805
DQ579268	NULL	0.272293262	0.12846269	1.083811877
DQ579335	NULL	0.378375658	0.15970292	1.244428588
DQ579404	NULL	1.479949773	0.513679439	1.526607981
DQ579470	NULL	0.99078195	0.378063018	1.389940854
DQ579700	NULL	0.876428065	0.285270918	1.619303004
DQ579782	NULL	0.429007533	1.28872501	-1.586869566
DQ580039	NULL	1.739065468	0.829842713	1.067402423
DQ580631	NULL	0.167926662	0.462902347	-1.462876574
DQ580826	NULL	0.556997429	0.242736801	1.198277821
DQ580910	NULL	1.293383229	0.514602999	1.329618039
DQ581770	NULL	0.4158628	0.074676736	2.477376862
DQ581862	NULL	0.566286597	0.146261774	1.952979622
DQ582013	NULL	0.169346393	0.373378445	-1.140661388
DQ582057	NULL	0.424136901	0.203134327	1.062095954
DQ582610	NULL	2.445930375	1.020194556	1.261539031
DQ582763	NULL	1.498902092	0.512143943	1.549284896
DQ583130	NULL	0.597869532	0.234213604	1.352005819
DQ583138	NULL	1.110943598	0.474875373	1.226164729
DQ583414	NULL	0.233169798	0.598788609	-1.360665848
DQ583633	NULL	0.543330921	0.188821414	1.524808767
DQ583646	NULL	1.422657937	0.64722103	1.136258432
DQ583809	NULL	0.627666288	0.275362291	1.18866672
DQ583891	NULL	0.274667384	0.961544891	-1.807668608
DQ584035	NULL	0.898345767	0.338122985	1.409722743
DQ584116	NULL	0.546852125	0.200877695	1.444833388
DQ584254	NULL	0.552089311	0.18099835	1.608925122
DQ584372	NULL	1.073955216	0.522109246	1.040510223
DQ584676	NULL	0.678777248	0.284471663	1.25465326
DQ584694	NULL	0.967453788	2.553467234	-1.400192891
DQ584913	NULL	0.526068065	0.233331767	1.172866737
DQ584993	NULL	1.171109408	0.574059015	1.028604901
DQ585215	NULL	0.801384135	1.675239165	-1.063801223
DQ585295	NULL	1.936107487	0.967881859	1.000256184
DQ585389	NULL	1.158186338	0.569665238	1.023681106
DQ585433	NULL	0.173455289	0.401262284	-1.209981731
DQ585554	NULL	0.355748549	0.850858811	-1.258061885
DQ585588	NULL	0.570710903	1.435806442	-1.331029246
DQ585724	NULL	2.965978182	1.263782778	1.230759474

DQ585728	NULL	1.465846072	0.578204027	1.342083053
DQ586209	NULL	0.978703737	0.400536121	1.288939857
DQ586243	NULL	0.439970352	1.362824915	-1.631122013
DQ586485	NULL	0.765372961	1.896887456	-1.309399245
DQ586506	NULL	0.265913668	0.64157666	-1.270663727
DQ586641	NULL	0.380209027	0.765609207	-1.009815395
DQ587033	NULL	0.540406857	0.228813315	1.239874972
DQ587117	NULL	0.740837184	0.297373519	1.316880332
DQ587139	NULL	0.611093242	0.23663634	1.368720886
DQ587882	NULL	0.600647796	1.296957066	-1.110539537
DQ587889	NULL	0.912329083	0.344031812	1.407012332
DQ588228	NULL	0.353322974	0.723194792	-1.033396727
DQ588689	NULL	0.428692413	0.911564826	-1.088402374
DQ588796	NULL	1.315205273	0.498863492	1.398570991
DQ588877	NULL	0.719950412	0.168610316	2.094204738
DQ588883	NULL	1.231402646	2.65027479	-1.105839378
DQ589456	NULL	0.180934911	0.476141413	-1.395919313
DQ589665	NULL	0.594931249	0.202226422	1.556751452
DQ589679	NULL	0.446749656	1.204642466	-1.431066499
DQ590295	NULL	0.175100822	0.080813765	1.115512912
DQ590472	NULL	0.699199047	1.500112801	-1.101295865
DQ590984	NULL	0.588368259	1.248889305	-1.085854286
DQ591120	NULL	0.538747351	1.091950569	-1.019226773
DQ591184	NULL	0.814848444	1.794954202	-1.139343377
DQ592298	NULL	0.636334739	0.297990859	1.09451781
DQ592427	NULL	1.374601691	0.517590918	1.409129431
DQ592502	NULL	0.394561952	0.178155527	1.147114599
DQ592588	NULL	0.401119759	0.133567323	1.586465936
DQ592678	NULL	0.413516888	1.361585497	-1.719269421
DQ592688	NULL	1.218079538	0.5480373	1.152262349
DQ592861	NULL	1.616462522	0.70363173	1.199947611
DQ593224	NULL	0.602415652	0.264784531	1.185940414
DQ593386	NULL	1.885385078	0.711942524	1.405026535
DQ593444	NULL	0.650194666	0.322616994	1.011049286
DQ593488	NULL	0.228026721	0.536193902	-1.233551916
DQ594400	NULL	0.341630836	0.170073112	1.006283126
DQ594582	NULL	0.596069075	0.160325444	1.894476128
DQ594669	NULL	1.115344145	0.514763559	1.115507098
DQ594674	NULL	0.148980848	0.388645952	-1.383329615
DQ594798	NULL	0.598823943	0.178980386	1.742330415
DQ595000	NULL	0.517262713	0.257179548	1.00812128
DQ595071	NULL	0.727815146	1.61211463	-1.147310352
DQ595383	NULL	0.189759052	0.496757356	-1.388372624
DQ595590	NULL	0.363369161	0.178259274	1.027458846
DQ595602	NULL	0.97269561	0.452480628	1.104132381
DQ595661	NULL	1.74569722	0.604168504	1.530780472
DQ595836	NULL	0.267916329	0.619804081	-1.210029743
DQ596025	NULL	0.187352012	0.400494746	-1.096031847
DQ596112	NULL	0.121460047	0.306768485	-1.336668447
DQ596613	NULL	0.388492542	0.805403501	-1.05182484
DQ596703	NULL	2.020732248	0.686958513	1.556583295
DQ597117	NULL	2.627714199	1.30069903	1.014521197
DQ597410	NULL	1.21350473	0.577093858	1.07230185
DQ597473	NULL	0.43067617	0.179073602	1.266050824
DQ597986	NULL	0.167766856	0.49024157	-1.547035097
DQ597999	NULL	0.591193685	0.191928859	1.623059206
DQ598099	NULL	0.374478171	0.153436161	1.287243099
DQ598314	NULL	1.712236311	0.850951847	1.008732425
DQ598830	NULL	0.236714496	0.607135502	-1.358870483
DQ599224	NULL	0.456269569	0.152372726	1.582281744
DQ599327	NULL	0.68417359	0.275056101	1.314636513
DQ599532	NULL	0.295018777	0.975455117	-1.725268711
DQ599954	NULL	0.148590656	0.33159158	-1.158063982
DQ600036	NULL	1.696755021	0.70021831	1.27690159
DQ600787	NULL	0.216377205	0.46650286	-1.108337403
DQ600844	NULL	0.715272421	1.494063583	-1.062676825
DQ600899	NULL	0.295262917	0.14101182	1.066184074
DQ600902	NULL	1.801747035	0.657897345	1.453462075
DQ601004	NULL	0.58625772	0.249714359	1.231256234
DQ601165	NULL	1.504546437	0.638630106	1.236276166
DQ601360	NULL	1.484152877	0.741117938	1.001864656
DQ601842	NULL	0.715544288	0.268976149	1.411562814
DQ786325	NULL	0.562234471	0.279372325	1.008982799

DRD4	dopamine receptor D4	0.458841272	0.228439713	1.006181686
E02193	NULL	0.159361085	0.356534047	-1.161740477
FAM89B	family with sequence similarity 89 member B	1.595918826	0.725331848	1.137674172
FKSG46	NULL	0.190656946	0.396199043	-1.055246303
FLJ45513	uncharacterized LOC729220	0.609089663	0.257233134	1.243578132
FTH1P3	ferritin heavy chain 1 pseudogene 3	0.29712483	0.100345416	1.566094465
FTHL17	ferritin heavy chain like 17	0.239239149	0.479637859	-1.003492049
FUT7	fucosyltransferase 7	0.676592697	0.322179437	1.070423187
H2AFX	NULL	0.600393898	0.293825349	1.030950451
Hel-N1	NULL	0.498425245	0.204301469	1.286677563
HI650153	NULL	0.445987943	0.211626526	1.075484241
HIST1H1D	NULL	0.302328936	0.12796472	1.240372959
HIST1H1T	NULL	0.27982911	0.114419565	1.290212283
HIST1H2AE	NULL	0.372997387	0.16410729	1.1845262
HIST1H2AG	NULL	0.576874023	0.11713787	2.300048732
HIST1H2AJ	NULL	0.477974914	0.23048885	1.052237941
HIST1H2BF	NULL	0.467726624	0.068736838	2.76651016
HIST1H2BG	NULL	0.418504941	0.199093069	1.071801656
HIST1H2BJ	NULL	0.264075428	0.105813144	1.319431217
HIST1H2BM	NULL	0.707966716	0.350926155	1.012514058
HIST1H4C	NULL	0.381713373	0.139242548	1.454889615
HIST1H4D	NULL	0.401984965	0.200742676	1.001794192
HIST1H4K	NULL	0.597677044	0.2302813	1.375968872
HIST2H3D	NULL	0.380420637	0.177200923	1.102209391
HM587425	NULL	0.531089261	0.257407753	1.044898851
HMBS	hydroxymethylbilane synthase	2.476664021	1.23230851	1.007034687
HMGB3P1	high mobility group box 3 pseudogene 1	0.270939203	0.108718292	1.317374458
HOTAIR_4	NULL	1.193591078	0.410745418	1.538992272
HOXD12	homeobox D12	1.094729099	0.387561678	1.498076076
HSD17B10	hydroxysteroid 17-beta dehydrogenase 10	0.472131476	0.207837675	1.18373147
HV303525	NULL	0.297059168	0.143055635	1.054173988
HV303528	NULL	0.389202191	0.142785878	1.446666531
HV592909	NULL	0.415355026	0.100303898	2.049967339
HV592934	NULL	0.126969822	0.253952699	-1.00007417
HV592982	NULL	0.767835823	0.306161686	1.326504119
HV745899	NULL	0.104060958	0.930693728	-3.160877597
HV745905	NULL	0.250084849	1.060603713	-2.084396145
HV781757	NULL	0.931269293	0.41764492	1.156921521
HV941430	NULL	0.305629113	0.096231569	1.667199826
HV941434	NULL	0.120773662	0.307764721	-1.349521995
HV941486	NULL	0.560114408	0.254808682	1.136307104
HV983065	NULL	0.801544138	0.3123767	1.359495121
IGFLR1	IGF like family receptor 1	1.962862858	0.902469105	1.121009929
IGHV3-07	NULL	0.620440286	0.264374983	1.230706699
ILF3-AS1	NULL	0.326916248	0.157067548	1.057535951
JA202335	NULL	0.262312269	0.067180506	1.965170721
JA374918	NULL	0.247409847	1.008056721	-2.026601991
JA429818	NULL	0.037849331	0.107530506	-1.5064063
JA429845	NULL	0.280562652	0.129266746	1.117971782
JA429848	NULL	0.224312892	0.101974352	1.1373062
JA611269	NULL	1.050274971	0.361389787	1.539139448
JA700170	NULL	1.234941435	0.582312533	1.084577052
JA700174	NULL	1.21149168	0.332240291	1.866485551
JA715134	NULL	0.894274401	0.360378242	1.311205668
JA783409	NULL	0.130989474	0.050040404	1.388285543
JA783435	NULL	0.155256993	0.347715087	-1.163247419
JA783436	NULL	0.476447078	0.1872495	1.347354107
JA783516	NULL	0.058243102	0.02735466	1.090300571
JA783560	NULL	0.206057918	0.050595493	2.025969134
JA783587	NULL	0.104399478	0.030088573	1.794826931
JA783589	NULL	0.104263141	0.030088571	1.792941733
JA783602	NULL	0.01645419	0.034020838	-1.047963693
JA783603	NULL	0.016454188	0.035079124	-1.092157868
JA783612	NULL	0.049245022	0.266185411	-2.4343817
JA783645	NULL	0.04241417	0.215143634	-2.342681914
JA783654	NULL	0.042865488	0.215128074	-2.327307348
JA783672	NULL	0.201610661	0.079449411	1.343463509
JA783673	NULL	0.159572754	0.079195214	1.010729186
JA783679	NULL	0.07497463	0.163137108	-1.121610575
JA783690	NULL	0.108616394	0.358313548	-1.721980726
JA783704	NULL	0.270882623	0.090021898	1.589319966
JA783760	NULL	0.572234064	0.278111678	1.040941055
JA783782	NULL	0.097954089	0.348259888	-1.82998669

JA783787	NULL	0.505636303	0.173128548	1.546256409
JA783844	NULL	0.270436337	0.57379969	-1.085258179
JA783867	NULL	0.067372859	0.14468975	-1.102723291
JB022994	NULL	1.131552156	0.497281309	1.186168973
JB050151	NULL	0.609998463	0.237372986	1.361649847
JB153432	NULL	0.616585104	0.285146379	1.112597324
JB155089	NULL	0.478261723	0.124224349	1.944852355
JB175196	NULL	0.550956325	0.236330224	1.221133812
JB175310	NULL	0.192622114	0.597289677	-1.632657445
KCNJ14	potassium inwardly rectifying channel subfamily J member 14	0.728421017	0.340449673	1.097331
KIAA0855	NULL	0.923282116	0.433078507	1.092142966
KIAA0913	NULL	0.545456112	0.260639671	1.065406432
KRTAP19-1	keratin associated protein 19-1	0.270708915	0.702947278	-1.376674089
KRTAP20-3	keratin associated protein 20-3	0.649902431	0.31247518	1.056481544
KRTAP25-1	keratin associated protein 25-1	0.3604264	0.781955501	-1.117381822
KRTAP6-1	keratin associated protein 6-1	0.676991057	0.177683485	1.929827184
LENEP	lens epithelial protein	1.27587447	0.329360856	1.953745385
LINC00244	long intergenic non-protein coding RNA 244	0.542766654	0.257724203	1.074504059
LOC100128881	NULL	0.887721694	0.406533549	1.126733039
LOC100129726	NULL	1.01919533	0.453533443	1.168149729
LOC101409256	cell division cycle 42 pseudogene	0.25031977	0.124307334	1.009860822
LOC1720	NULL	0.172637279	0.433316851	-1.327678309
LOC646862	NULL	0.402586663	0.201111055	1.001306993
LSMD1	NULL	1.152911367	0.529413504	1.122814708
LSP1P3	LSP1 pseudogene 3	0.361206744	0.165181213	1.128775219
MascRNA_menRNA	NULL	0.696580372	0.150453264	2.210974416
MASP2	MBL associated serine protease 2	2.311462206	1.144376547	1.014243937
MEG3_1	NULL	0.750850488	0.265018083	1.502434858
MIEN1	migration and invasion enhancer 1	0.517662601	0.25395276	1.027451941
Mir_361	NULL	0.309770848	0.109351169	1.502232744
Mir_576	NULL	0.173803157	0.348607204	-1.004148095
Mir_616	NULL	0.616369463	0.245922063	1.325594217
Mir_875	NULL	0.356975749	0.92131703	-1.367871613
MIR103A1	microRNA 103a-1	0.185560948	0.636596229	-1.778485488
MIR103B1	microRNA 103b-1	0.193722229	0.62383972	-1.687185907
MIR105-1	microRNA 105-1	0.064152523	0.201188843	-1.648972392
MIR106A	microRNA 106a	0.133170791	0.511345037	-1.941019413
MIR107	microRNA 107	1.088913132	0.476224515	1.193175073
MIR1181	microRNA 1181	0.688231072	0.140031259	2.297144118
MIR1229	microRNA 1229	1.406066986	0.493125873	1.511637473
MIR1237	microRNA 1237	0.419948053	0.187472649	1.16353075
MIR1264	microRNA 1264	0.059559466	0.526954315	-3.145275178
MIR1270-1	NULL	0.113552781	0.042845085	1.40616142
MIR1279	microRNA 1279	0.221708491	0.095816552	1.210317228
MIR1295A	microRNA 1295a	0.652877849	0.272924065	1.258313484
MIR1295B	microRNA 1295b	0.648919581	0.274475187	1.241363969
MIR135A2	microRNA 135a-2	0.222234807	0.090203463	1.300830064
MIR136	microRNA 136	0.282758084	0.098052453	1.527942648
MIR137	microRNA 137	0.956288642	0.362629443	1.398950076
MIR148B	microRNA 148b	0.418095814	0.19516596	1.099132153
MIR153-2	microRNA 153-2	0.291125972	0.713544103	-1.293359051
MIR183	microRNA 183	1.506968127	0.748603837	1.009374556
MIR186	microRNA 186	0.19033262	0.085622776	1.152456327
MIR190A	microRNA 190a	0.857606322	0.414791786	1.047928215
MIR191	microRNA 191	0.624779174	0.285665013	1.129022011
MIR1913	microRNA 1913	0.733921553	0.274299034	1.419876327
MIR193A	microRNA 193a	1.208278379	0.577695615	1.064571431
MIR194-2	microRNA 194-2	0.497973848	0.193972812	1.360215528
MIR197	microRNA 197	0.356119986	0.092169624	1.950000132
MIR199A1	microRNA 199a-1	0.783631986	0.340841238	1.201076389
MIR19B2	microRNA 19b-2	0.178043753	0.470144461	-1.400872304
MIR200A	microRNA 200a	0.954892422	0.363345586	1.393995828
MIR2052	microRNA 2052	0.879509152	0.356367981	1.303330873
MIR20B	microRNA 20b	0.28971095	0.685422797	-1.242379964
MIR211	microRNA 211	1.249426235	0.559880991	1.158073626
MIR2110	microRNA 2110	2.045074879	0.850433085	1.265884038
MIR212	microRNA 212	0.686090851	0.139014435	2.30316493
MIR218-1	microRNA 218-1	0.76459904	0.339056971	1.173175683
MIR25	microRNA 25	0.298685548	0.76222065	-1.351581262
MIR29B1	microRNA 29b-1	0.437036884	0.201977437	1.113560906
MIR3064	microRNA 3064	0.38750509	0.141862595	1.449721018
MIR30C2	microRNA 30c-2	0.155162911	0.360648019	-1.216807754
MIR3130-1	microRNA 3130-1	0.587879111	0.17859122	1.718858359

MIR3130-2	microRNA 3130-2	0.587879111	0.17859122	1.718858359
MIR3162	microRNA 3162	0.311359875	0.10107167	1.623204364
MIR3169	microRNA 3169	0.169717969	0.052795086	1.68466376
MIR3170	microRNA 3170	0.992337434	0.32908087	1.592388615
MIR3183	microRNA 3183	0.786447919	0.286448359	1.457076159
MIR3184	microRNA 3184	1.110854193	0.271160289	2.034451647
MIR3187	microRNA 3187	0.417418931	0.116385737	1.842581769
MIR3188	microRNA 3188	0.18069977	0.423584882	-1.229056432
MIR3189	microRNA 3189	0.321185687	0.148731033	1.110701904
MIR3193	microRNA 3193	0.752989952	0.232636742	1.694551643
MIR320A	microRNA 320a	0.578745099	1.240636439	-1.100080427
MIR320D2	microRNA 320d-2	0.281916542	0.131206997	1.103423475
MIR320E	microRNA 320e	0.209607537	0.098140606	1.094768516
MIR324	microRNA 324	0.471825874	0.225975584	1.062087632
MIR329-2	microRNA 329-2	0.392873787	0.166311955	1.240174033
MIR34A	microRNA 34a	0.654180466	0.260857639	1.326425999
MIR3615	microRNA 3615	1.125921549	0.416783865	1.433734975
MIR363	microRNA 363	0.325646821	0.128270713	1.344116331
MIR3649	microRNA 3649	0.603694372	0.289685498	1.059330887
MIR3650	microRNA 3650	0.263986255	0.576118883	-1.12590373
MIR3653	NULL	0.948438008	0.473148214	1.003261299
MIR3662	microRNA 3662	0.19136672	0.627760843	-1.713875088
MIR3677	microRNA 3677	0.534572658	0.225285656	1.246630592
MIR373	microRNA 373	0.166854786	0.650686182	-1.963368852
MIR374A	microRNA 374a	0.128055879	0.434489413	-1.762547542
MIR378D2	microRNA 378d-2	0.620712734	0.197168364	1.654497655
MIR378F	microRNA 378f	0.260187613	0.61944612	-1.251426523
MIR3911	microRNA 3911	0.350723197	0.140731555	1.317387012
MIR3914-1	microRNA 3914-1	0.577574484	0.273100562	1.080574728
MIR3914-2	microRNA 3914-2	0.581090973	0.273017558	1.089770311
MIR3916	microRNA 3916	0.287153821	0.118875959	1.272366783
MIR423	microRNA 423	1.093963163	0.275014753	1.991983243
MIR425	microRNA 425	0.308920149	0.714411333	-1.209520997
MIR4273	microRNA 4273	0.285409576	0.663733554	-1.21757047
MIR4275	microRNA 4275	0.165646594	0.406434463	-1.294914197
MIR4291	microRNA 4291	2.145544373	0.922589112	1.217583567
MIR4297	microRNA 4297	0.382437302	0.867646033	-1.181883349
MIR4305	microRNA 4305	0.134454217	0.441296334	-1.714632756
MIR4306	microRNA 4306	0.700487666	1.491329861	-1.090167843
MIR4321	microRNA 4321	0.519223136	0.19328286	1.425640961
MIR4322	microRNA 4322	0.357220105	0.129157793	1.467678592
MIR4328	microRNA 4328	0.045070344	0.13513924	-1.584196296
MIR4420	microRNA 4420	1.754909307	0.727106111	1.271158649
MIR4424	microRNA 4424	0.185753568	0.42846125	-1.205774809
MIR4427	microRNA 4427	0.277365767	1.992901168	-2.845008522
MIR4432	microRNA 4432	1.26226959	0.370898967	1.766921913
MIR4443	microRNA 4443	0.297533992	0.874457872	-1.555334384
MIR4444-1	microRNA 4444-1	1.146904119	0.55739091	1.040983409
MIR4456	microRNA 4456	1.295048381	0.457590079	1.500878317
MIR4457	microRNA 4457	0.251504569	0.750014744	-1.576334348
MIR4466	microRNA 4466	0.915025106	0.380698372	1.265162929
MIR4470	microRNA 4470	1.256564387	0.627575399	1.001623894
MIR4488	microRNA 4488	0.428038951	0.210568188	1.023454593
MIR449A	microRNA 449a	0.250512606	0.79843632	-1.672294146
MIR449B	microRNA 449b	0.280120823	0.883531725	-1.657232707
MIR450A2	microRNA 450a-2	0.256229228	0.119685127	1.098191173
MIR4517	microRNA 4517	0.144961387	0.539772541	-1.896682926
MIR4519	microRNA 4519	0.919548803	0.357820718	1.361689223
MIR451A	microRNA 451a	0.265170885	0.532848135	-1.006802033
MIR451B	microRNA 451b	0.265631601	0.544074285	-1.034376856
MIR4531	microRNA 4531	0.533543099	0.204197792	1.385637549
MIR4638	microRNA 4638	1.038483851	0.461060656	1.171450318
MIR4646	microRNA 4646	0.373476901	0.138625326	1.429828169
MIR4648	microRNA 4648	1.184117893	0.565732777	1.065620062
MIR4651	microRNA 4651	1.312822691	0.425442154	1.625637189
MIR4656	microRNA 4656	0.605635964	0.296927697	1.028339205
MIR4665	microRNA 4665	0.840897534	0.307365145	1.45197644
MIR4671	microRNA 4671	1.245379712	0.484182215	1.362963691
MIR4672	microRNA 4672	0.808017728	0.237570087	1.766033755
MIR4677	microRNA 4677	0.118597027	0.725082009	-2.612076333
MIR4684	microRNA 4684	0.640429455	0.31346079	1.03075467
MIR4703	microRNA 4703	0.614563949	0.206371483	1.574319509
MIR4716	microRNA 4716	0.402807525	0.146951556	1.454749998

MIR4721	microRNA 4721	0.36469955	0.174980712	1.05951252
MIR4725	microRNA 4725	0.801680109	0.395151282	1.02062159
MIR4728	microRNA 4728	0.406582503	0.151101795	1.428027332
MIR4735	microRNA 4735	0.629405241	0.305471826	1.042949867
MIR4736	microRNA 4736	2.014316699	0.882067509	1.191329545
MIR4738	microRNA 4738	0.513534908	0.107468096	2.256553922
MIR4741	microRNA 4741	0.937028159	0.327778801	1.515369851
MIR4747	microRNA 4747	0.376997935	0.160486319	1.232106304
MIR4749	microRNA 4749	0.584631129	0.200026952	1.547332246
MIR4753	microRNA 4753	0.432025489	0.086063692	2.327639803
MIR4766	microRNA 4766	0.126810019	0.425411183	-1.746189224
MIR4768	microRNA 4768	0.170488888	0.511448855	-1.584912266
MIR4769	microRNA 4769	0.241928248	0.557800983	-1.205171245
MIR4773-1	microRNA 4773-1	0.505928341	0.233419788	1.116006187
MIR4773-2	microRNA 4773-2	0.505928341	0.233419788	1.116006187
MIR4777	microRNA 4777	1.112664702	0.521056345	1.094507613
MIR4791	microRNA 4791	0.207252335	0.081166537	1.352431389
MIR4797	microRNA 4797	0.296800563	0.144362946	1.039793339
MIR487A	microRNA 487a	0.234439154	0.629106311	-1.4240903
MIR487B	microRNA 487b	0.652153629	0.263129938	1.309436459
MIR490	microRNA 490	0.351893782	0.769531371	-1.128840122
MIR496	microRNA 496	0.547298868	0.22723627	1.268135747
MIR497	microRNA 497	0.804567067	0.38971451	1.045795039
MIR5006	microRNA 5006	0.620576447	0.303759774	1.030678113
MIR5010	microRNA 5010	0.64845442	0.194138722	1.73991727
MIR504	microRNA 504	0.368954344	0.14817579	1.316132556
MIR5047	microRNA 5047	0.276246988	0.087578516	1.657309831
MIR506	microRNA 506	0.275466427	0.035561584	2.953485027
MIR507	microRNA 507	0.183774312	0.050771845	1.855834543
MIR508	microRNA 508	0.100577311	0.045625677	1.140387032
MIR510	microRNA 510	0.027934505	0.390425226	-3.804926136
MIR514B	microRNA 514b	0.258227013	0.123088227	1.068947149
MIR516B1	microRNA 516b-1	0.141430758	0.288378325	-1.02786683
MIR516B2	microRNA 516b-2	0.144364254	0.487943452	-1.757000407
MIR517B	microRNA 517b	0.248035087	0.07007006	1.823674189
MIR518E	microRNA 518e	0.235680235	0.101403704	1.216720427
MIR5193	microRNA 5193	0.637571145	0.316391848	1.010873909
MIR519D	microRNA 519d	0.088697451	0.418749888	-2.239124261
MIR520B	microRNA 520b	0.201089012	0.078676461	1.353830278
MIR520D	microRNA 520d	0.176116188	0.047264988	1.897683744
MIR520F	microRNA 520f	0.129141806	0.401765394	-1.637397195
MIR522	microRNA 522	0.116871687	0.241471065	-1.046924855
MIR523	microRNA 523	0.079675853	0.179104696	-1.168588696
MIR524	microRNA 524	0.08584382	0.222515215	-1.374117802
MIR527	microRNA 527	0.290830033	0.105387872	1.464467405
MIR539	microRNA 539	0.63308136	0.269993071	1.229468538
MIR542	microRNA 542	0.390184796	0.152004422	1.360044273
MIR545	microRNA 545	0.143960047	0.372097019	-1.370010354
MIR548F4	microRNA 548f-4	0.165594827	0.447292546	-1.433561111
MIR548J	microRNA 548j	1.045019103	0.516937547	1.015467415
MIR548M	microRNA 548m	0.241091307	0.511635585	-1.085536978
MIR553	microRNA 553	0.659295594	0.134895409	2.289084191
MIR558	microRNA 558	0.278710206	0.07852083	1.827618512
MIR5580	microRNA 5580	0.383161445	0.930944929	-1.280743428
MIR5586	microRNA 5586	0.17894171	0.089456444	1.000232388
MIR5587	microRNA 5587	1.386711684	0.388438284	1.835910562
MIR5687	microRNA 5687	0.498166691	1.475990347	-1.566982819
MIR5699	microRNA 5699	0.598118692	1.384738477	-1.211109824
MIR577	microRNA 577	0.277953421	0.715350951	-1.363808062
MIR592	microRNA 592	0.078115049	0.171146799	-1.131561901
MIR602	microRNA 602	0.225699719	0.636975241	-1.496832676
MIR624	microRNA 624	0.594113158	0.171603589	1.791658014
MIR626	microRNA 626	0.242656913	0.116017434	1.064576345
MIR630	microRNA 630	0.147274296	0.623357493	-2.081554127
MIR639	microRNA 639	0.261898837	0.711252523	-1.441352215
MIR643	microRNA 643	0.300114686	0.087786088	1.773449694
MIR645	microRNA 645	0.241157227	0.540775304	-1.165055225
MIR647	microRNA 647	0.441648803	0.178975156	1.303140264
MIR654	microRNA 654	0.360632997	0.176983107	1.026919744
MIR658	microRNA 658	0.261790815	0.577487009	-1.14137401
MIR7-1	microRNA 7-1	0.462418866	0.174617417	1.405002795
MIR761	microRNA 761	0.249501692	0.06570721	1.924926017
MIR764	microRNA 764	0.124572352	0.035836515	1.79748167

MIR765	microRNA 765	0.528573802	0.245512468	1.106308632
MIR769	microRNA 769	0.604267914	0.278324531	1.118420269
MIR890	microRNA 890	0.09656728	0.04503427	1.100511159
MIR891A	microRNA 891a	0.101348449	0.019713199	2.36209023
MIR9-3	microRNA 9-3	0.640528282	0.227661539	1.492371689
MIR937	microRNA 937	0.496154516	0.13353623	1.893558275
MIR938	microRNA 938	0.593628824	0.283564128	1.065886106
MIR939	microRNA 939	0.515594015	0.176386637	1.54749425
MIR941-3	microRNA 941-3	0.238044979	0.589896997	-1.309228866
MIR98	microRNA 98	0.16153786	0.058081319	1.475726223
MIR99B	microRNA 99b	0.567650445	1.183363599	-1.059818717
MIRLET7F1	microRNA let-7f-1	0.596238306	0.273717851	1.123199539
MIRLET7F2	microRNA let-7f-2	0.365954983	0.150053864	1.286185723
MIRLET7G	microRNA let-7g	0.187196999	0.392972306	-1.069870338
MRP63	NULL	1.00225362	0.444271676	1.173733556
MRPL51	mitochondrial ribosomal protein L51	0.637077603	0.295775937	1.106964434
NEUROD2	neuronal differentiation 2	0.732702159	0.304486063	1.266850675
NIP7	nucleolar pre-rRNA processing protein NIP7	0.778351663	0.289547233	1.426623409
NPBWR1	neuropeptides B and W receptor 1	0.549879967	0.243764171	1.173630639
NUDT17	nudix hydrolase 17	0.444040153	0.215373684	1.043848159
OR14C36	olfactory receptor family 14 subfamily C member 36	0.31633842	0.065411537	2.273851765
OR14I1	olfactory receptor family 14 subfamily I member 1	0.091386589	0.211154465	-1.208244384
OR14J1	olfactory receptor family 14 subfamily J member 1	0.155567203	0.375349962	-1.270698399
OR1F1	olfactory receptor family 1 subfamily F member 1	0.546988407	0.272166839	1.007018961
OR2AK2	olfactory receptor family 2 subfamily AK member 2	0.191291561	0.064425858	1.570061486
OR2H1	olfactory receptor family 2 subfamily H member 1	0.792176327	1.818024904	-1.198478469
OR2M1P	olfactory receptor family 2 subfamily M member 1 pseudogene	0.300025418	0.142925868	1.069817683
OR2M3	olfactory receptor family 2 subfamily M member 3	0.276317251	0.107431767	1.362904981
OR2M4	olfactory receptor family 2 subfamily M member 4	0.271559801	0.108702761	1.320881355
OR2T33	olfactory receptor family 2 subfamily T member 33	0.379005546	0.124431864	1.606862985
OR2Z1	olfactory receptor family 2 subfamily Z member 1	0.480029518	0.205894041	1.221221049
OR4A16	olfactory receptor family 4 subfamily A member 16	0.281159518	0.042155109	2.73760949
OR4C11	olfactory receptor family 4 subfamily C member 11	0.093408035	0.010728147	3.122145805
OR4C6	olfactory receptor family 4 subfamily C member 6	0.21967262	0.064820155	1.760840685
OR4F21	olfactory receptor family 4 subfamily F member 21	0.272655163	0.592008224	-1.118539743
OR4K15	olfactory receptor family 4 subfamily K member 15	0.106519703	0.364720207	-1.775669827
OR4N4	olfactory receptor family 4 subfamily N member 4	0.107492836	0.219750421	-1.031625423
OR4Q3	olfactory receptor family 4 subfamily Q member 3	0.189335886	0.386280334	-1.02870035
OR52Z1	NULL	1.223198969	0.475228338	1.363966324
OR5AK4P	olfactory receptor family 5 subfamily AK member 4 pseudogene	0.166760745	0.480966104	-1.528155506
OR5D18	olfactory receptor family 5 subfamily D member 18	0.197426705	0.09845184	1.003827079
OR5H1	olfactory receptor family 5 subfamily H member 1	0.167423637	0.49141914	-1.553450826
OR5J2	olfactory receptor family 5 subfamily J member 2	0.11039818	0.045143273	1.290133462
OR5L1	olfactory receptor family 5 subfamily L member 1	0.195442803	0.043612919	2.163919015
OR5M10	olfactory receptor family 5 subfamily M member 10	0.069018213	0.173419176	-1.32921442
OR5M11	olfactory receptor family 5 subfamily M member 11	0.108658757	0.277769356	-1.354083006
OR5T1	olfactory receptor family 5 subfamily T member 1	0.119165911	0.041143543	1.534233653
OR5T2	olfactory receptor family 5 subfamily T member 2	0.092571259	0.280778089	-1.600794114
OR6C65	olfactory receptor family 6 subfamily C member 65	0.190774476	0.484353518	-1.344192256
OR6C68	olfactory receptor family 6 subfamily C member 68	0.209969343	0.426277388	-1.021613833
OR7E24	olfactory receptor family 7 subfamily E member 24	0.156319373	0.338517419	-1.114733491
OR8J3	olfactory receptor family 8 subfamily J member 3	0.16930413	0.056774041	1.576313842
OR8K3	olfactory receptor family 8 subfamily K member 3 (gene/pseudogene)	0.092886201	0.204638612	-1.139542195
OR9G4	olfactory receptor family 9 subfamily G member 4	0.175721269	0.079252246	1.148765106
PCDHB18	NULL	0.214897823	0.094932949	1.17867006
PCDHX	NULL	0.009919532	0.03311295	-1.739051583
PDCL3P4	PDCL3 pseudogene 4	0.24366758	0.084139119	1.534065702
PDF	peptide deformylase, mitochondrial	0.61928814	0.292103039	1.084133449
PGP	phosphoglycolate phosphatase	0.423291548	0.206285721	1.037007724
POLD4	DNA polymerase delta 4, accessory subunit	3.208054985	1.590855883	1.011895723
PQBP1	polyglutamine binding protein 1	3.766953405	1.788130621	1.074946061
PYDC1	pyrin domain containing 1	1.213758852	0.601880539	1.011932744
RMRP	RNA component of mitochondrial RNA processing endoribonuclease	0.796725383	0.367708408	1.115520374
RNA5SP411	RNA, 5S ribosomal pseudogene 411	0.317537196	0.137250698	1.210112114
RNU6-16P	RNA, U6 small nuclear 16, pseudogene	0.223913203	0.590166716	-1.398182958
RNU6-64P	RNA, U6 small nuclear 64, pseudogene	0.080479749	0.612931684	-2.929028581
RNU6-67P	RNA, U6 small nuclear 67, pseudogene	0.170662712	0.447832129	-1.391810161
RNU6-78P	RNA, U6 small nuclear 78, pseudogene	0.443731313	0.171494526	1.371523837
RPL29P2	ribosomal protein L29 pseudogene 2	0.440943687	0.140337272	1.651696197
RPP25	ribonuclease P and MRP subunit p25	0.47541131	0.221773572	1.100088772
RPS27	ribosomal protein S27	0.33827912	0.154250624	1.132937808
RPS27A	ribosomal protein S27a	1.45941965	0.668506036	1.126382293
RPSAP9	ribosomal protein SA pseudogene 9	0.665134308	0.31235052	1.090479758

SAC3D1	SAC3 domain containing 1	0.485131823	0.167401426	1.535065005
SCARNA10	small Cajal body-specific RNA 10	0.209179644	0.460738963	-1.139207142
SCARNA14	small Cajal body-specific RNA 14	0.118587637	0.30978275	-1.385303203
SCARNA18	small Cajal body-specific RNA 18	0.135436711	0.275486986	-1.024365326
SCARNA6	small Cajal body-specific RNA 6	0.942537635	0.443095802	1.088931566
SCGB2B3P	secretoglobin family 2B member 3, pseudogene	1.569314285	0.494682193	1.665560435
SCXB	NULL	0.48539361	0.241030287	1.009940674
SLC16A11	solute carrier family 16 member 11	0.546397829	0.264812359	1.044981295
SMIM3	small integral membrane protein 3	0.46614887	0.934711928	-1.003731036
SnoMe28S_Am2634	NULL	0.049451873	0.163541574	-1.725560357
SNORA12	small nucleolar RNA, H/ACA box 12	0.223466675	0.09815101	1.186984691
SNORA16	NULL	0.189796616	0.405609684	-1.095637826
SNORA16A	small nucleolar RNA, H/ACA box 16A	0.407160809	0.16684651	1.287077191
SNORA18	small nucleolar RNA, H/ACA box 18	0.153178977	0.356305885	-1.217898004
SNORA23	small nucleolar RNA, H/ACA box 23	0.451836405	0.137022394	1.721388817
SNORA28	small nucleolar RNA, H/ACA box 28	0.348057527	0.091038695	1.934774
SNORA34	NULL	0.332017106	0.151579024	1.131187453
SNORA35	small nucleolar RNA, H/ACA box 35	1.098890339	0.472234837	1.218471044
SNORA38B	small nucleolar RNA, H/ACA box 38B	0.090718928	0.361877541	-1.996026074
SNORA41	small nucleolar RNA, H/ACA box 41	0.471478036	0.991845279	-1.072924524
SNORA42	NULL	0.218398668	0.077389906	1.496746751
SNORA44	small nucleolar RNA, H/ACA box 44	0.409972091	0.171141689	1.260334467
SNORA61	small nucleolar RNA, H/ACA box 61	0.316362124	0.135450486	1.223811316
SNORA68	small nucleolar RNA, H/ACA box 68	0.705112229	0.254248482	1.471613742
SNORA70B	small nucleolar RNA, H/ACA box 70B	0.304496254	0.644974593	-1.082817852
SNORA75	small nucleolar RNA, H/ACA box 75	2.855664825	1.303753907	1.131155081
SNORD111	small nucleolar RNA, C/D box 111	0.128427241	0.059528689	1.109294221
SNORD113-6	small nucleolar RNA, C/D box 113-6	0.068797307	0.192203605	-1.482211408
SNORD113-7	small nucleolar RNA, C/D box 113-7	0.194911689	0.05935231	1.715444535
SNORD113-9	small nucleolar RNA, C/D box 113-9	0.094841887	0.284191843	-1.583268874
SNORD114-14	small nucleolar RNA, C/D box 114-14	0.051285333	0.165347011	-1.688878765
SNORD114-15	small nucleolar RNA, C/D box 114-15	0.091193827	0.412638753	-2.177871251
SNORD114-16	small nucleolar RNA, C/D box 114-16	0.04560629	0.366841903	-3.007853737
SNORD114-18	small nucleolar RNA, C/D box 114-18	0.076935095	0.164356191	-1.095112043
SNORD114-19	small nucleolar RNA, C/D box 114-19	0.047016656	0.197640475	-2.071634587
SNORD114-2	small nucleolar RNA, C/D box 114-2	0.107135548	0.047643724	1.169079156
SNORD114-23	small nucleolar RNA, C/D box 114-23	0.194102807	0.059850333	1.697389799
SNORD114-26	small nucleolar RNA, C/D box 114-26	0.111314944	0.052400819	1.086986035
SNORD114-27	small nucleolar RNA, C/D box 114-27	0.083023122	0.166483027	-1.003790012
SNORD114-30	small nucleolar RNA, C/D box 114-30	0.182791975	0.069727701	1.39039892
SNORD114-4	small nucleolar RNA, C/D box 114-4	0.265109674	0.054647094	2.278372642
SNORD115-3	small nucleolar RNA, C/D box 115-3	0.19515592	0.574785596	-1.55839668
SNORD115-30	small nucleolar RNA, C/D box 115-30	0.239300193	0.575495911	-1.265984117
SNORD115-4	small nucleolar RNA, C/D box 115-4	0.413065443	0.17879358	1.208075439
SNORD115-47	small nucleolar RNA, C/D box 115-47	0.148854038	0.059082532	1.333094811
SNORD116-1	small nucleolar RNA, C/D box 116-1	0.090093666	0.334118176	-1.890860879
SNORD116-28	small nucleolar RNA, C/D box 116-28	0.100107328	0.366930365	-1.873958711
SNORD116-29	small nucleolar RNA, C/D box 116-29	0.066883926	0.331467121	-2.309134345
SNORD116-4	small nucleolar RNA, C/D box 116-4	0.22059882	0.473179087	-1.100961239
SNORD116-5	small nucleolar RNA, C/D box 116-5	0.180572916	0.395789346	-1.132151262
SNORD117	small nucleolar RNA, C/D box 117	0.362048161	0.088989571	2.024473452
SNORD11B	small nucleolar RNA, C/D box 11B	0.642587219	0.148507861	2.113352985
SNORD126	small nucleolar RNA, C/D box 126	0.061698461	0.376631406	-2.609846896
SNORD127	small nucleolar RNA, C/D box 127	0.177954402	0.073805231	1.26971264
SNORD12C	small nucleolar RNA, C/D box 12C	0.738844249	0.223900393	1.722413211
SNORD14	NULL	0.678147234	0.192515025	1.81662749
SNORD15A	small nucleolar RNA, C/D box 15A	0.851081145	0.388422788	1.131668849
SNORD16	small nucleolar RNA, C/D box 16	0.45313403	0.17535937	1.369623318
SNORD18A	small nucleolar RNA, C/D box 18A	0.310213053	0.129520902	1.26007446
SNORD18C	small nucleolar RNA, C/D box 18C	0.547914635	0.267772525	1.032943201
SNORD1B	small nucleolar RNA, C/D box 1B	0.215963438	0.455764632	-1.077501883
SNORD1C	small nucleolar RNA, C/D box 1C	0.622945285	0.251047661	1.311144169
SNORD2	small nucleolar RNA, C/D box 2	0.671819582	0.224196245	1.583311736
SNORD25	small nucleolar RNA, C/D box 25	0.812948853	0.393304435	1.047518134
SNORD26	small nucleolar RNA, C/D box 26	0.336610319	0.734154141	-1.125003594
SNORD29	small nucleolar RNA, C/D box 29	0.565393776	0.163770078	1.78758421
SNORD36C	small nucleolar RNA, C/D box 36C	0.389277716	0.183068282	1.088417904
SNORD42A	small nucleolar RNA, C/D box 42A	0.277314136	0.137992499	1.006931317
SNORD49B	small nucleolar RNA, C/D box 49B	1.076032879	0.503382047	1.095996493
SNORD5	small nucleolar RNA, C/D box 5	0.663818046	1.482141985	-1.158823906
SNORD50	NULL	0.38669657	0.074749359	2.371068863
SNORD53	small nucleolar RNA, C/D box 53	0.195579056	0.090296841	1.115004459
SNORD59A	small nucleolar RNA, C/D box 59A	0.411119288	0.102166267	2.008638136

SNORD62A	small nucleolar RNA, C/D box 62A	1.25438123	0.560192245	1.162981961
SNORD67	small nucleolar RNA, C/D box 67	0.553386743	0.253283488	1.127535058
SNORD69	small nucleolar RNA, C/D box 69	0.369621827	0.083153402	2.15220276
SNORD76	small nucleolar RNA, C/D box 76	1.264216675	0.547938994	1.206156567
SNORD79	small nucleolar RNA, C/D box 79	0.982300158	0.328904269	1.578496197
SNORD8	small nucleolar RNA, C/D box 8	0.173601118	0.398627018	-1.199263259
SNORD85	NULL	0.235680269	0.096895598	1.282327959
SNORD88B	small nucleolar RNA, C/D box 88B	0.580263496	0.237777592	1.28709541
SNORD88C	small nucleolar RNA, C/D box 88C	0.937638484	0.320121829	1.550410727
SNORD89	small nucleolar RNA, C/D box 89	0.510677057	0.191648623	1.413947616
SNORD91A	small nucleolar RNA, C/D box 91A	0.075078083	0.169606175	-1.175724987
SNORD92	small nucleolar RNA, C/D box 92	0.276002291	0.088647192	1.63853341
SnoU83B	NULL	0.176590932	0.553308586	-1.647673048
SPHK1	sphingosine kinase 1	3.15312412	1.526254243	1.046786656
SSSCA1	NULL	0.520497383	0.208529357	1.319640411
SSSCA1-AS1	NULL	0.595570728	0.184572599	1.690084459
STH	saitohin	0.793095572	0.369088389	1.103528376
SUMO4	small ubiquitin like modifier 4	0.366335724	0.125676819	1.543447825
T-cell receptor alpha chain variable ...	NULL	1.203110759	0.584387736	1.041771658
TCRAVN1	NULL	0.237809859	0.668900649	-1.491983421
TGIF2LX	TGFB induced factor homeobox 2 like X-linked	0.126410463	0.059456063	1.08822004
TGIF2LY	TGFB induced factor homeobox 2 like Y-linked	0.12096651	0.04140288	1.546804651
TIMM22	translocase of inner mitochondrial membrane 22	0.409961278	0.187028163	1.132232124
TMEM141	transmembrane protein 141	1.097629736	0.517549535	1.084622616
TMEM187	transmembrane protein 187	0.650070799	0.317808799	1.032437781
TMEM191C	transmembrane protein 191C	1.338223164	0.61833419	1.113860038
TMEM88	transmembrane protein 88	0.432204208	0.190388108	1.182769754
TMSB10	thymosin beta 10	0.514409321	0.173055937	1.57167835
TOB2	transducer of ERBB2, 2	0.645091938	0.277193664	1.218610508
U39094	NULL	0.600473997	1.456448796	-1.278281304
UBXN1	UBX domain protein 1	1.593083833	0.746138033	1.094307734
UNQ599/PRO1185	NULL	0.460853374	0.226618855	1.024039914
VN1R1	vomeronasal 1 receptor 1	0.173459953	0.071185406	1.284949216
VTRNA1-2	vault RNA 1-2	0.23463207	0.485079951	-1.047822333
WFIKKN1	WAP, follistatin/kazal, immunoglobulin, kunitz and netrin domain containing 1	0.607387083	0.297531931	1.02957172
X15673	NULL	0.463170852	0.223355797	1.05220077
Y16709	NULL	0.379743671	0.134967878	1.492409833
YIPF2	Yip1 domain family member 2	0.84865624	0.409221992	1.052296609
ZACN	zinc activated ion channel	1.796560841	0.894541113	1.006018098
ZBED6	zinc finger BED-type containing 6	0.349981857	0.159837791	1.130671583
ZNF835	zinc finger protein 835	0.681480186	0.337971324	1.011770869
